# Supplementary material for: CellNeighborEX: deciphering neighbor‐dependent gene expression from spatial transcriptomics data
Source: Mol Syst Biol. 2023 Oct 10;19(11):e11670. doi: 10.15252/msb.202311670 (PMC10632736; doi:10.15252/msb.202311670)
Supplement: Supplementary file 1 — Appendix S1 [file MSB-19-e11670-s008.pdf]

# CellNeighborEX: Deciphering Neighbor-Dependent Gene Expression from Spatial Transcriptomics Data

## Appendix Figures

### Table of Contents

1. Appendix Figure S1 - Generation of artificial heterotypic spots for statistical tests…………2
2. Appendix Figure S2 – Validation of cell types of heterotypic spots…………………………..3
3. Appendix Figure S3 - Regression models to determine the origin of the expression of neighbor-dependent genes……………………………………………………………………..4
4. Appendix Figure S4 - Comparison of neighbor-dependent genes to ligand-receptor pairs & downstream targets…………………………………………………………………………6
5. Appendix Figure S5 - Distributions of minimum distances between two interacting cell types detected by NicheNet………………………………………………………………………..7
6. Appendix Figure S6 - Spatial plots with cell identities in Slide-seq data……………………8
7. Appendix Figure S7 - Heterogeneity of Interneuron (In) cells in the mouse hippocampus Slide-seq data…………………………………………………………………………………...10
8. Appendix Figure S8 - UMAP projections for single cell/nucleus data……………………..12
9. Appendix Figure S9 - Origins of the expression of neighbor-dependent genes identified from single cell/nucleus data……………………………………………………………………..14

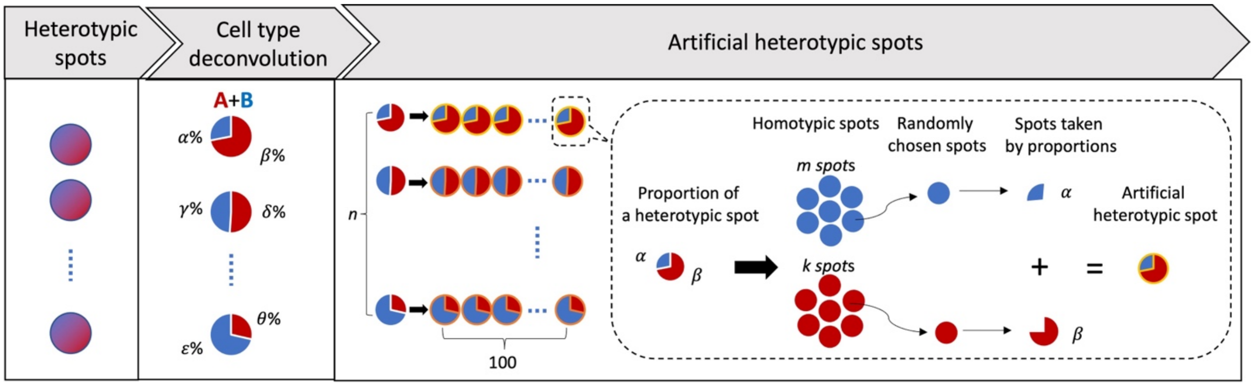


**Appendix Figure S1. Generation of artificial heterotypic spots for statistical tests.** Artificial heterotypic spots are produced by combining two different cell types of homotypic spots. For the two cell types, a homotypic spot is randomly chosen respectively and then their transcriptomes are mixed according to the cell type proportions of the heterotypic spots. From repeated random sampling, 100 artificial heterotypic spots are created for each heterotypic spot.

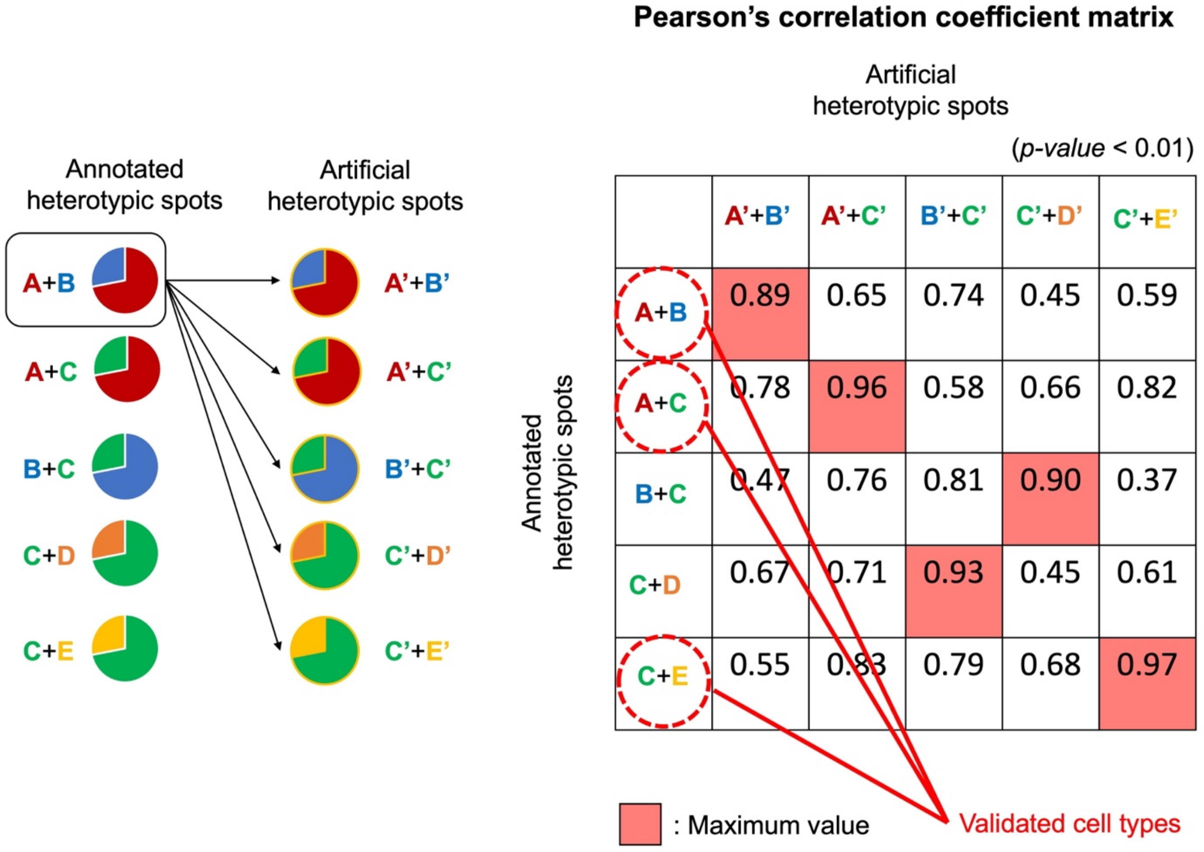


**Appendix Figure S2. Validation of cell types of heterotypic spots.**

Correlation analysis is performed to validate if the cell types of the heterotypic spots annotated by RCTD are correct. The Pearson's correlation coefficients are calculated based on the gene expression values between true heterotypic spots and many combinatorial types of artificial heterotypic spots. In the case that the cell type of true heterotypic spots matches that of artificial heterotypic spots with the largest Pearson's coefficient, the cell type annotation is regarded as validated.

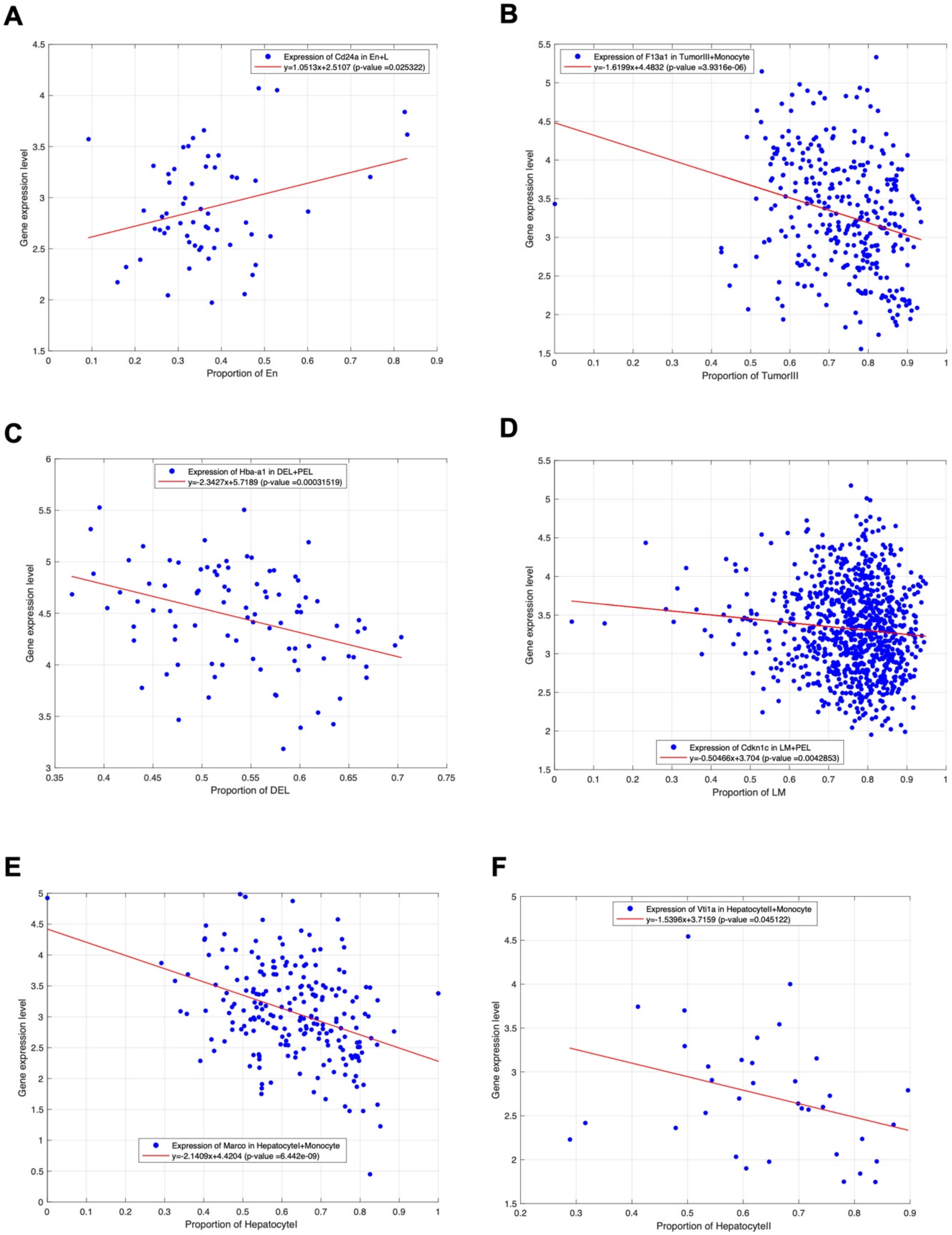
A
Expression of Cd24a in En+L
y=1.0513x+2.5107 (p-value =0.025322)
Gene expression level
Proportion of En
B
Expression of F13a1 in TumorIII+Monocyte
y=-1.6199x+4.4832 (p-value =3.9316e-06)
Gene expression level
Proportion of TumorIII
C
Expression of Hba-a1 in DEL+PEL
y=-2.3427x+5.7189 (p-value =0.00031519)
Gene expression level
Proportion of DEL
D
Expression of Cdkn1c in LM+PEL
y=-0.50466x+3.704 (p-value =0.0042853)
Gene expression level
Proportion of LM
E
Expression of Marco in HepatocyteI+Monocyte
y=-2.1409x+4.4204 (p-value =6.442e-09)
Gene expression level
Proportion of HepatocyteI
F
Expression of Vti1a in HepatocyteII+Monocyte
y=-1.5396x+3.7159 (p-value =0.045122)
Gene expression level
Proportion of HepatocyteII

**Appendix Figure S3. Regression models to determine the origin of the expression of neighbor-dependent genes.**

A The expression of *Cd24a* in the heterotypic spots of Endothelial and Lens cells (En+L). X-axis indicates the ratio of Endothelial cells against Lens cells in the heterotypic spots. The positive relationships indicate that *Cd24a* is expressed in Endothelial cells.

B The expression levels of *F13a1* expression in the heterotypic spots of Tumor III and Monocyte cells (Tumor III+Monocyte). It suggests that the expression of *F13a1* is expressed in Monocyte cells.

C The expression of Hba-a1 in the heterotypic spots of Definitive erythroid lineage and Primitive erythroid lineage cells (DEL+PEL). It suggests that *Hba-a1* is expressed in PEL.

D The expression levels of *Cdkn1c* in the heterotypic spots of Limb mesenchyme and Primitive erythroid lineage cells (LM+PEL). It suggests that the expression of *Cdkn1c* is expressed in PEL.

E The expression levels of *Marco* in the heterotypic spots of Hepatocyte I and Monocyte cells (Hepatocyte I+Monocyte). It suggests that the expression of *Marco* is expressed in Monocyte cells.

F The expression levels of *Vti1a* in the heterotypic spots of Hepatocyte II and Monocyte cells (Hepatocyte II+Monocyte). It suggests that the expression of *Vti1a* is expressed in Monocyte cells.

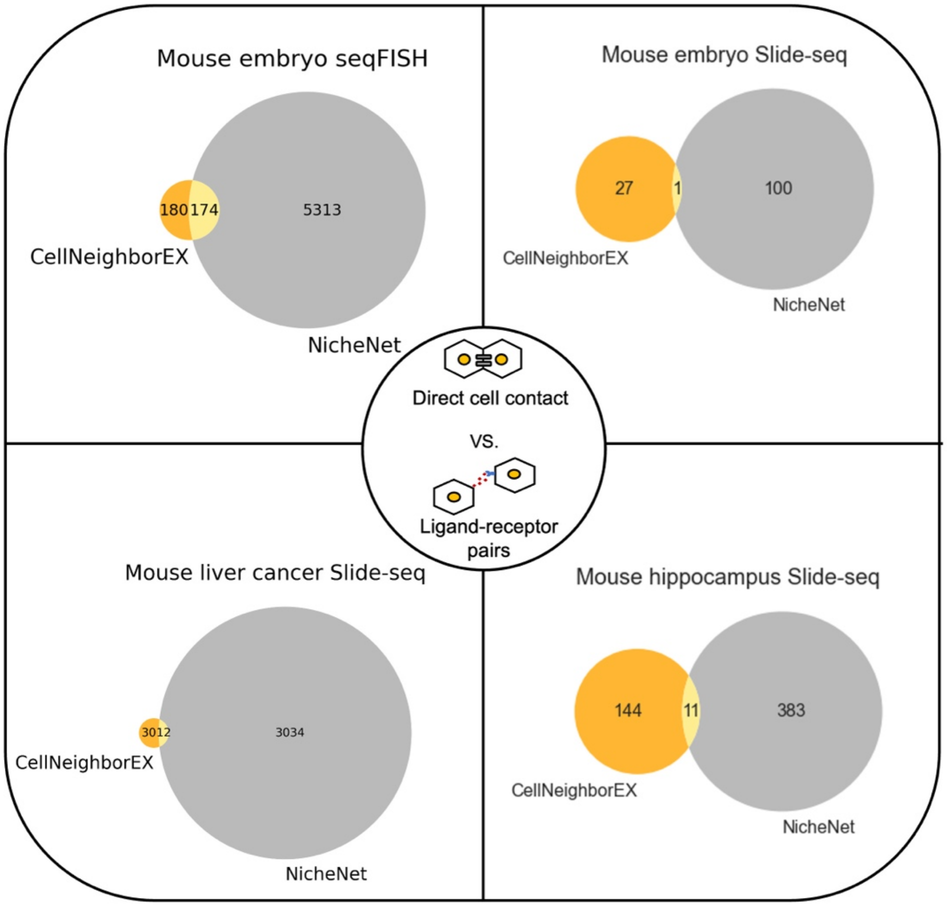


**Appendix Figure S4. Comparison of neighbor-dependent genes to ligand-receptor pairs & downstream targets.**

In Venn diagrams, "CellNeighborEX" represents genes up-regulated by cell contact while "NicheNet" indicates ligand-receptor pairs and their downstream target genes.

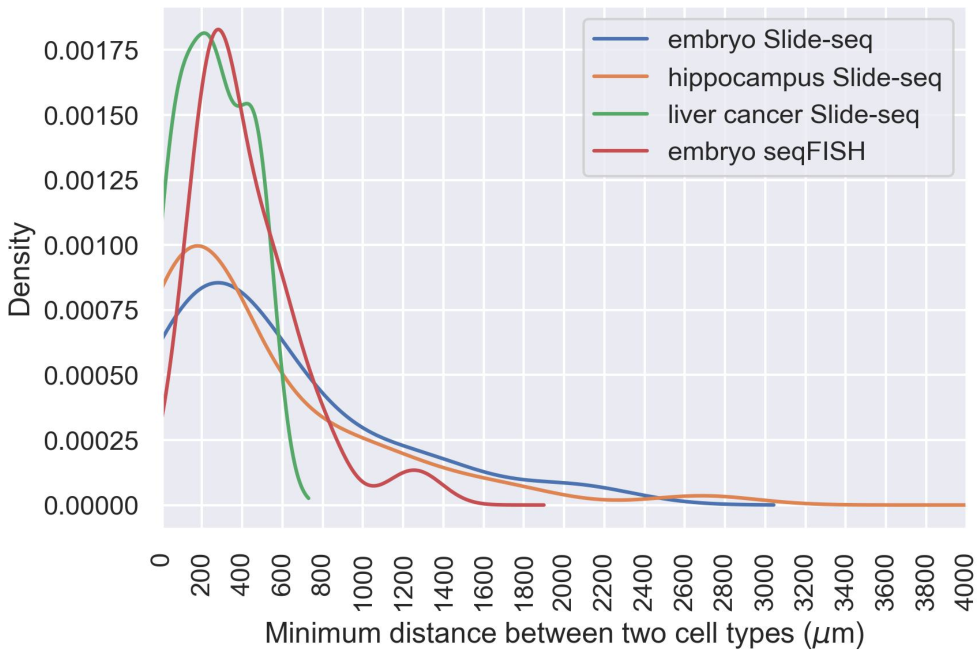


**Appendix Figure S5. Distributions of minimum distances between two interacting cell types detected by NicheNet.**

The minimum distance is defined as a distance from a sender cell to the nearest receiver cell between two interacting cell types identified by NicheNet. For each sender cell, the minimum distance is calculated and then the distance values are averaged. The distribution of each dataset is obtained from the averaged minimum distances for the entire cell type pairs of a sender and a receiver. The distributions are estimated by kernel density estimation.

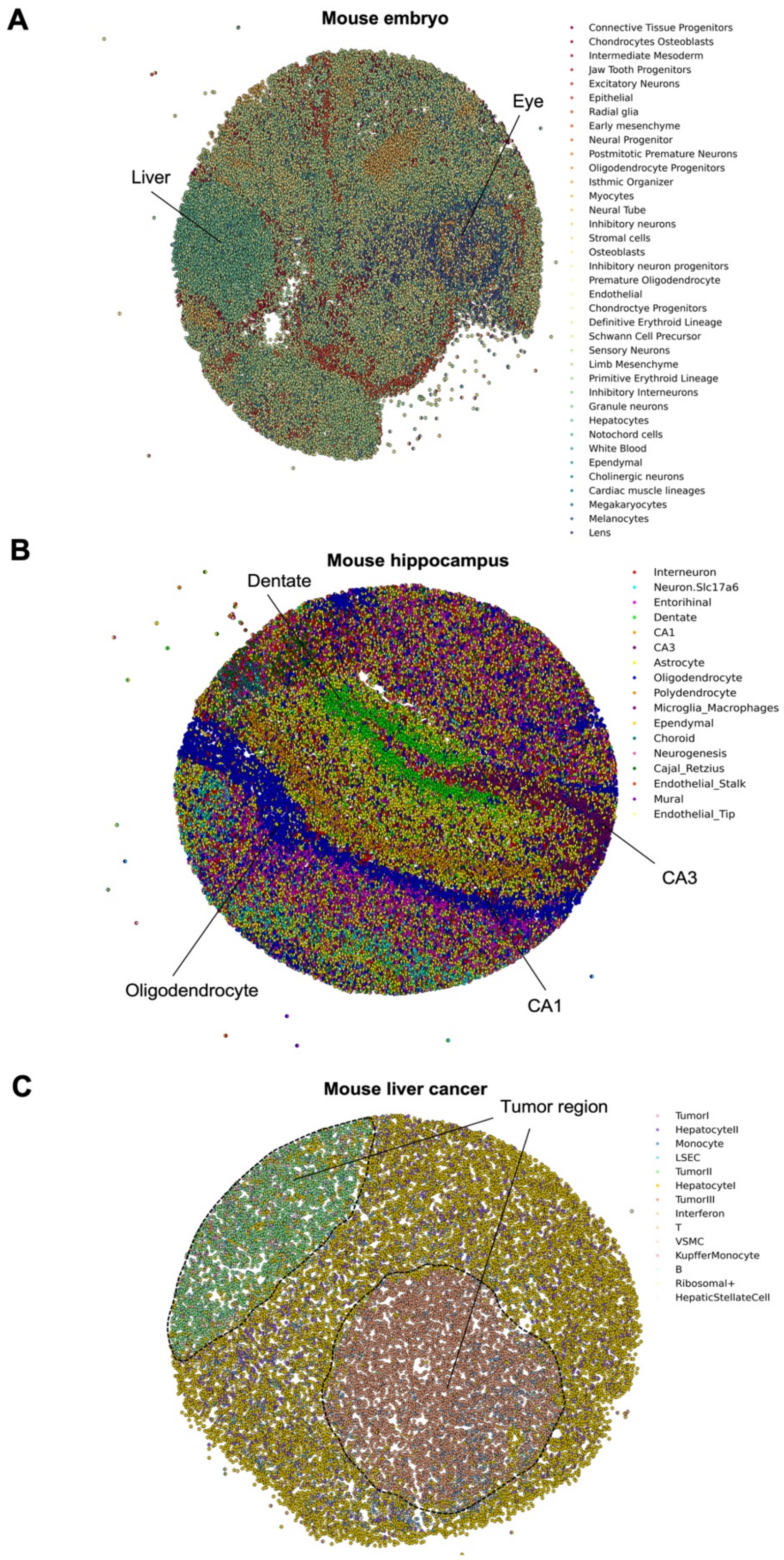
A
Mouse embryo
Eye
Liver
Connective Tissue Progenitors
Chondrocytes Osteoblasts
Intermediate Mesoderm
Jaw Tooth Progenitors
Excitatory Neurons
Epithelial
Radial glia
Early mesenchyme
Neural Progenitor
Postmitotic Premature Neurons
Oligodendrocyte Progenitors
Isthmic Organizer
Myocytes
Neural Tube
Inhibitory neurons
Stromal cells
Osteoblasts
Inhibitory neuron progenitors
Premature Oligodendrocyte
Endothelial
Chondroctye Progenitors
Definitive Erythroid Lineage
Schwann Cell Precursor
Sensory Neurons
Limb Mesenchyme
Primitive Erythroid Lineage
Inhibitory Interneurons
Granule neurons
Hepatocytes
Notochord cells
White Blood
Ependymal
Cholinergic neurons
Cardiac muscle lineages
Megakaryocytes
Melanocytes
Lens
B
Mouse hippocampus
Dentate
CA3
Oligodendrocyte
CA1
Interneuron
Neuron.Slc17a6
Entorihinal
Dentate
CA1
CA3
Astrocyte
Oligodendrocyte
Polydendrocyte
Microglia_Macrophages
Ependymal
Choroid
Neurogenesis
Cajal_Retzius
Endothelial_Stalk
Mural
Endothelial_Tip
C
Mouse liver cancer
Tumor region
TumorI
HepatocyteII
Monocyte
LSEC
TumorII
HepatocyteI
TumorIII
Interferon
T
VSMC
KupfferMonocyte
B
Ribosomal+
HepaticStellateCell

**Appendix Figure S6. Spatial plots with cell identities in Slide-seq data.**

A In the mouse embryo Slide-seq data, the spots are composed of 37 cell types.

B In the mouse hippocampus Slide-seq data, the spots have 17 cell types.

C In the mouse liver cancer Slide-seq data, the spots of the liver cancer data consist of 14 cell types.

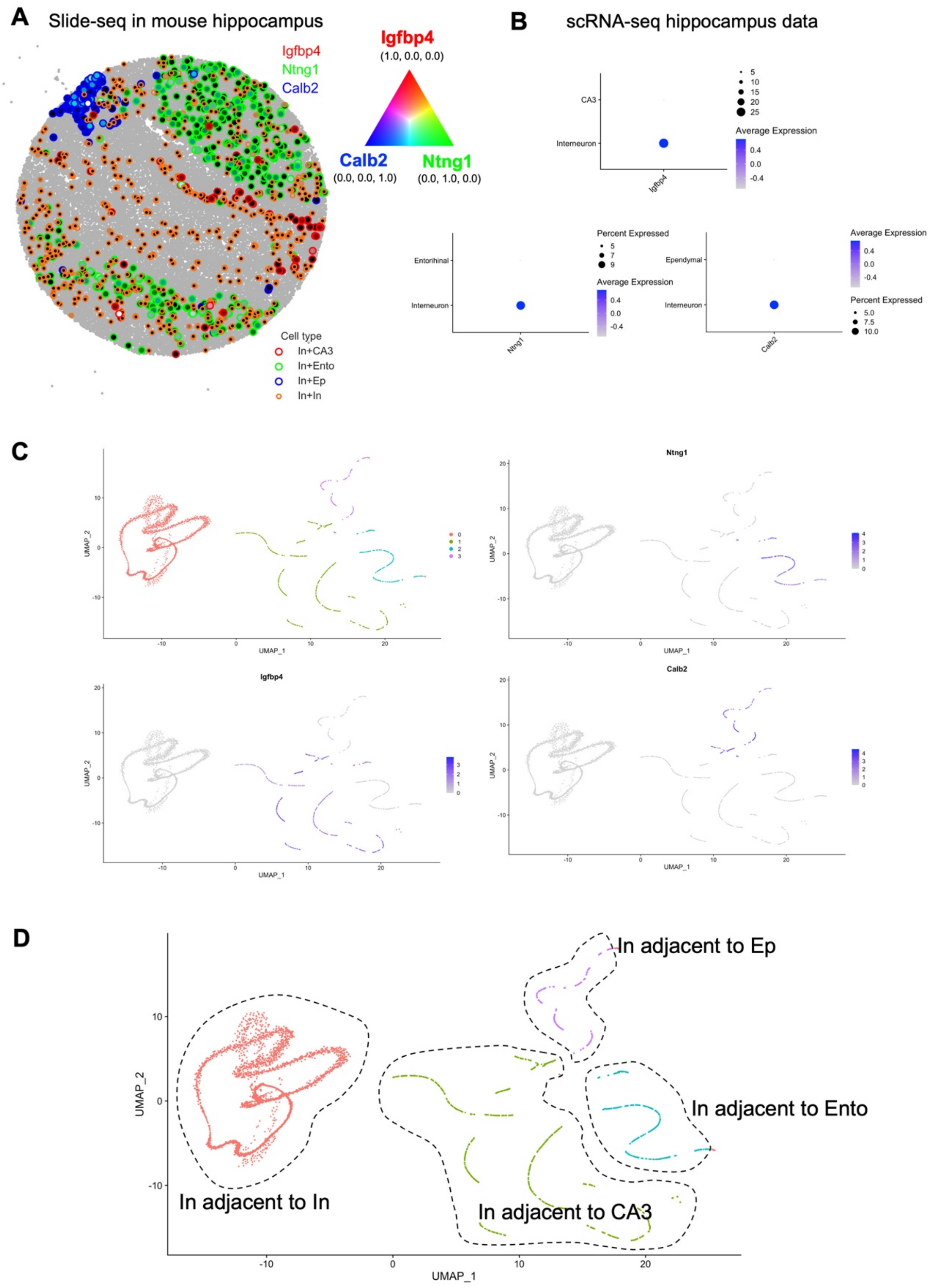
A
Slide-seq in mouse hippocampus
Igfbp4
Ntng1
Calb2
Igfbp4
(1.0, 0.0, 0.0)
Calb2
(0.0, 0.0, 1.0)
Ntng1
(0.0, 1.0, 0.0)
Cell type
In+CA3
In+Ento
In+Ep
In+In
B
scRNA-seq hippocampus data
CA3
Interneuron
Igfbp4
Average Expression
Entorhinal
Interneuron
Ntng1
Percent Expressed
Average Expression
Ependymal
Interneuron
Calb2
Average Expression
Percent Expressed
C
Ntng1
Igfbp4
Calb2
UMAP_1
UMAP_2
D
In adjacent to Ep
In adjacent to Ento
In adjacent to In
In adjacent to CA3
UMAP_1
UMAP_2

**Appendix Figure S7. Heterogeneity of Interneuron (In) cells in the mouse hippocampus Slide-seq data.**

A Neighboring cell type-dependent gene expression of Interneuron cells. Interneuron cells dominantly express *Igfbp4* (red) when proximal to CA3 (In+CA3), *Ntng1* (green) when proximal to Entorhinal cells (In+Ento), and *Calb2* (blue) when proximal to Ependymal cells (In+Ep).

B Expression of neighbor-dependent genes in the mouse hippocampus scRNA-seq data. It was confirmed that the three genes are mostly expressed from Interneuron cells.

C UMAP of Interneuron cells. 4 clusters were obtained from clustering analysis: Cluster 0 to 3. *Igfbp4* is mostly expressed in Cluster 1, *Ntng1* in Cluster 2, *Calb2* in Cluster 3, and none of them is expressed in Cluster 0.

D Heterogeneity of Interneuron cells explained by niche-specific gene expression. It can be inferred from (C) that Cluster 1 is Interneuron cells adjacent to CA3, Cluster 2 is Interneuron adjacent to Ento, Cluster 3 is Interneuron adjacent to Ep, and Cluster 0 is Interneuron adjacent to another Interneuron.

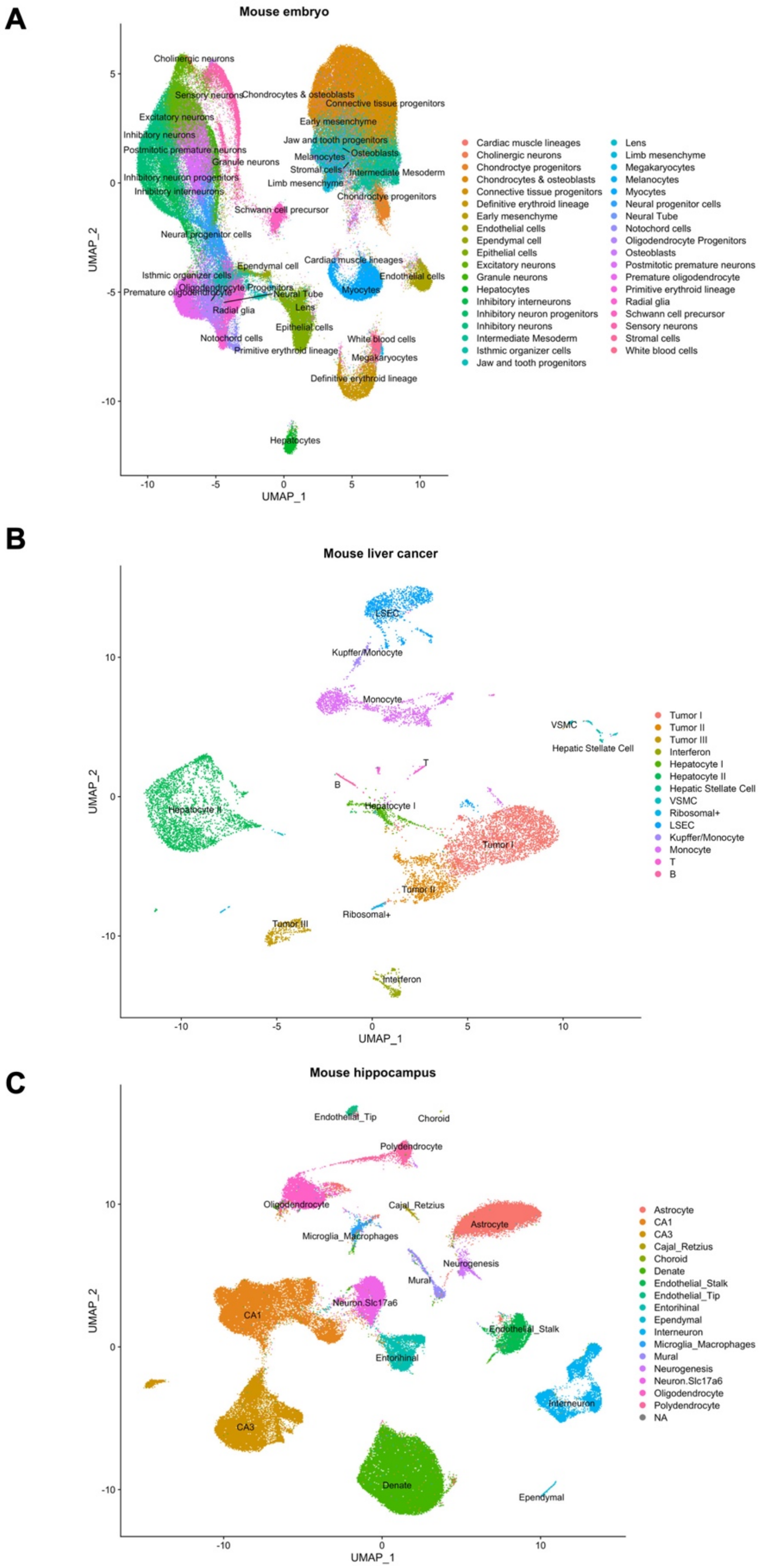
A
Mouse embryo
UMAP_1
UMAP_2
Cardiac muscle lineages
Cholinergic neurons
Chondrocyte progenitors
Chondrocytes & osteoblasts
Connective tissue progenitors
Definitive erythroid lineage
Early mesenchyme
Endothelial cells
Ependymal cell
Epithelial cells
Excitatory neurons
Granule neurons
Hepatocytes
Inhibitory interneurons
Inhibitory neuron progenitors
Inhibitory neurons
Intermediate Mesoderm
Isthmic organizer cells
Jaw and tooth progenitors
Lens
Limb mesenchyme
Megakaryocytes
Melanocytes
Myocytes
Neural progenitor cells
Neural Tube
Notochord cells
Oligodendrocyte Progenitors
Osteoblasts
Postmitotic premature neurons
Premature oligodendrocyte
Primitive erythroid lineage
Radial glia
Schwann cell precursor
Sensory neurons
Stromal cells
White blood cells
B
Mouse liver cancer
UMAP_1
UMAP_2
Tumor I
Tumor II
Tumor III
Interferon
Hepatocyte I
Hepatocyte II
Hepatic Stellate Cell
VSMC
Ribosomal+
LSEC
Kupffer/Monocyte
Monocyte
T
B
C
Mouse hippocampus
UMAP_1
UMAP_2
Astrocyte
CA1
CA3
Cajal_Retzius
Choroid
Denate
Endothelial_Stalk
Endothelial_Tip
Entorihinal
Ependymal
Interneuron
Microglia_Macrophages
Mural
Neurogenesis
Neuron.Slc17a6
Oligodendrocyte
Polydendrocyte
NA

**Appendix Figure S8. UMAP projections for single cell/nucleus data.**

A UMAP of scRNA-seq data from a mouse embryo at E12.5. It consists of 37 cell types.

B UMAP of snRNA-seq data from mouse liver cancer. It has 14 cell types.

C UMAP of scRNA-seq data from mouse hippocampus. It is composed of 17 cell types.

**A**

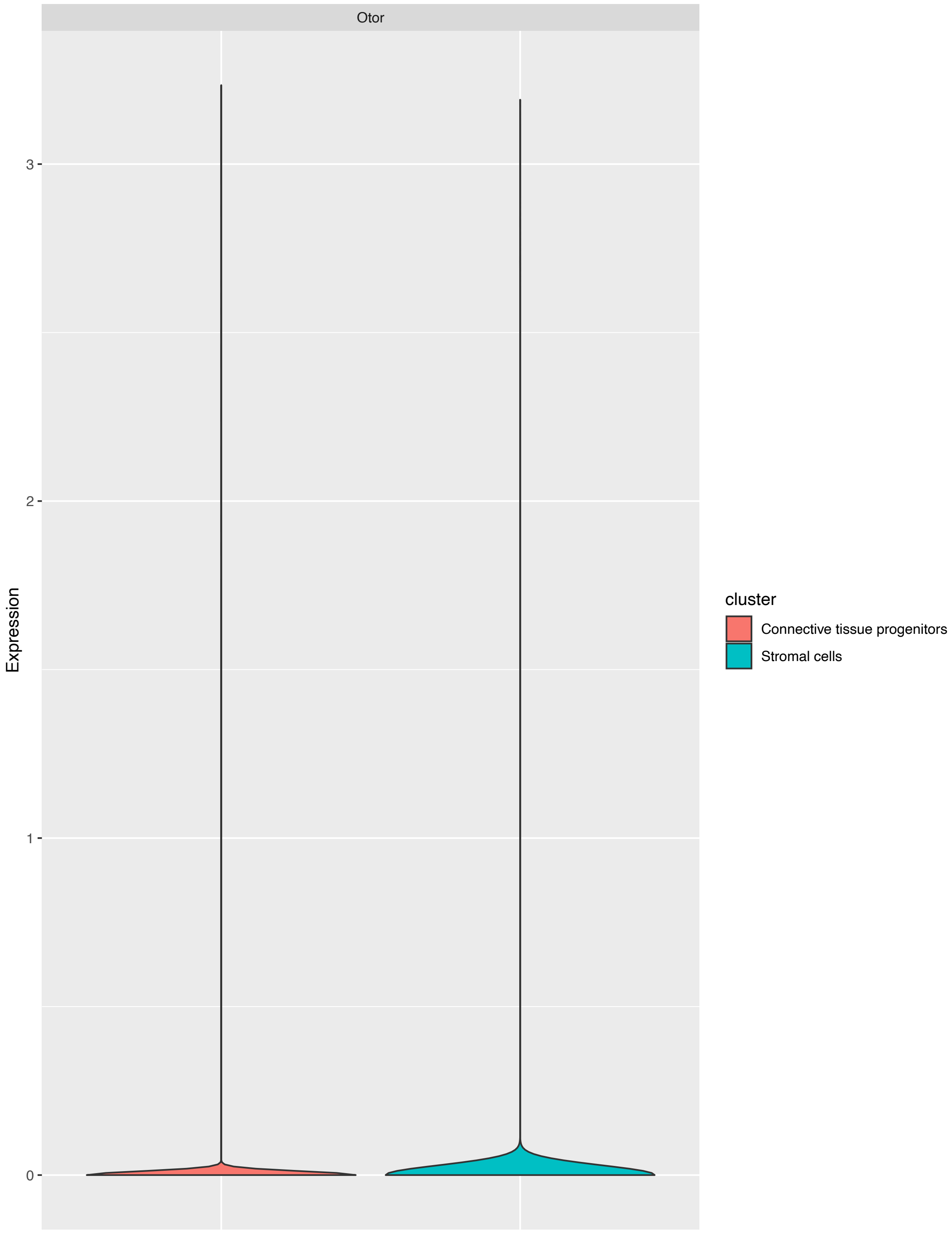

Otor
Expression
3
2
1
0
cluster
Connective tissue progenitors
Stromal cells

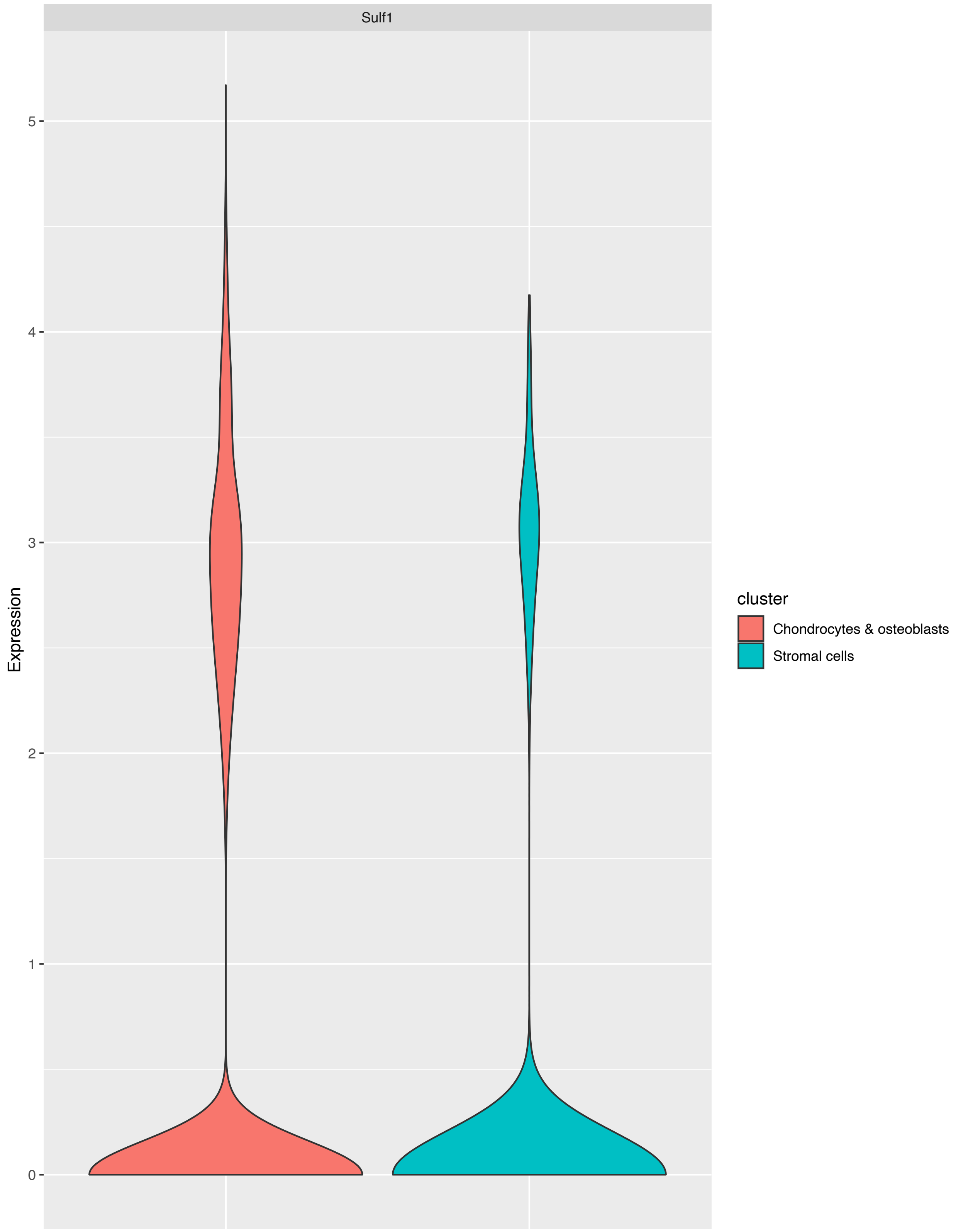
Sulf1
Expression
5
4
3
2
1
0
cluster
Chondrocytes & osteoblasts
Stromal cells

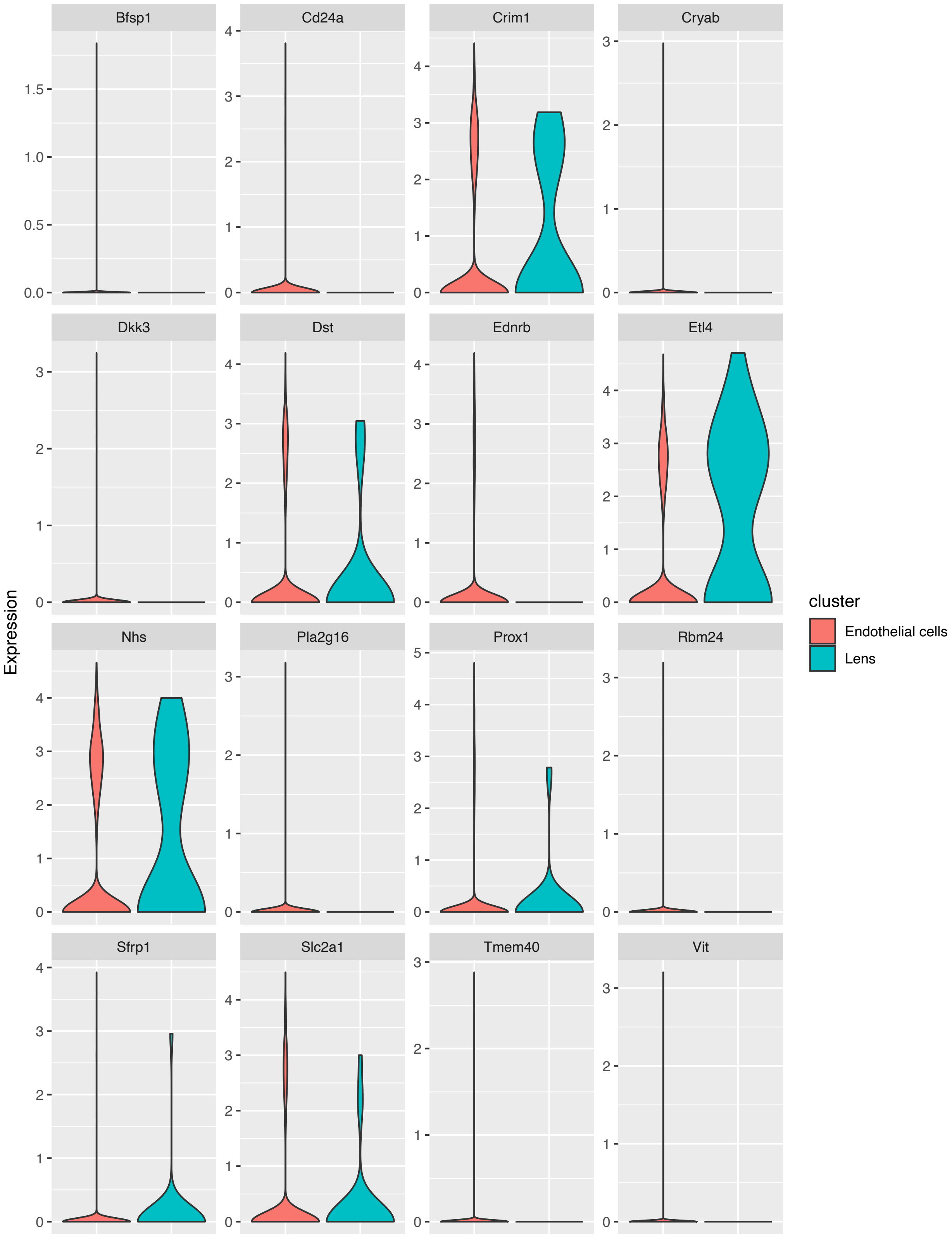

Bfsp1
Cd24a
Crim1
Cryab
Dkk3
Dst
Ednrb
Etl4
Nhs
Pla2g16
Prox1
Rbm24
Sfrp1
Slc2a1
Tmem40
Vit
Expression
cluster
Endothelial cells
Lens

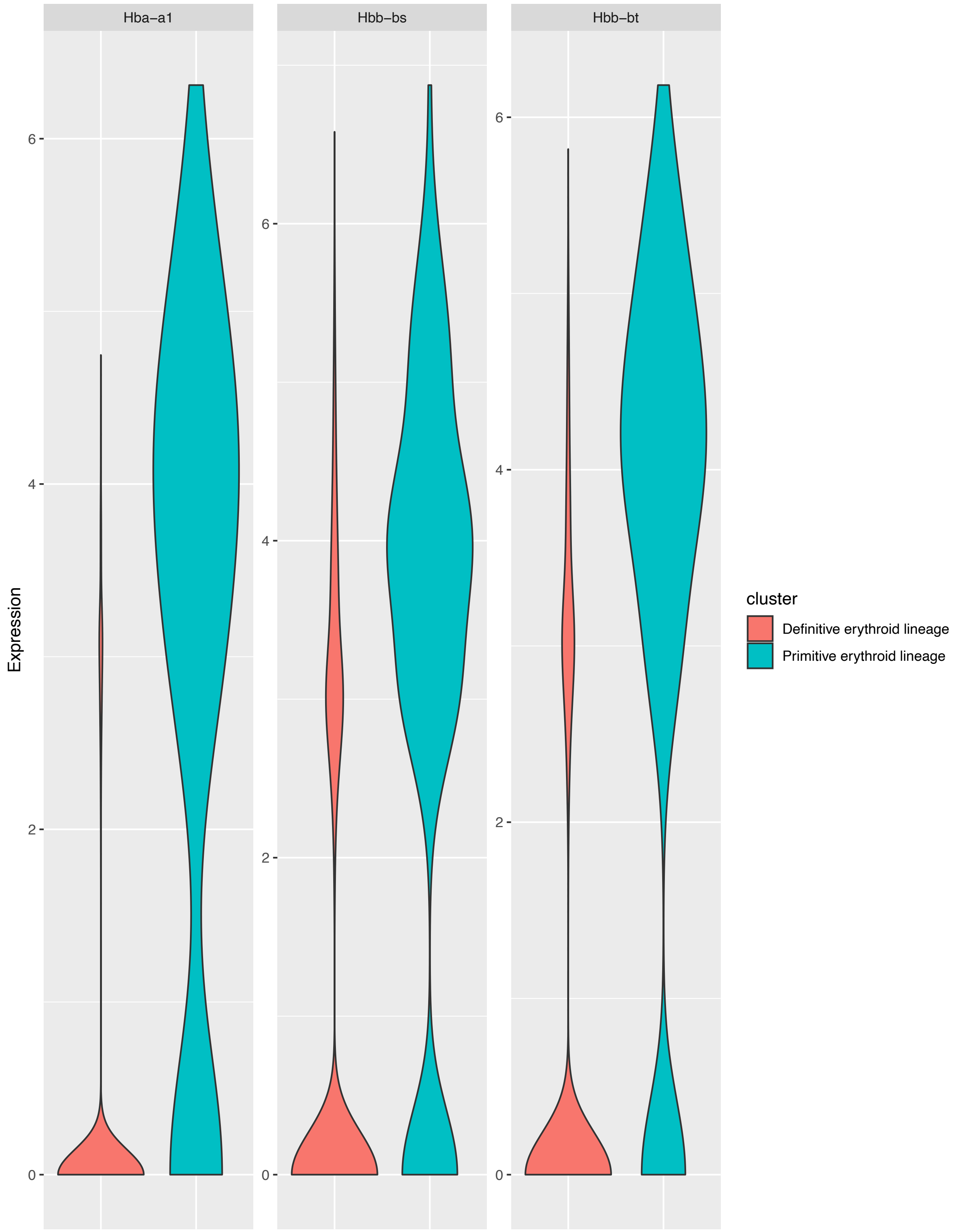
Hba−a1
Hbb−bs
Hbb−bt
Expression
0
2
4
6
cluster
Definitive erythroid lineage
Primitive erythroid lineage

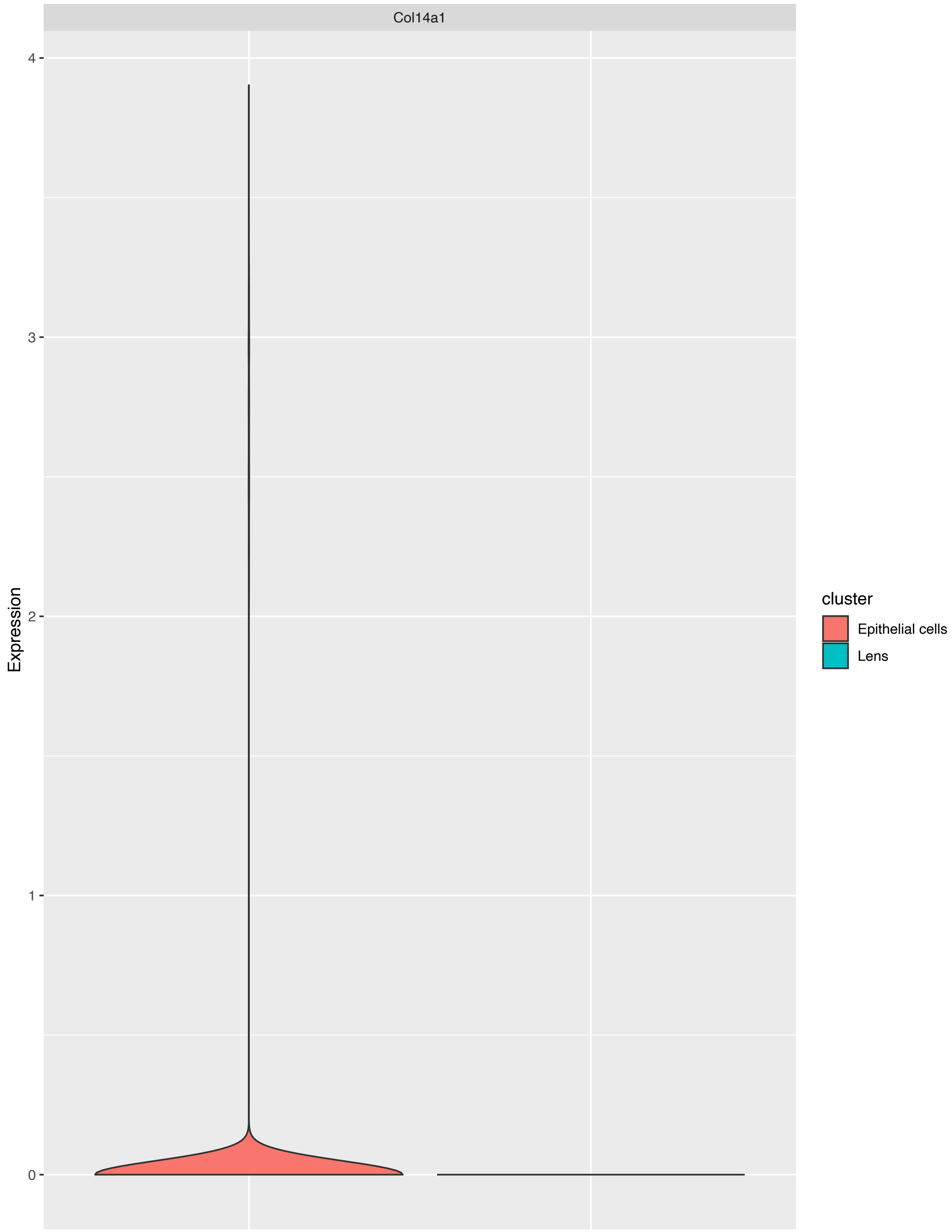

Col14a1
Expression
4
3
2
1
0
cluster
Epithelial cells
Lens

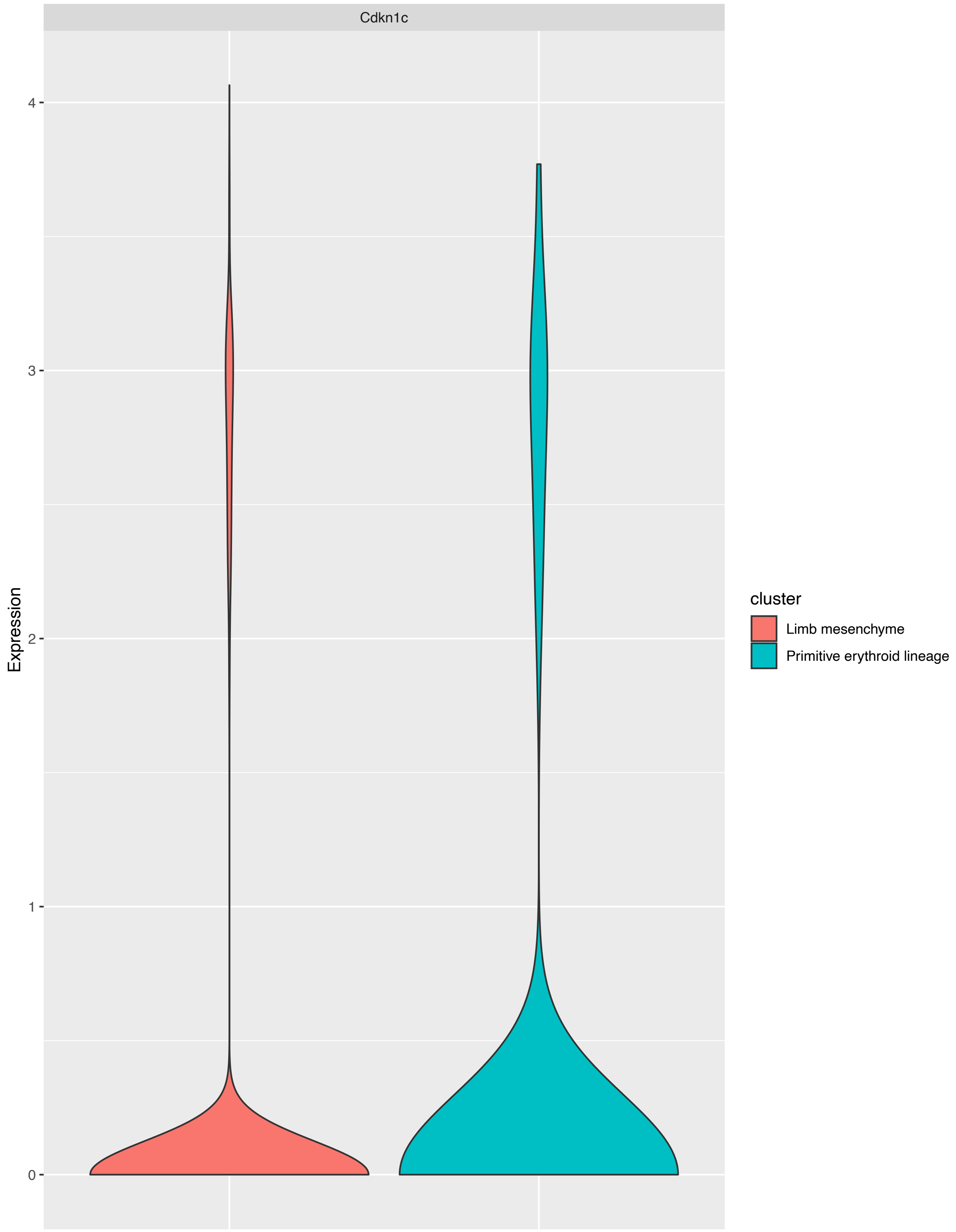

Cdkn1c
4
3
2
1
0
Expression
cluster
Limb mesenchyme
Primitive erythroid lineage

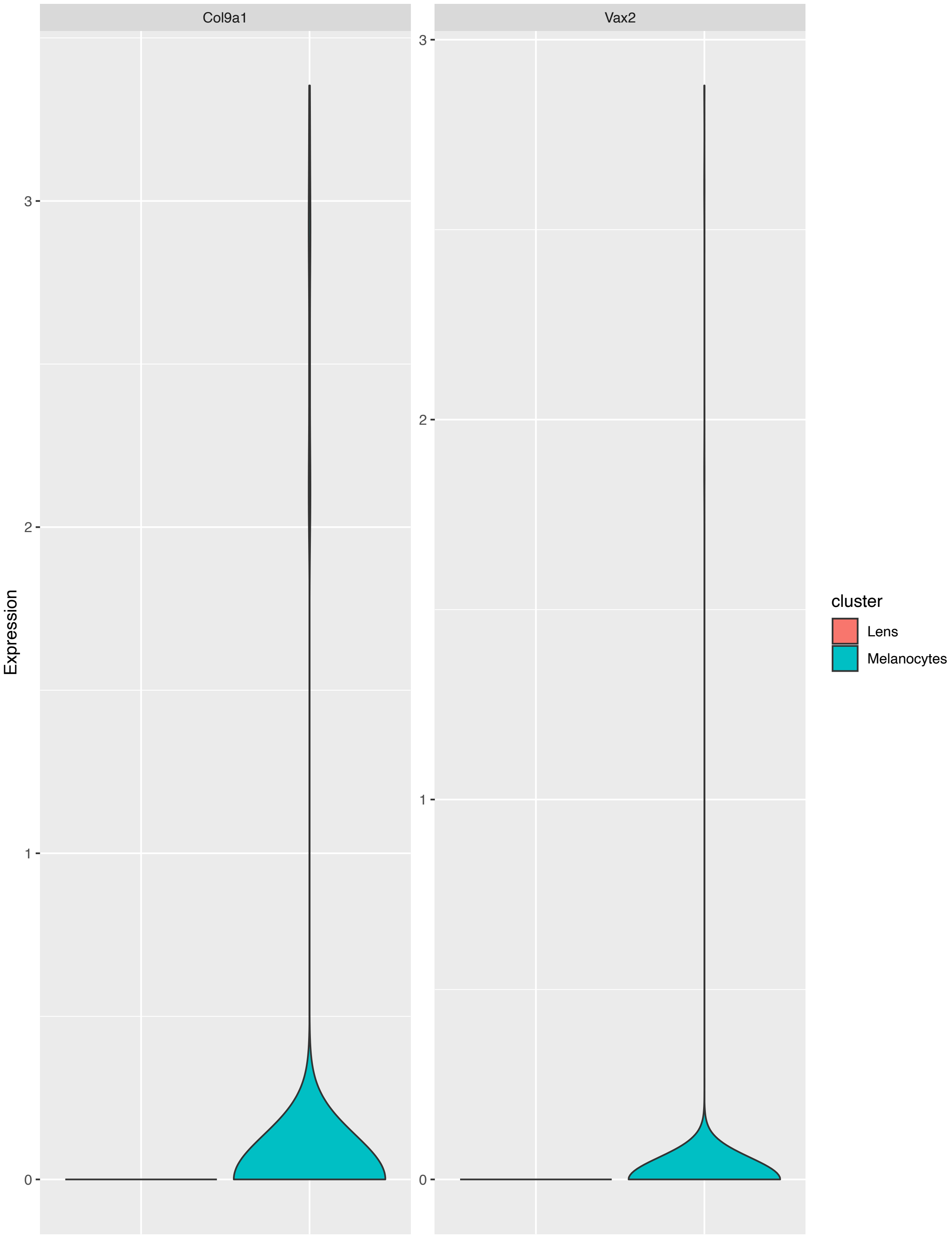
Col9a1
Vax2
Expression
3
2
1
0
cluster
Lens
Melanocytes

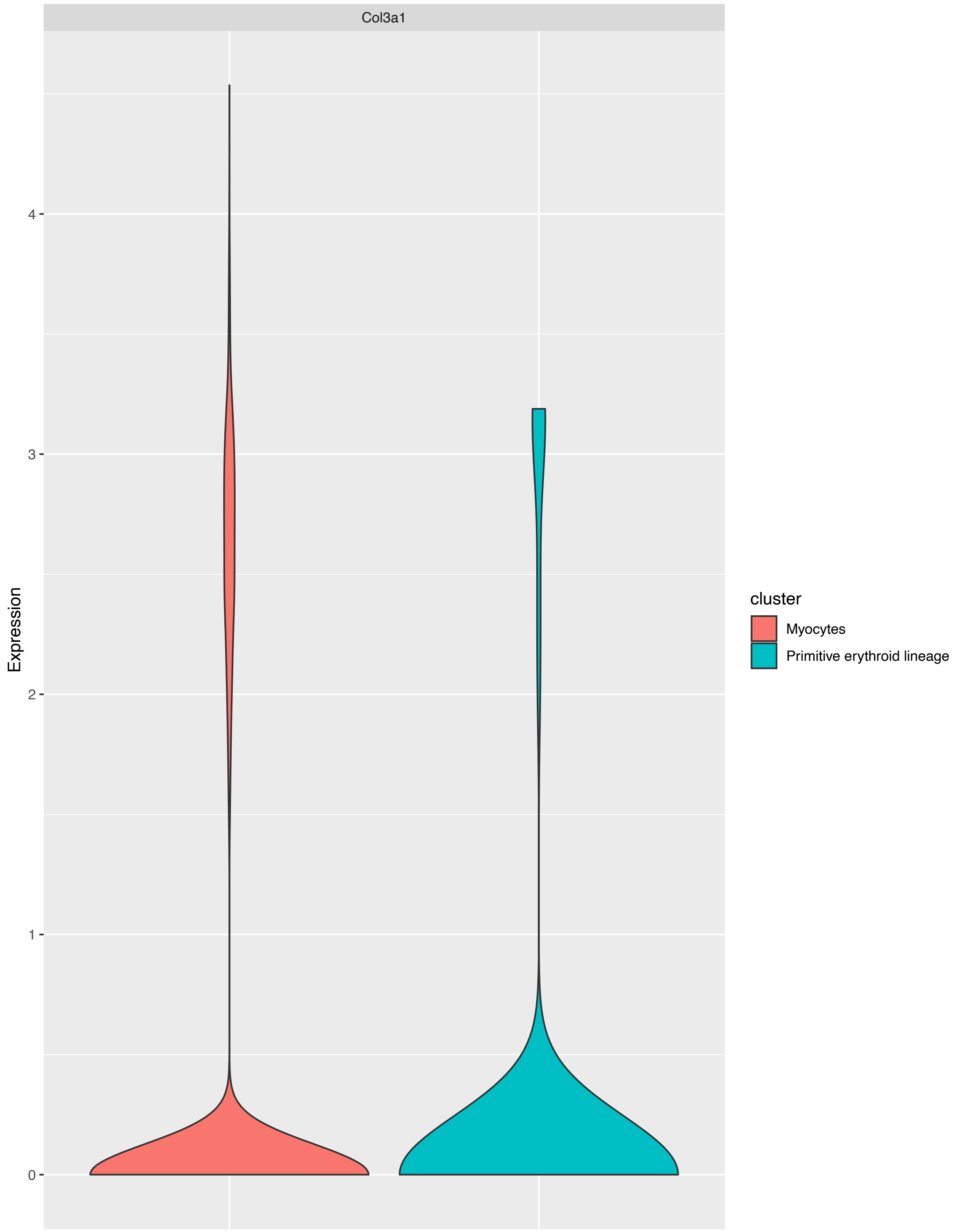
Col3a1
Expression
0
1
2
3
4
cluster
Myocytes
Primitive erythroid lineage

**B**

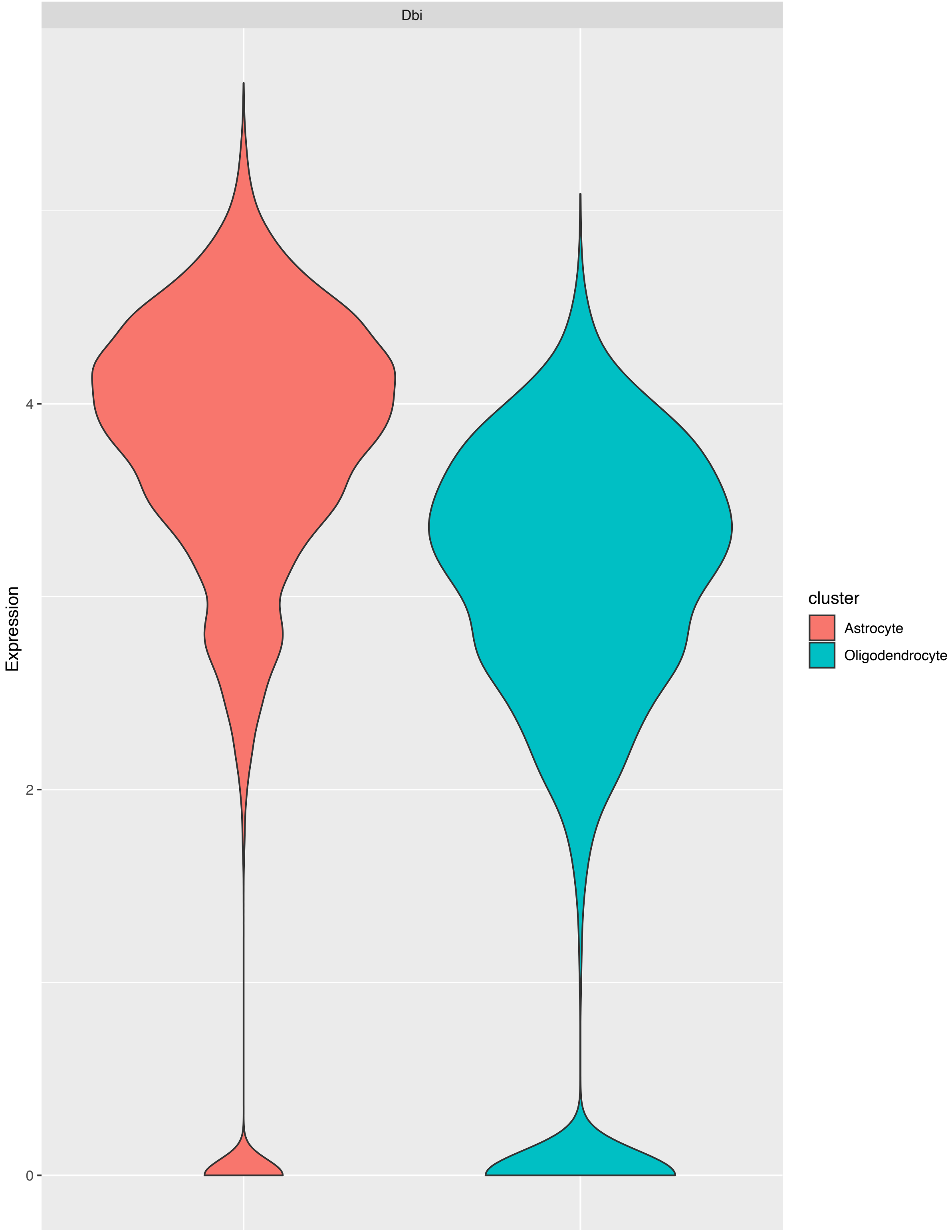

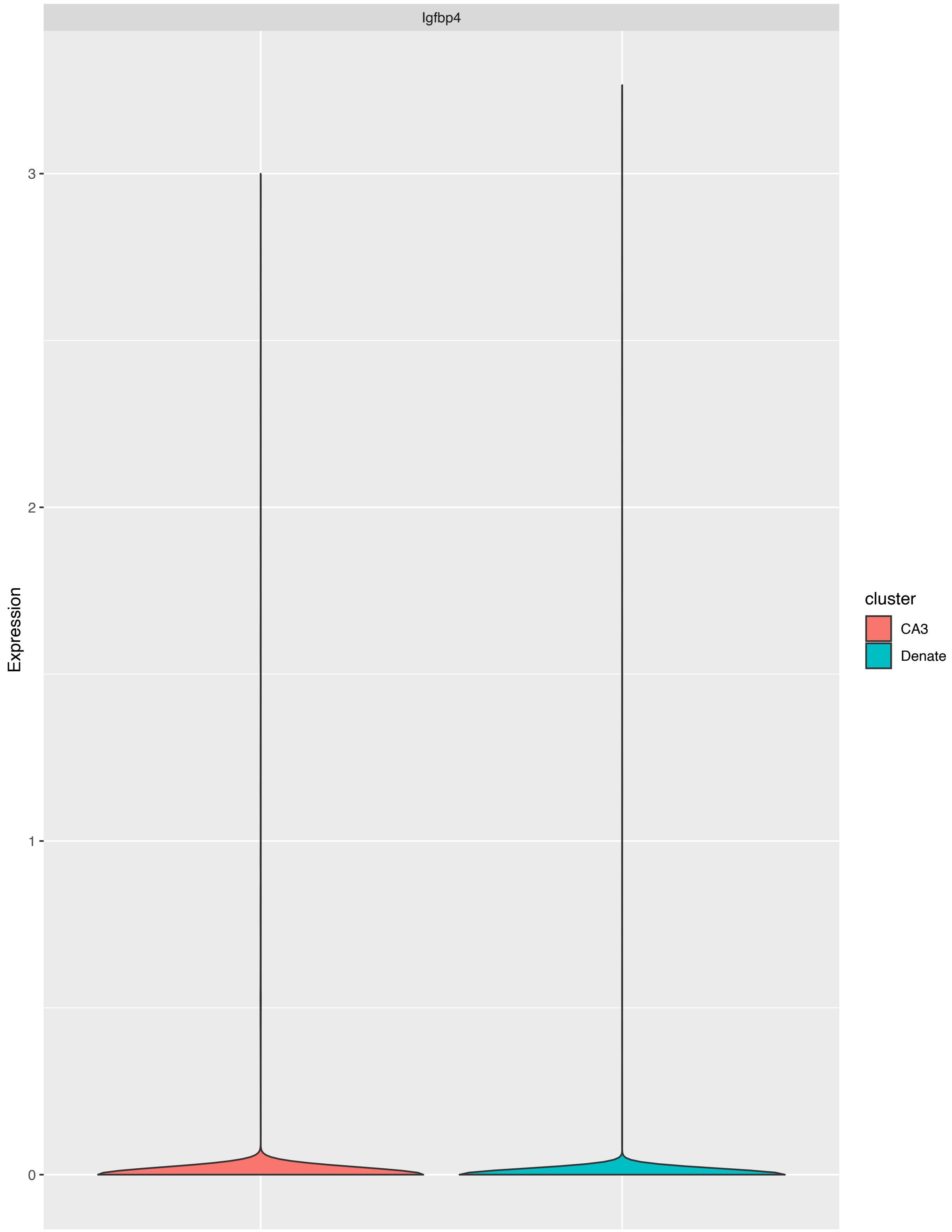
Igfbp4
3
2
1
0
Expression
cluster
CA3
Denate

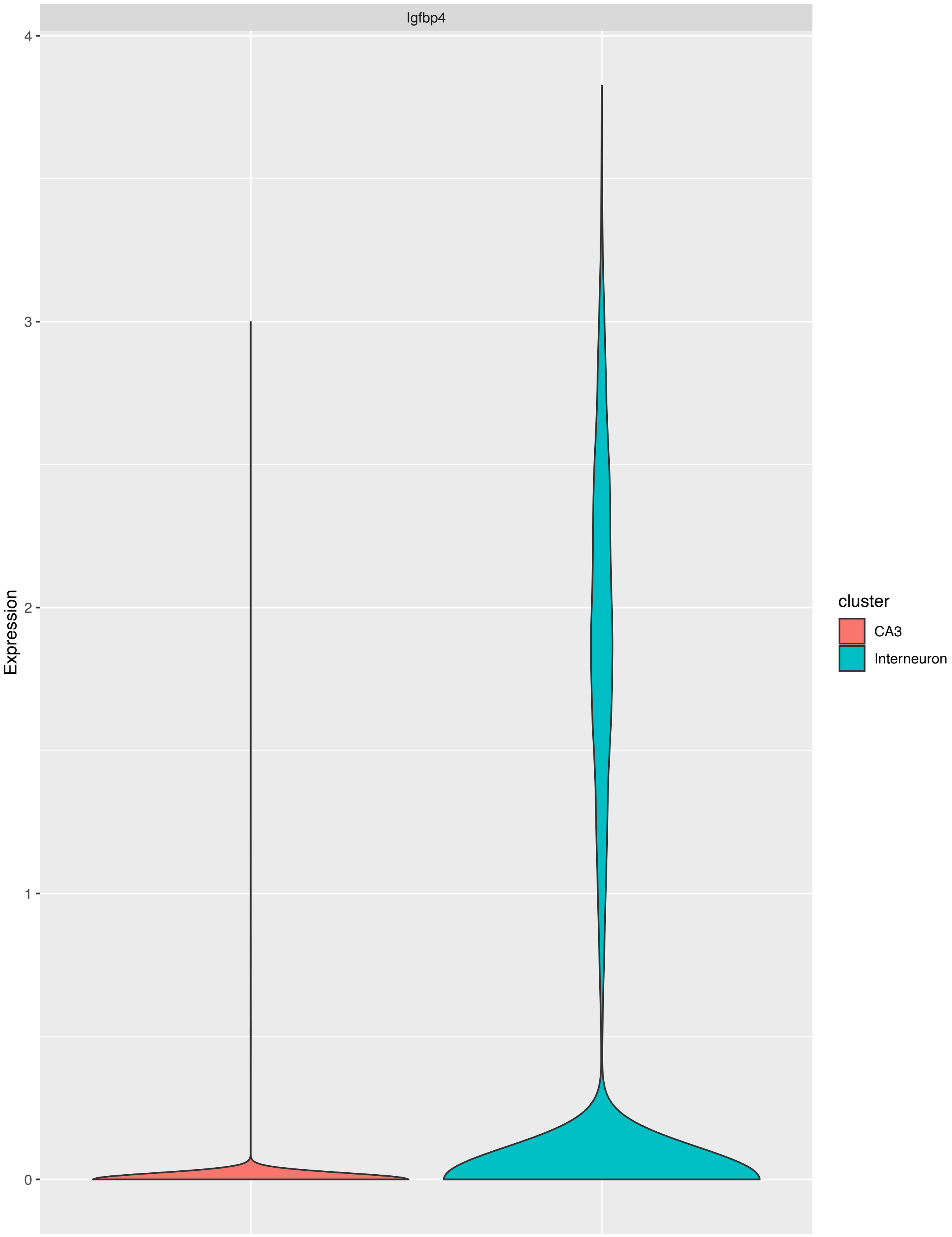
Igfbp4
4
3
2
1
0
Expression
cluster
CA3
Interneuron

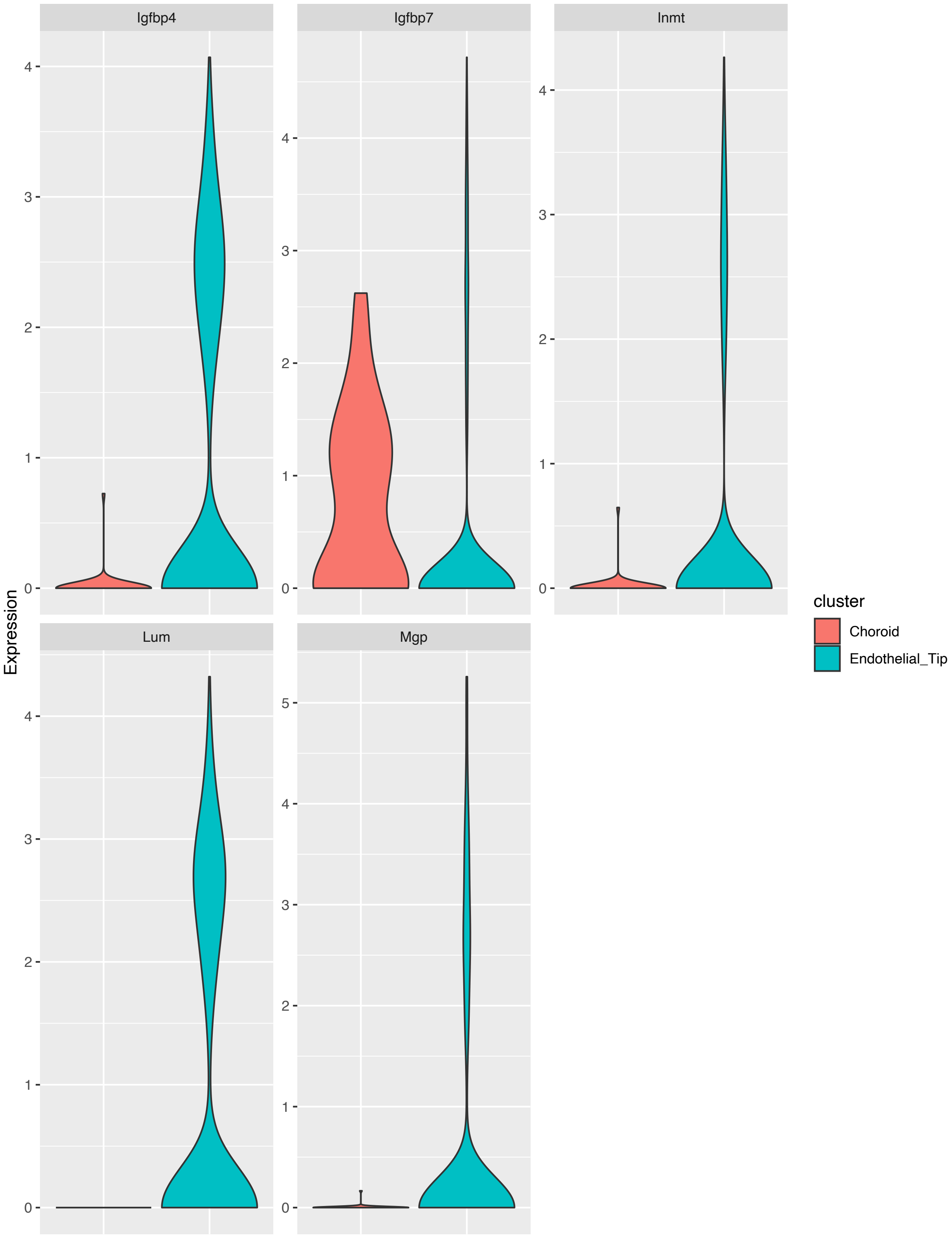
Igfbp4
Igfbp7
Inmt
Lum
Mgp
Expression
cluster
Choroid
Endothelial_Tip

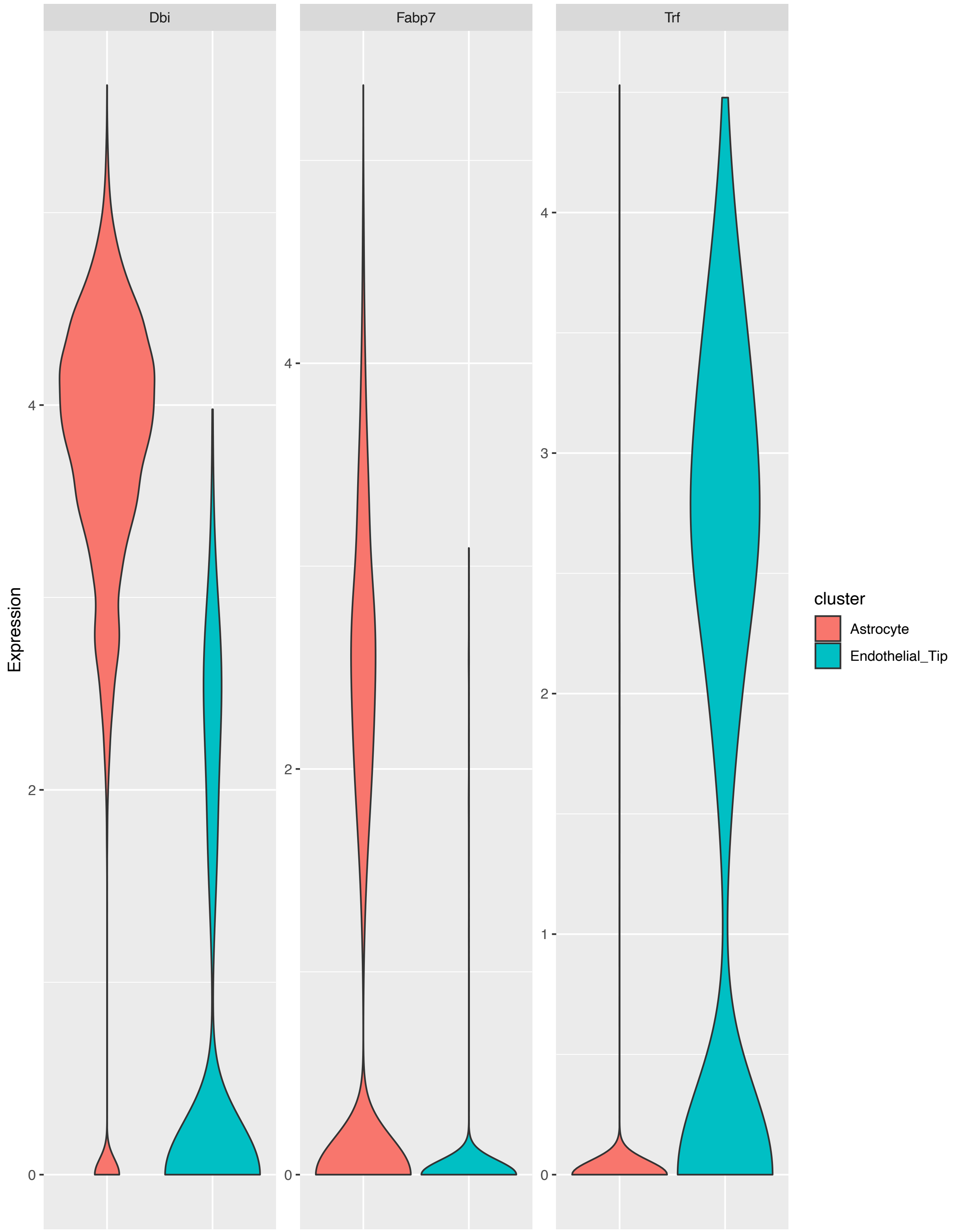
Dbi
Fabp7
Trf
Expression
0
2
4
0
2
4
0
1
2
3
4
cluster
Astrocyte
Endothelial_Tip

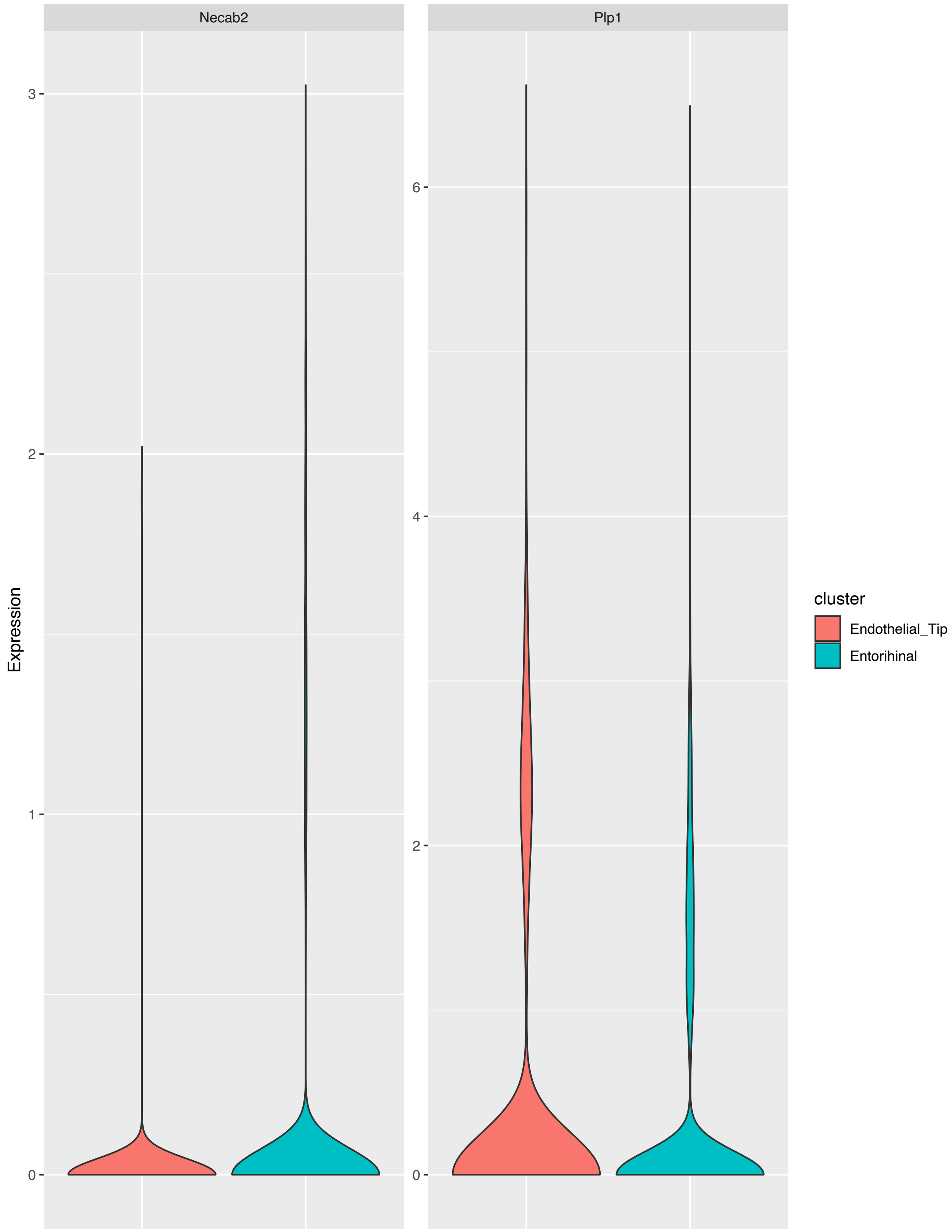
Necab2
Plp1
Expression
3
2
1
0
6
4
2
0
cluster
Endothelial_Tip
Entorihinal

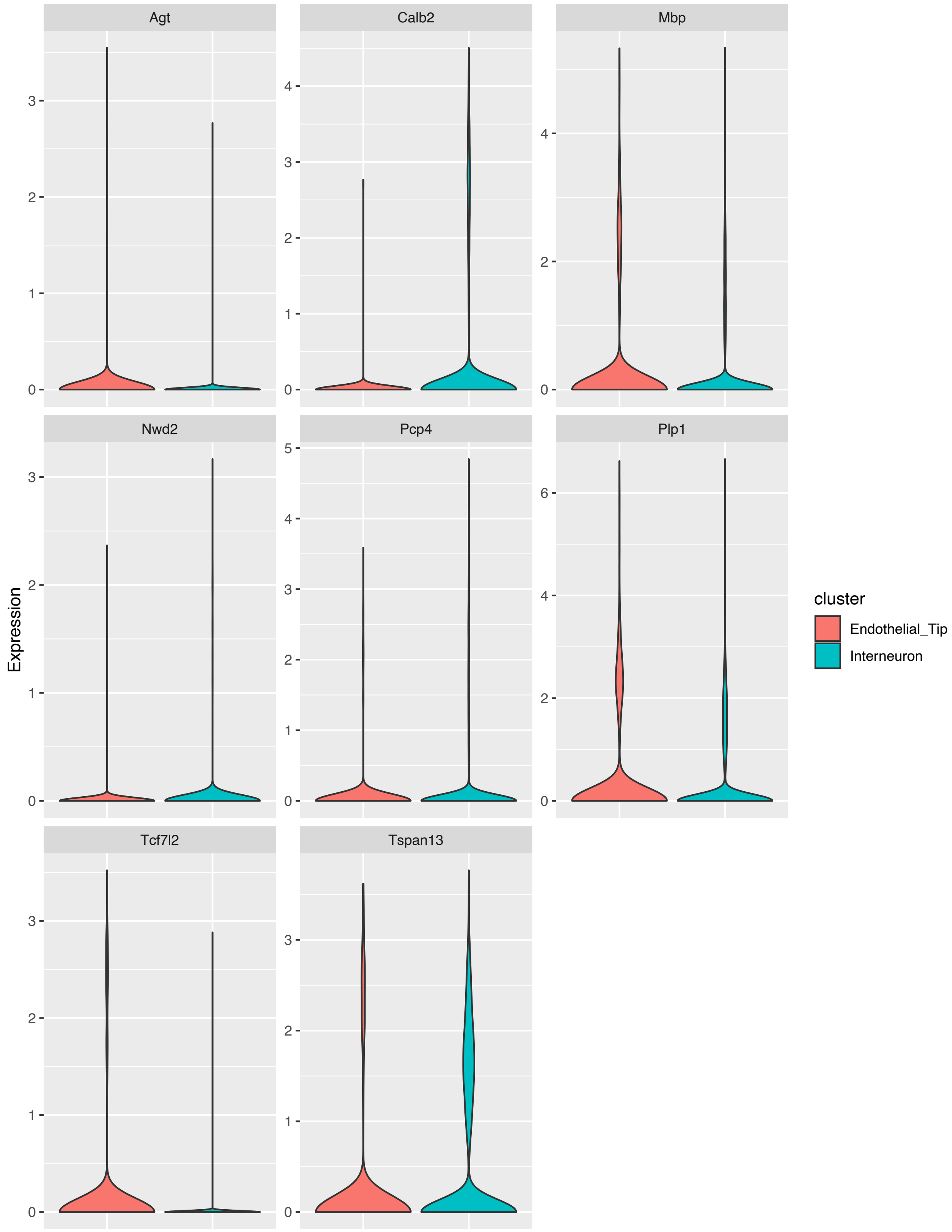
Agt
Calb2
Mbp
Nwd2
Pcp4
Plp1
Tcf7l2
Tspan13
Expression
cluster
Endothelial_Tip
Interneuron

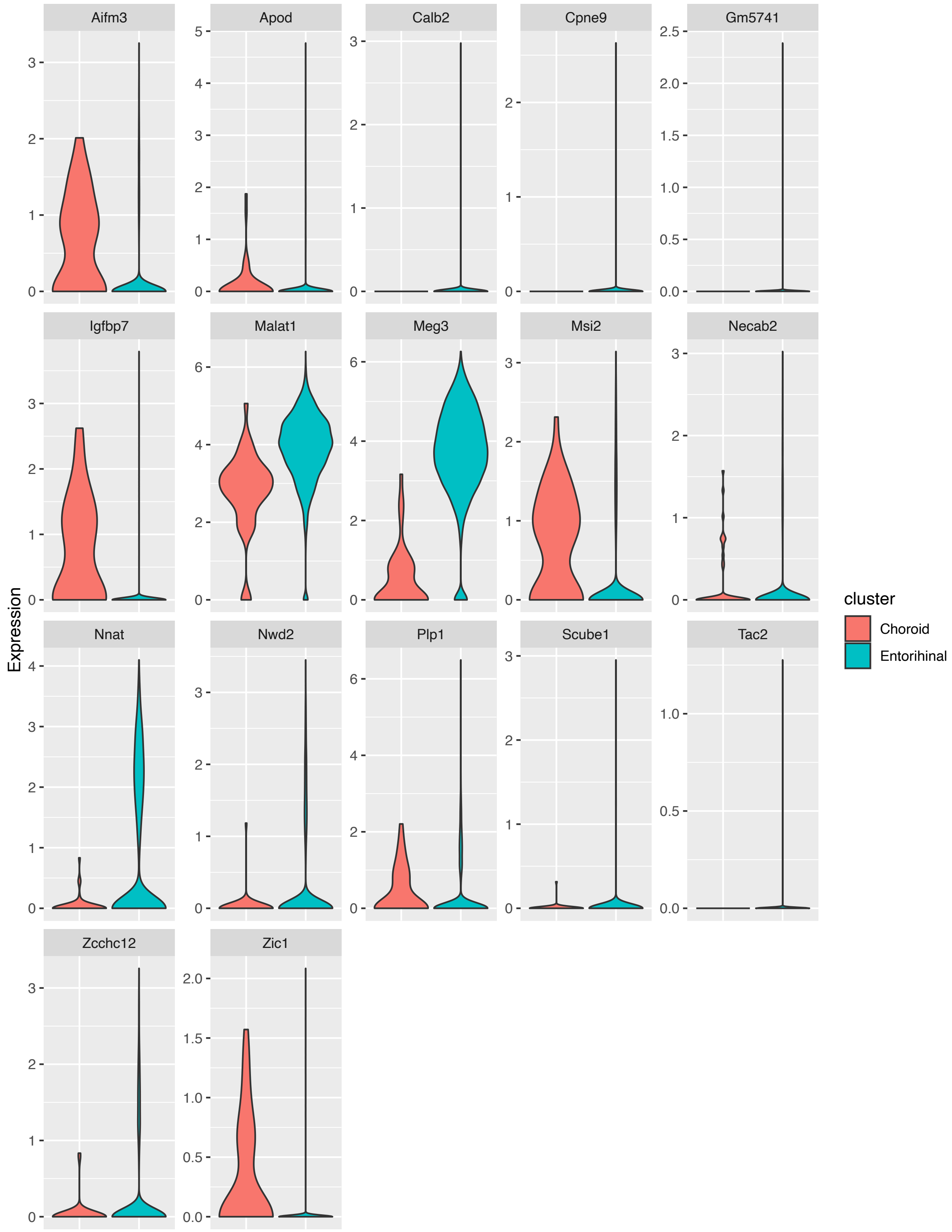
Aifm3
Apod
Calb2
Cpne9
Gm5741
Igfbp7
Malat1
Meg3
Msi2
Necab2
Nnat
Nwd2
Plp1
Scube1
Tac2
Zcchc12
Zic1
Expression
cluster
Choroid
Entorihinal

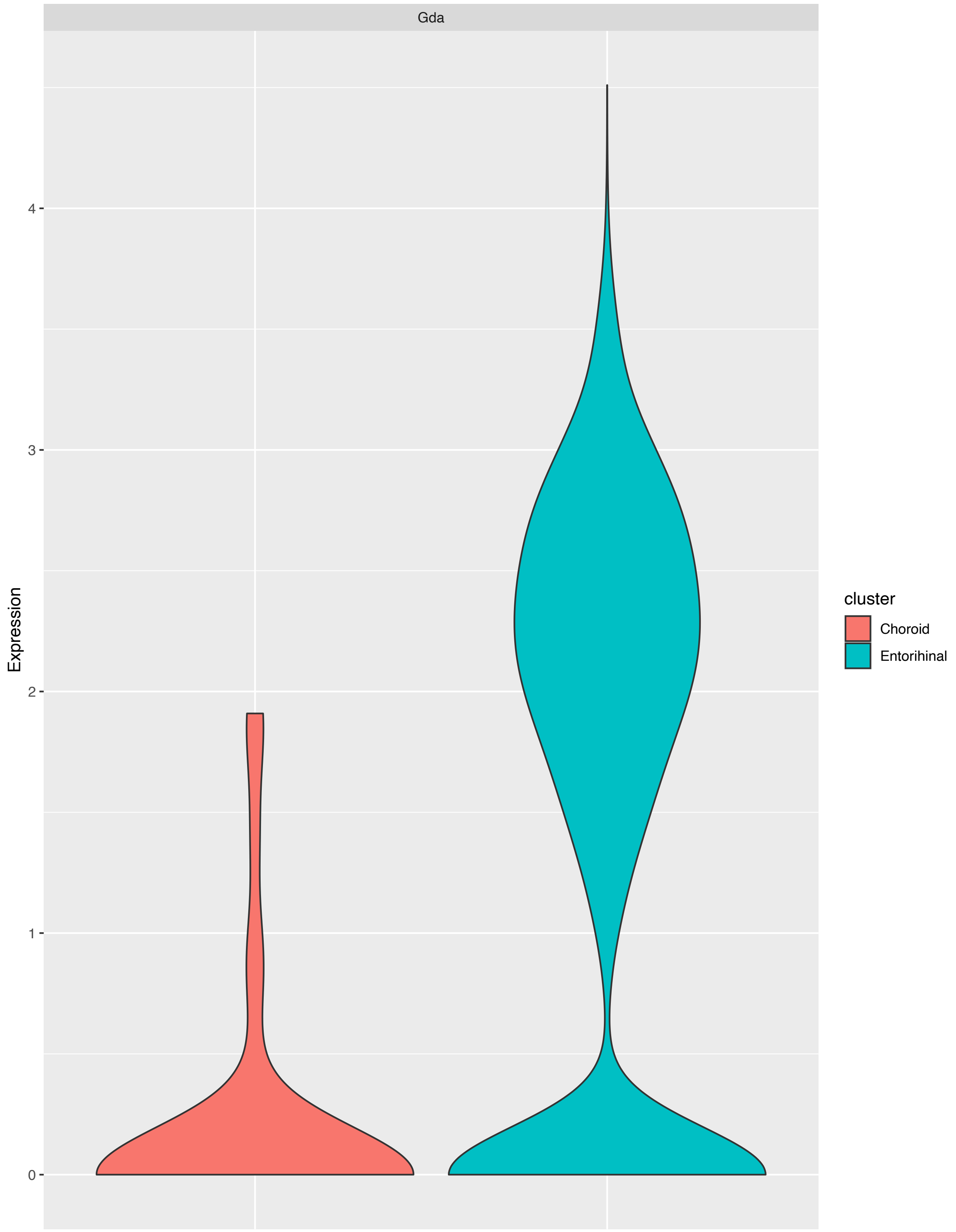
Gda
Expression
0
1
2
3
4
cluster
Choroid
Entorihinal

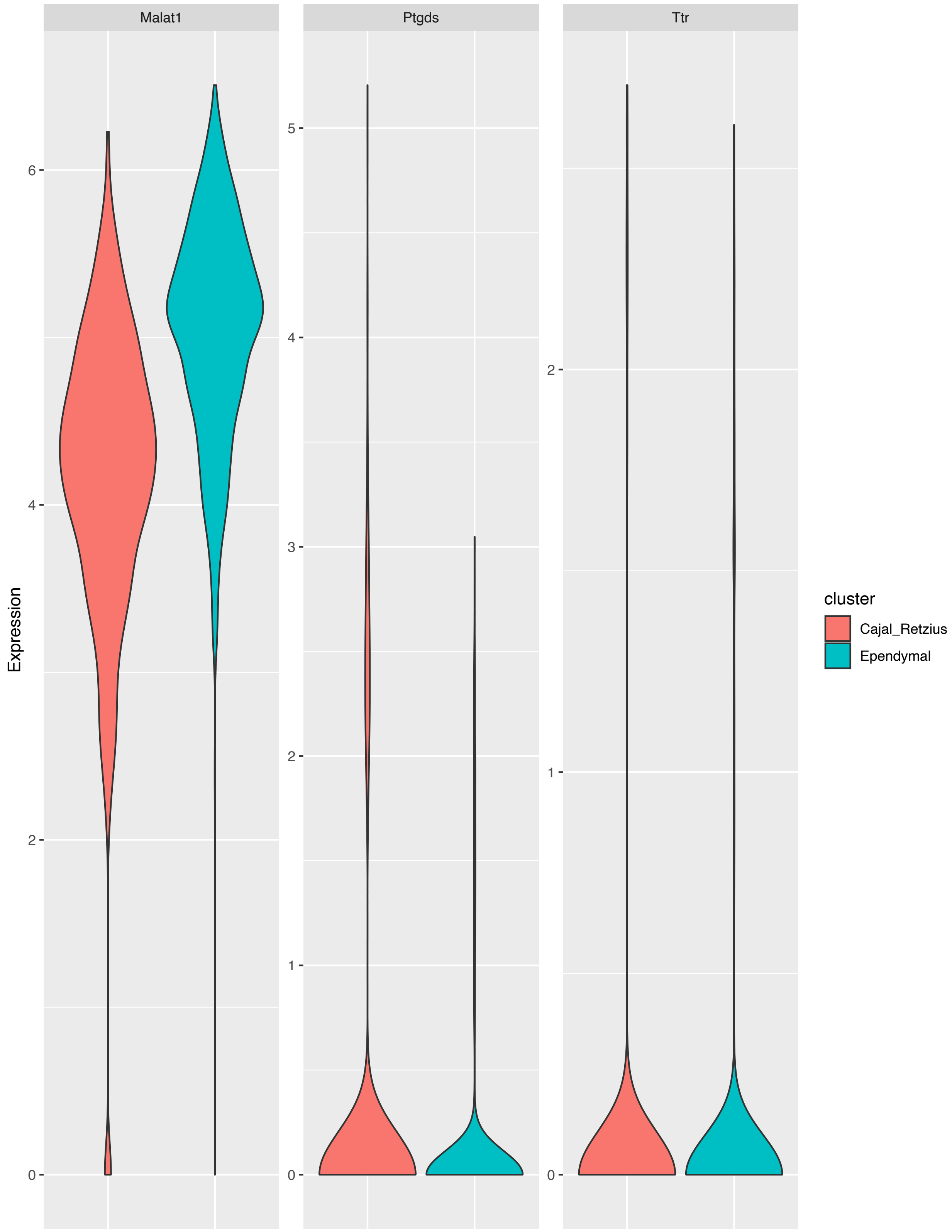
Malat1
Ptgds
Ttr
Expression
0
2
4
6
0
1
2
3
4
5
0
1
2
cluster
Cajal_Retzius
Ependymal

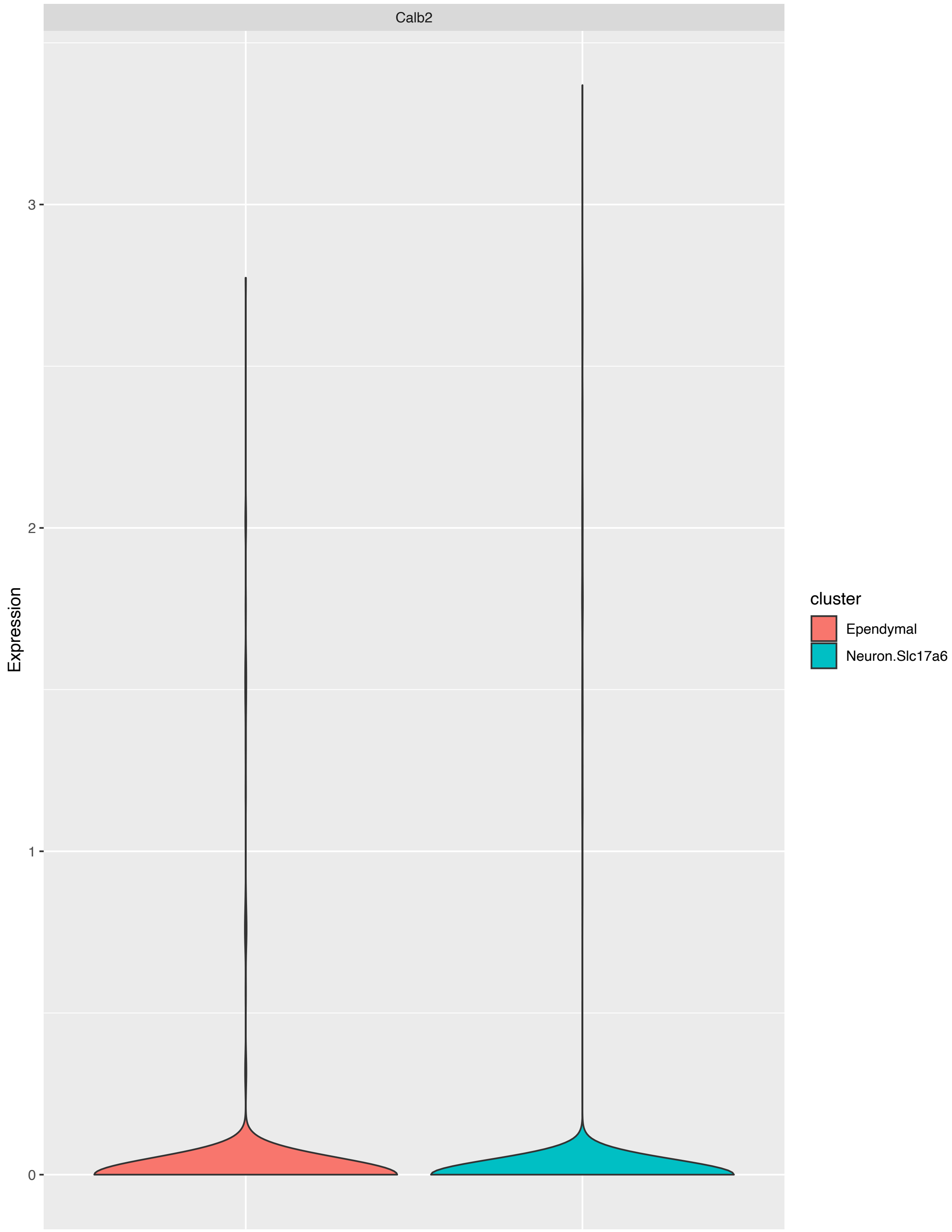

Calb2
Expression
3
2
1
0
cluster
Ependymal
Neuron.Slc17a6

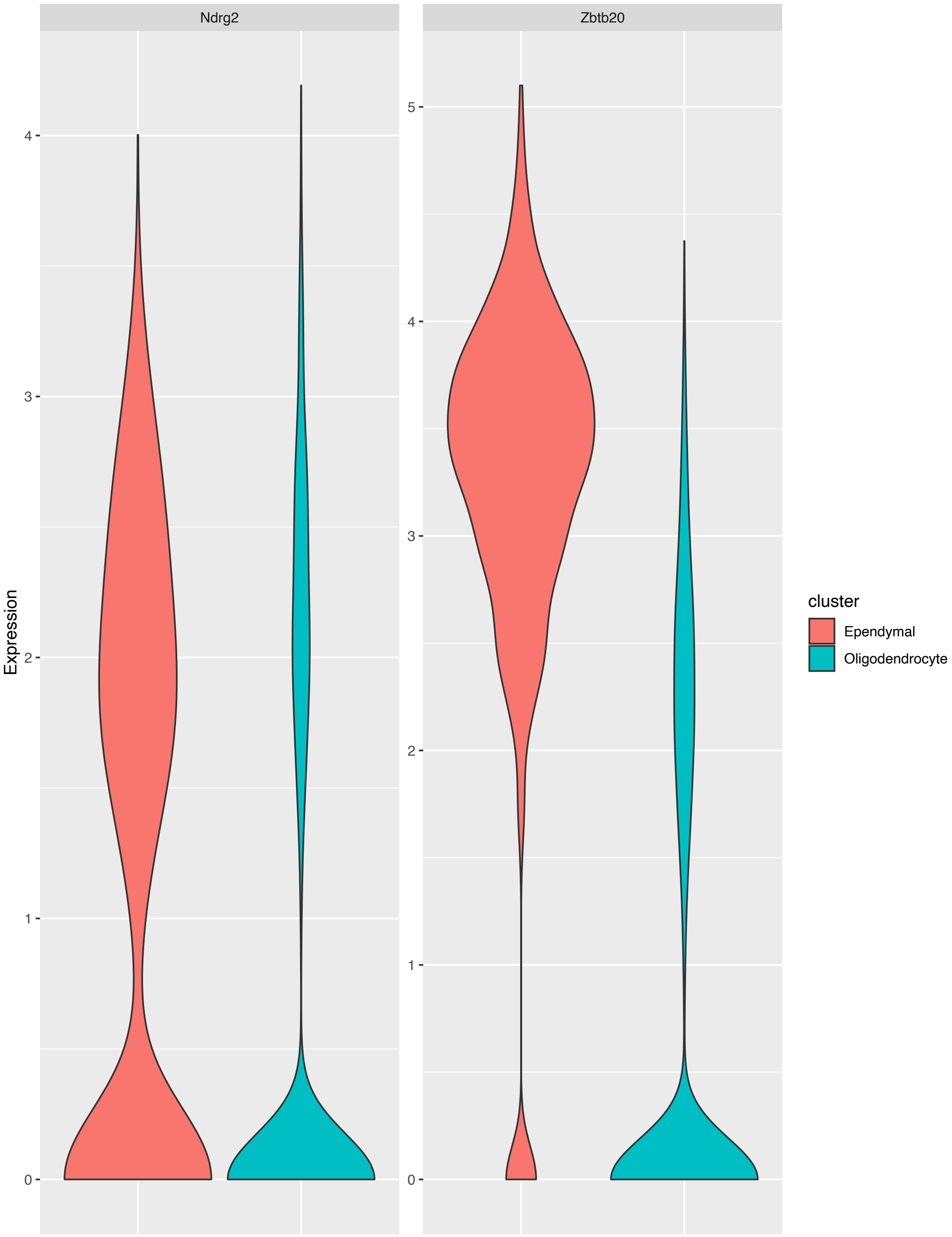
Ndrg2
Zbtb20
Expression
0
1
2
3
4
5
cluster
Ependymal
Oligodendrocyte

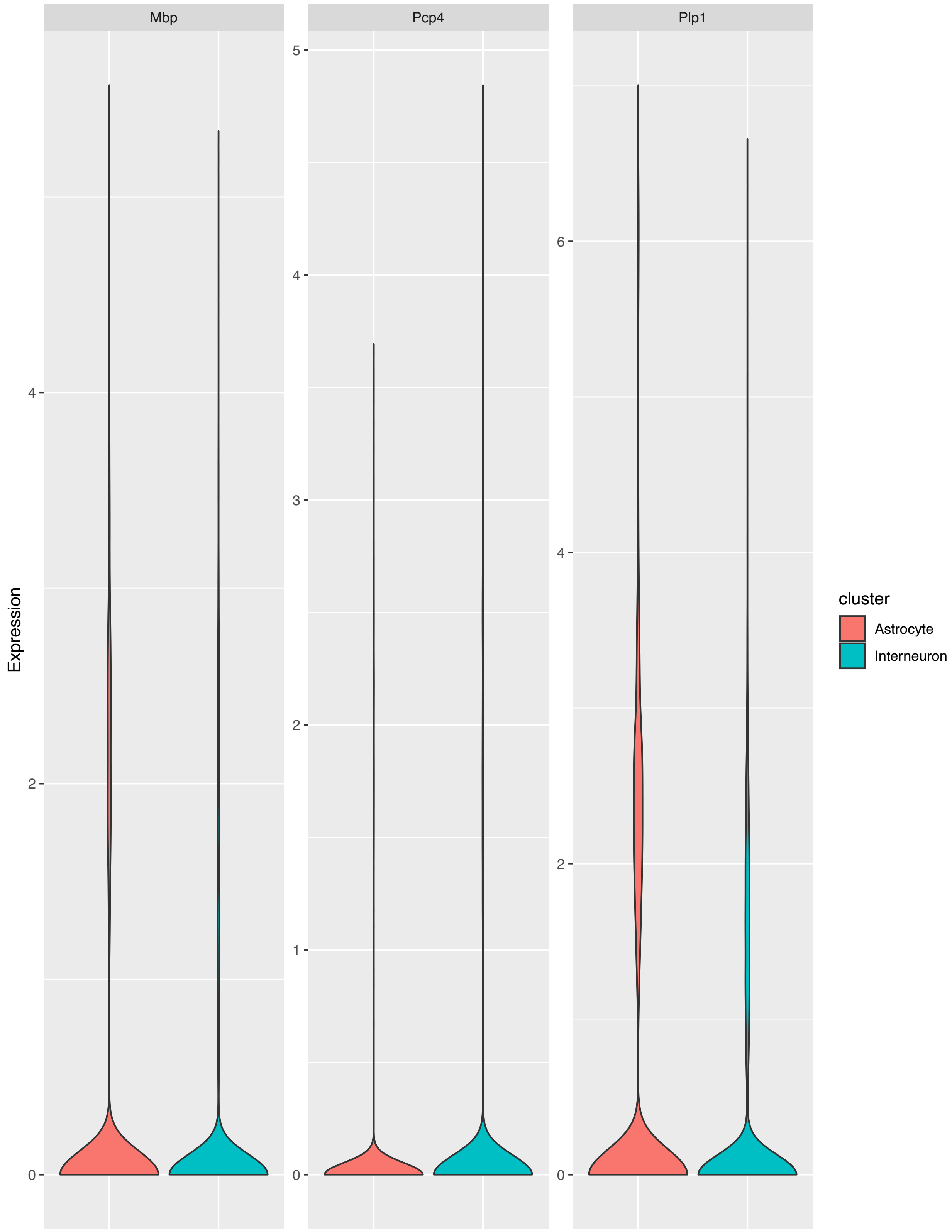

Mbp
Pcp4
Plp1
Expression
0
2
4
0
1
2
3
4
5
0
2
4
6
cluster
Astrocyte
Interneuron

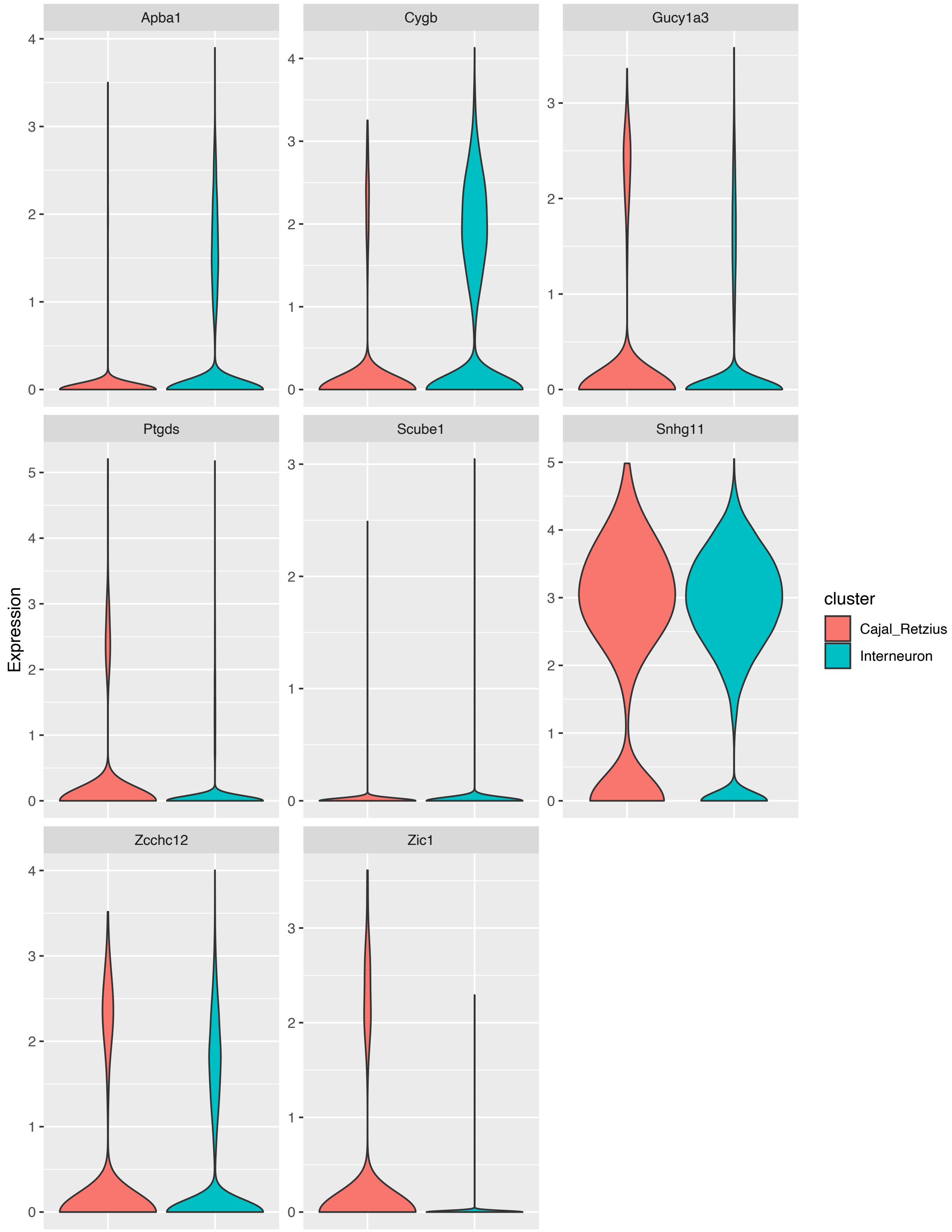
Apba1
Cygb
Gucy1a3
Ptgds
Scube1
Snhg11
Zcchc12
Zic1
Expression
cluster
Cajal_Retzius
Interneuron

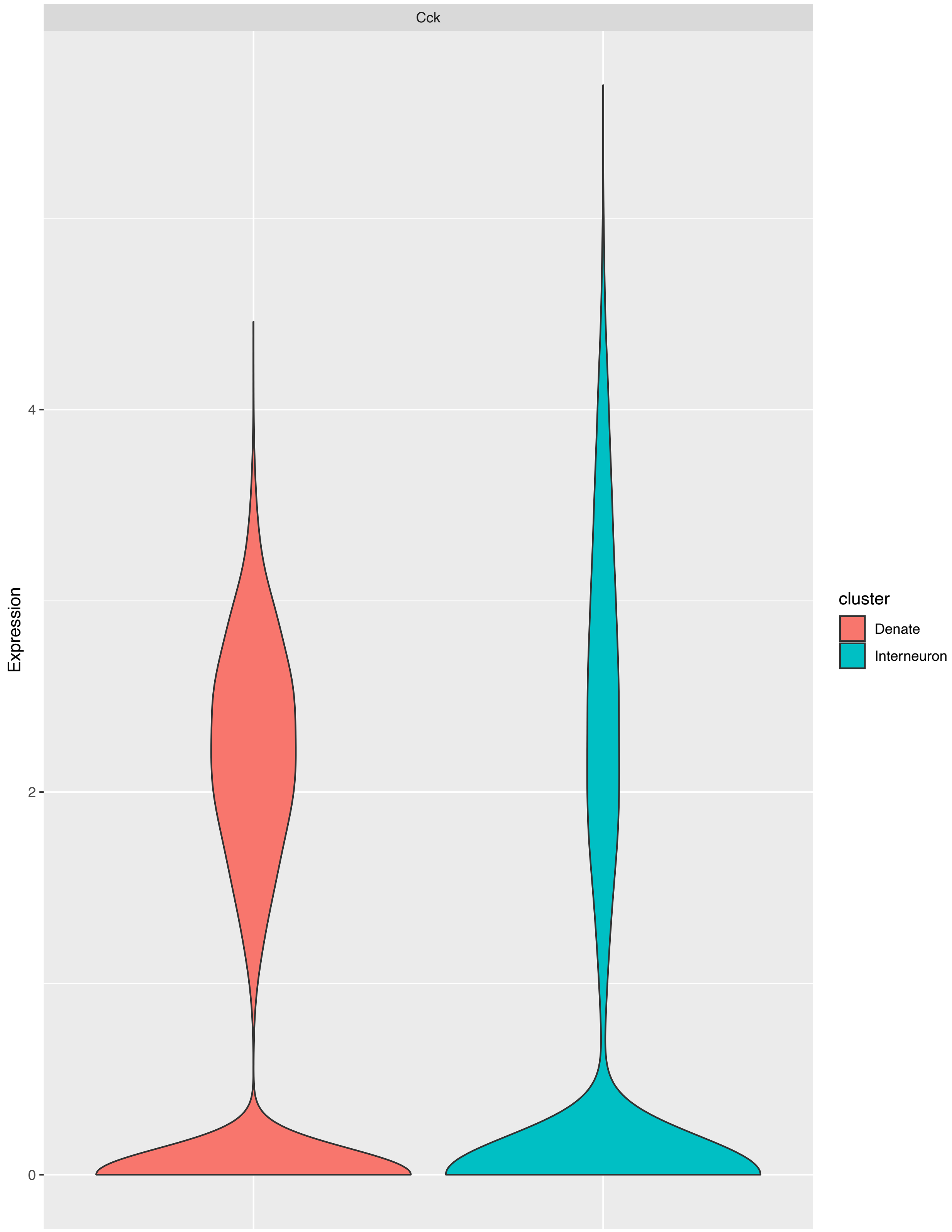
Cck
Expression
4
2
0
cluster
Denate
Interneuron

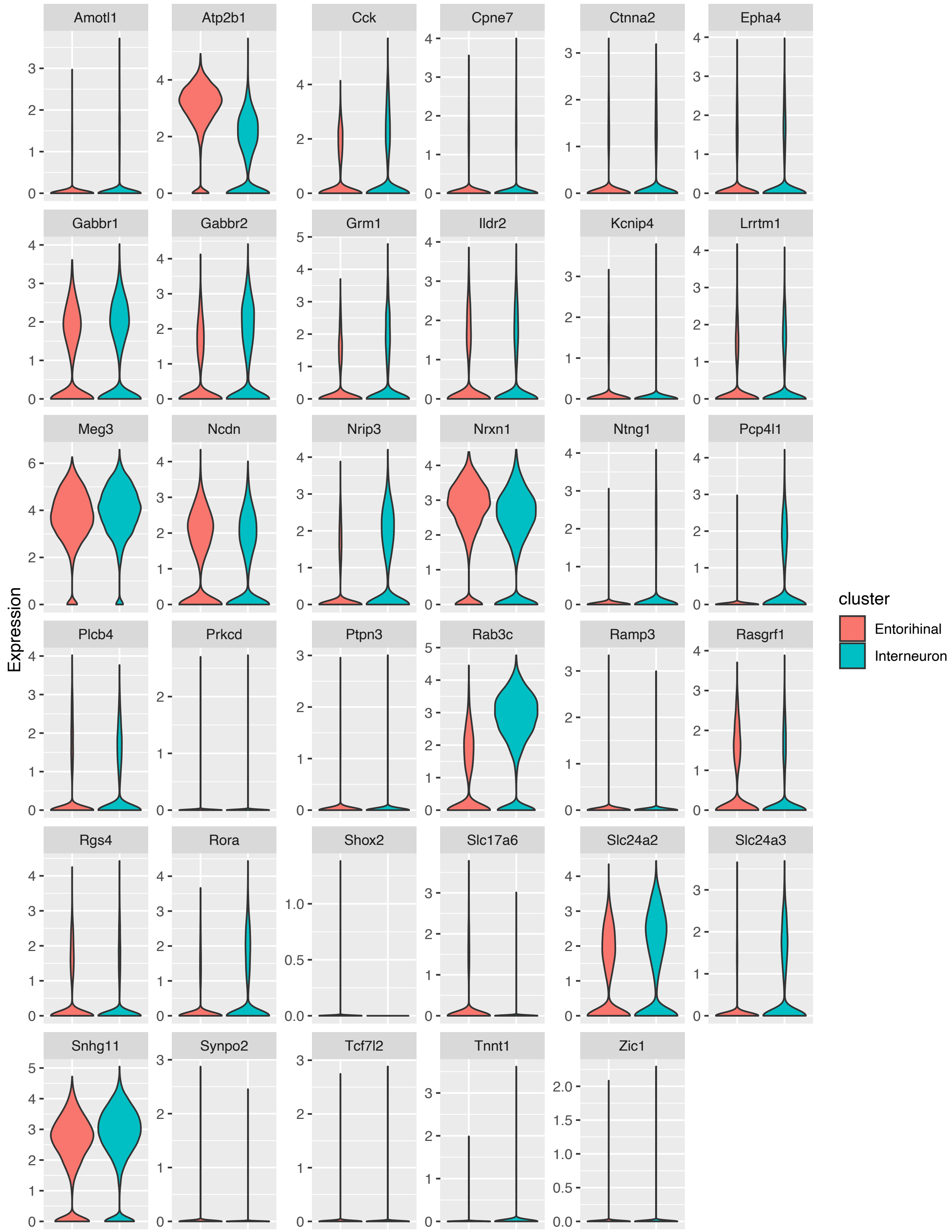

Amotl1
Atp2b1
Cck
Cpne7
Ctnna2
Epha4
Gabbr1
Gabbr2
Grm1
Ildr2
Kcnip4
Lrrtm1
Meg3
Ncdn
Nrip3
Nrxn1
Ntng1
Pcp4l1
Plcb4
Prkcd
Ptpn3
Rab3c
Ramp3
Rasgrf1
Rgs4
Rora
Shox2
Slc17a6
Slc24a2
Slc24a3
Snhg11
Synpo2
Tcf7l2
Tnnt1
Zic1
Expression
cluster
Entorihinal
Interneuron

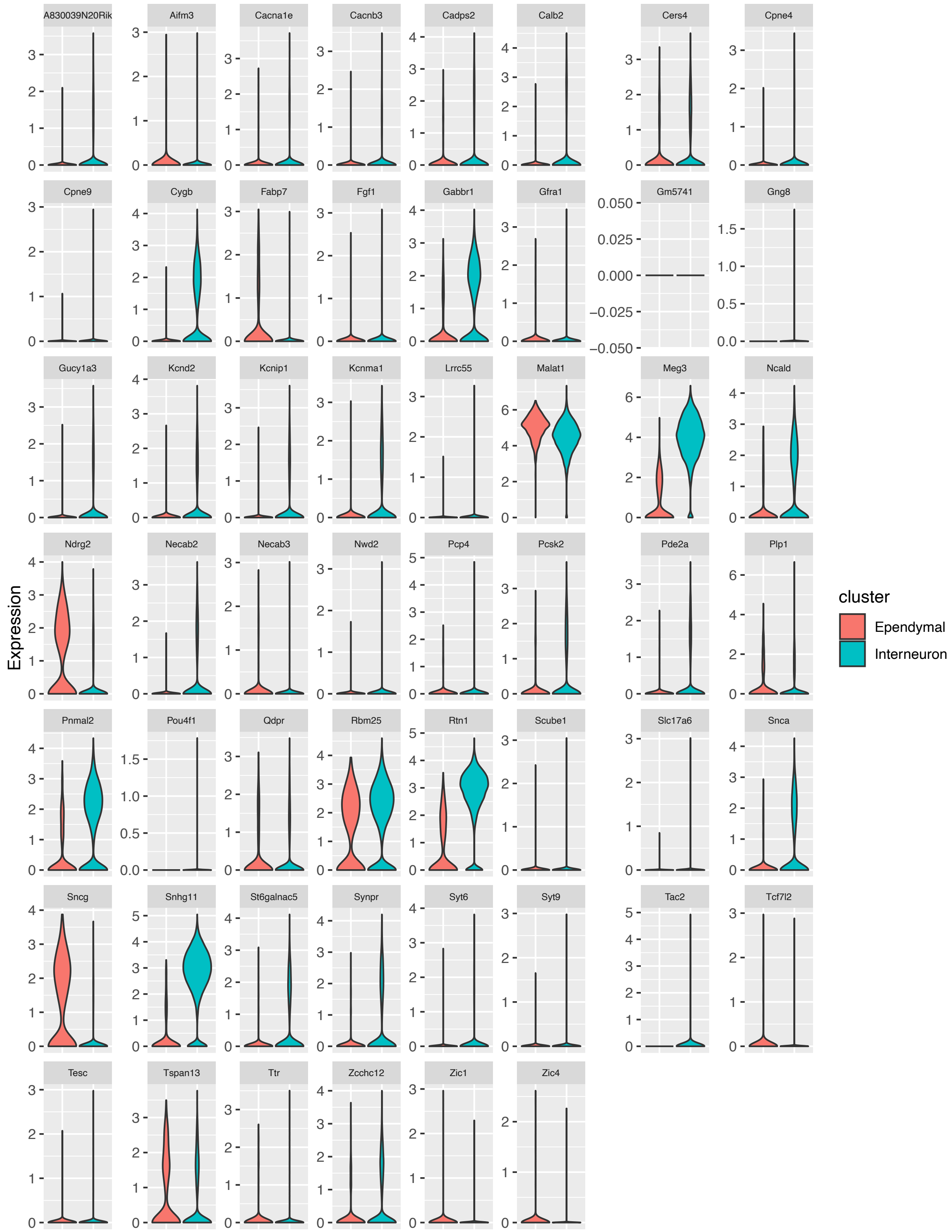
A830039N20Rik
Aifm3
Cacna1e
Cacnb3
Cadps2
Calb2
Cers4
Cpne4
Cpne9
Cygb
Fabp7
Fgf1
Gabbr1
Gfra1
Gm5741
Gng8
Gucy1a3
Kcnd2
Kcnip1
Kcnma1
Lrrc55
Malat1
Meg3
Ncald
Ndrg2
Necab2
Necab3
Nwd2
Pcp4
Pcsk2
Pde2a
Plp1
Pnmal2
Pou4f1
Qdpr
Rbm25
Rtn1
Scube1
Slc17a6
Snca
Sncg
Snhg11
St6galnac5
Synpr
Syt6
Syt9
Tac2
Tcf7l2
Tesc
Tspan13
Ttr
Zcchc12
Zic1
Zic4
Expression
cluster
Ependymal
Interneuron

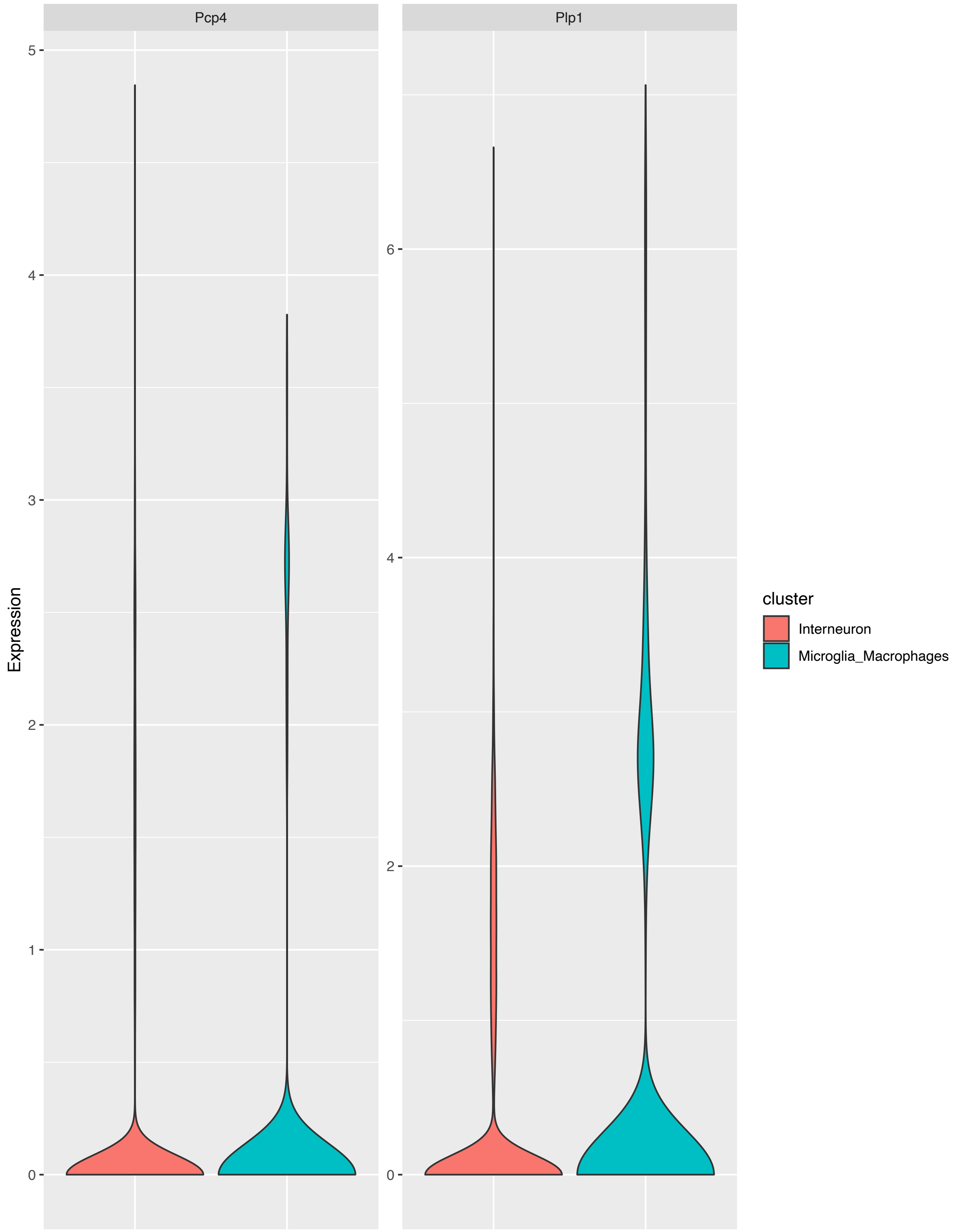

Pcp4
Plp1
Expression
5
4
3
2
1
0
6
4
2
0
cluster
Interneuron
Microglia_Macrophages

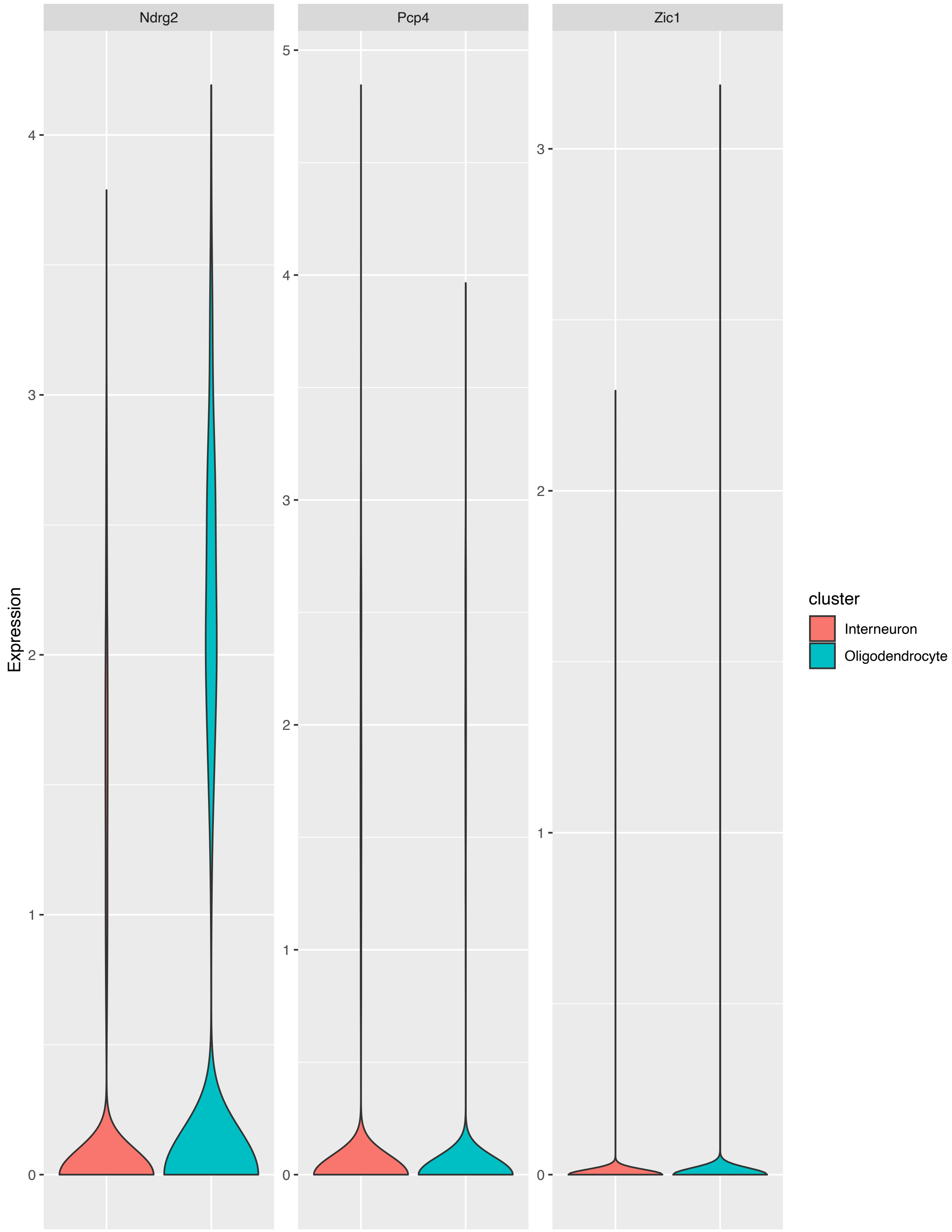

Ndrg2
Pcp4
Zic1
Expression
0
1
2
3
4
5
cluster
Interneuron
Oligodendrocyte

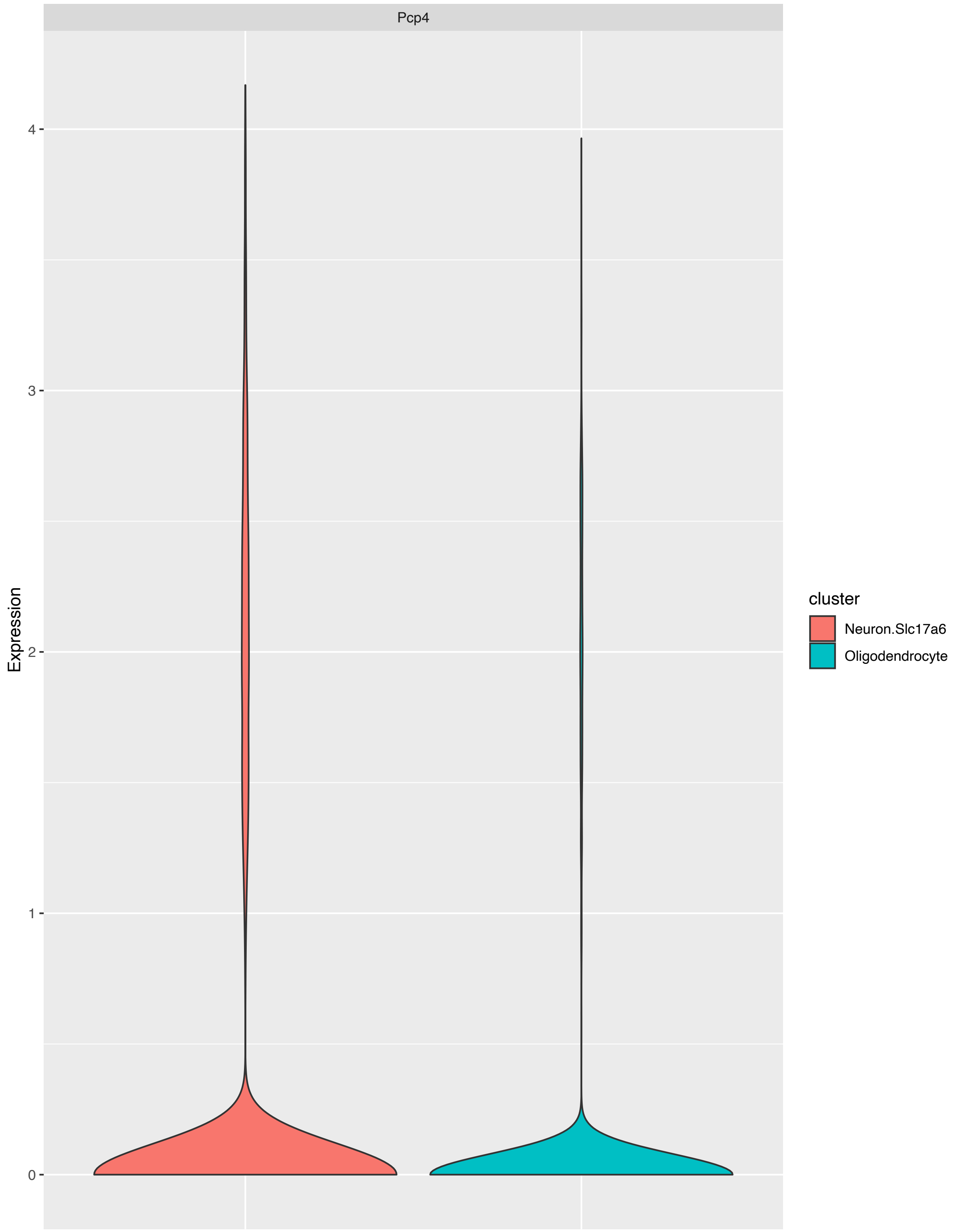
Pcp4
Expression
4
3
2
1
0
cluster
Neuron.Slc17a6
Oligodendrocyte

**C**

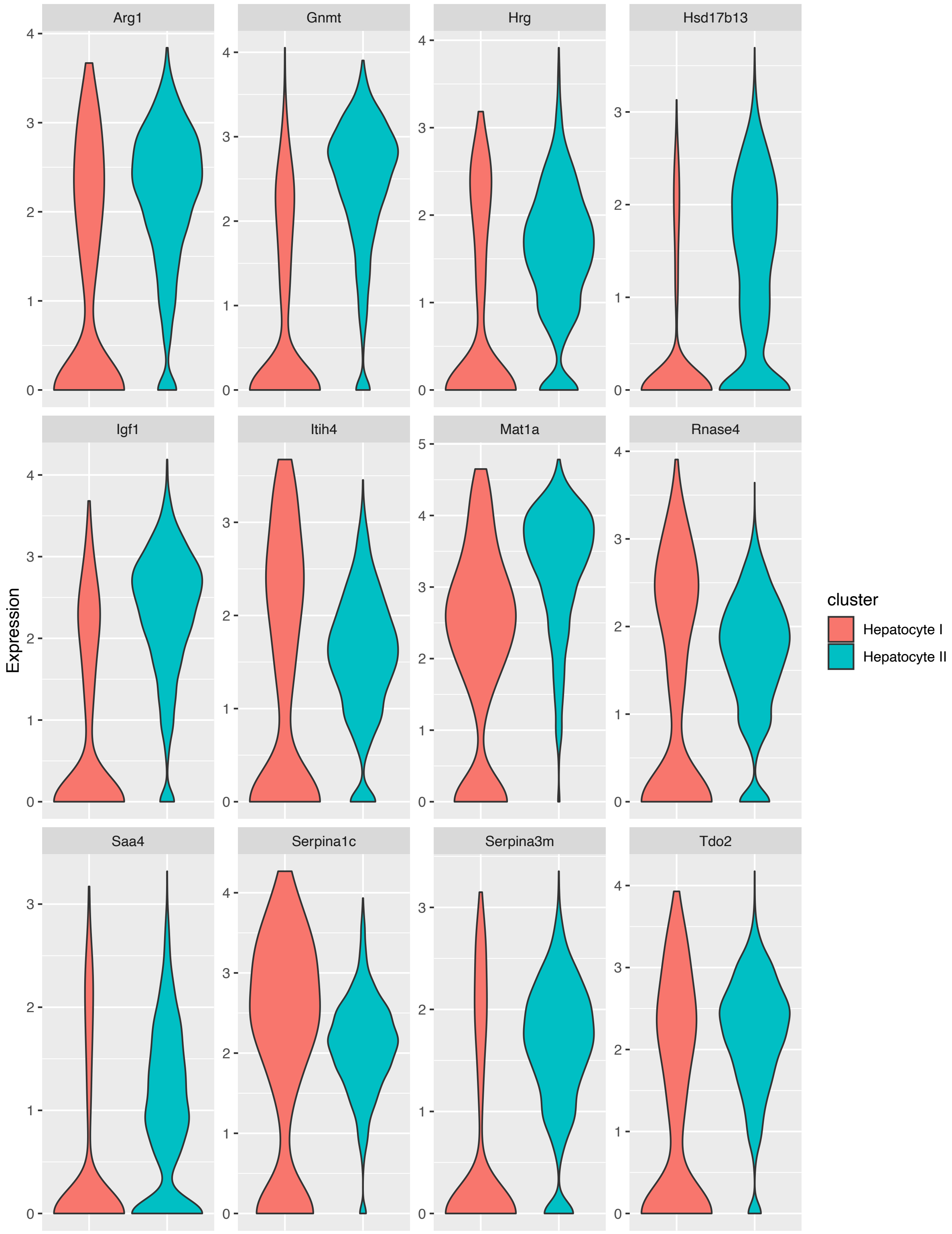

Arg1
Gnmt
Hrg
Hsd17b13
Igf1
Itih4
Mat1a
Rnase4
Saa4
Serpina1c
Serpina3m
Tdo2
Expression
cluster
Hepatocyte I
Hepatocyte II

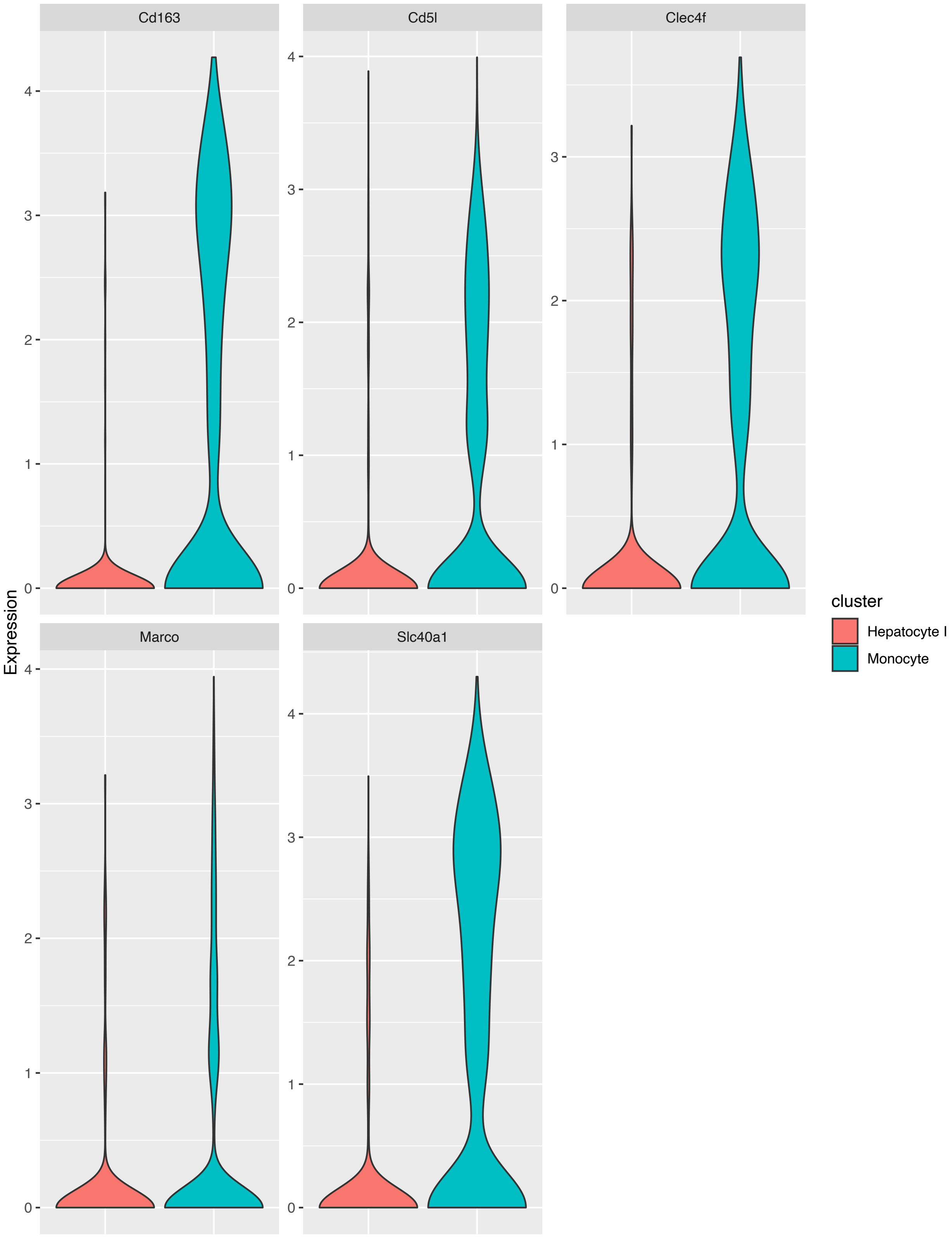
Cd163
Cd5l
Clec4f
Marco
Slc40a1
Expression
0
1
2
3
4
cluster
Hepatocyte I
Monocyte

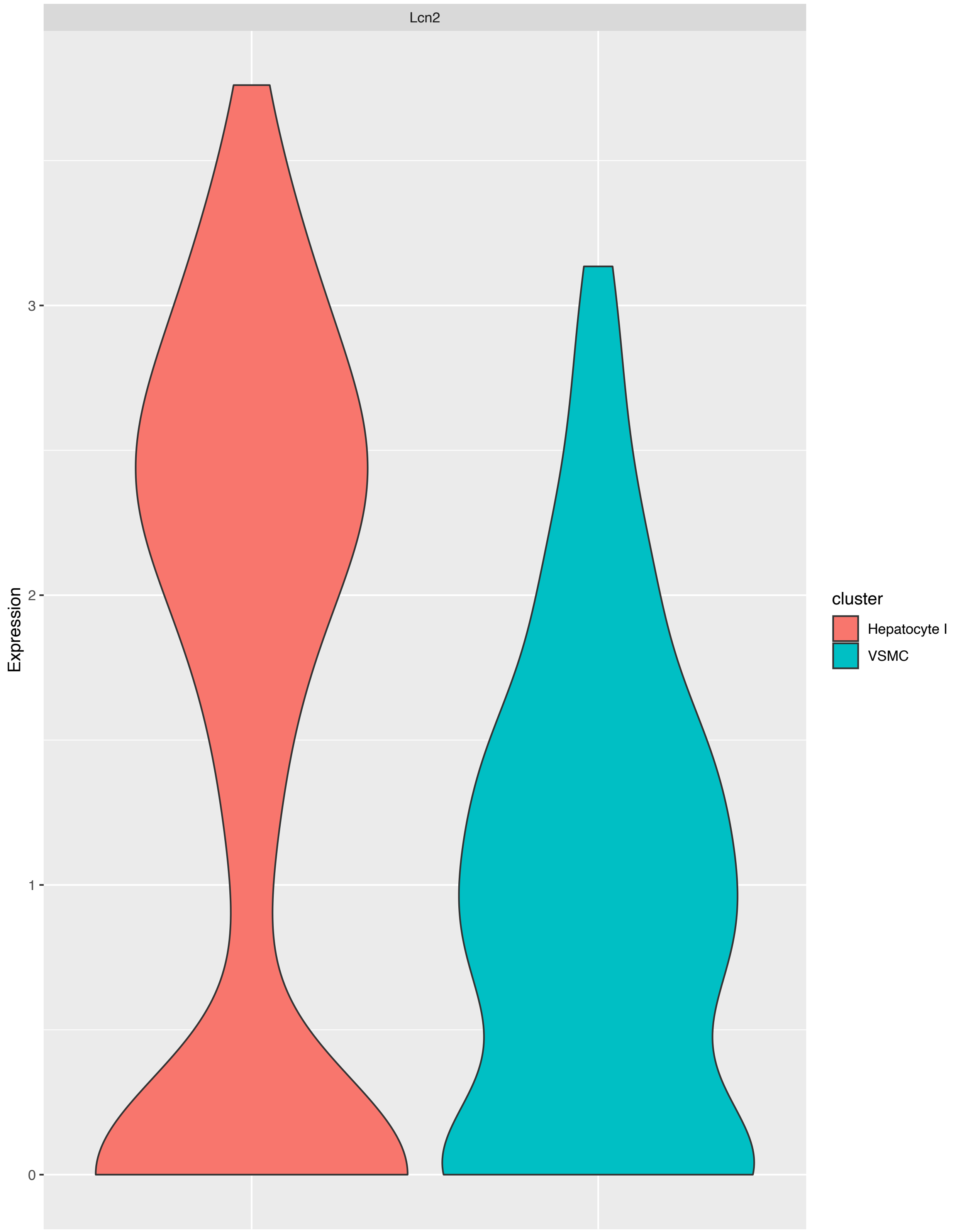
Lcn2
Expression
0
1
2
3
cluster
Hepatocyte I
VSMC

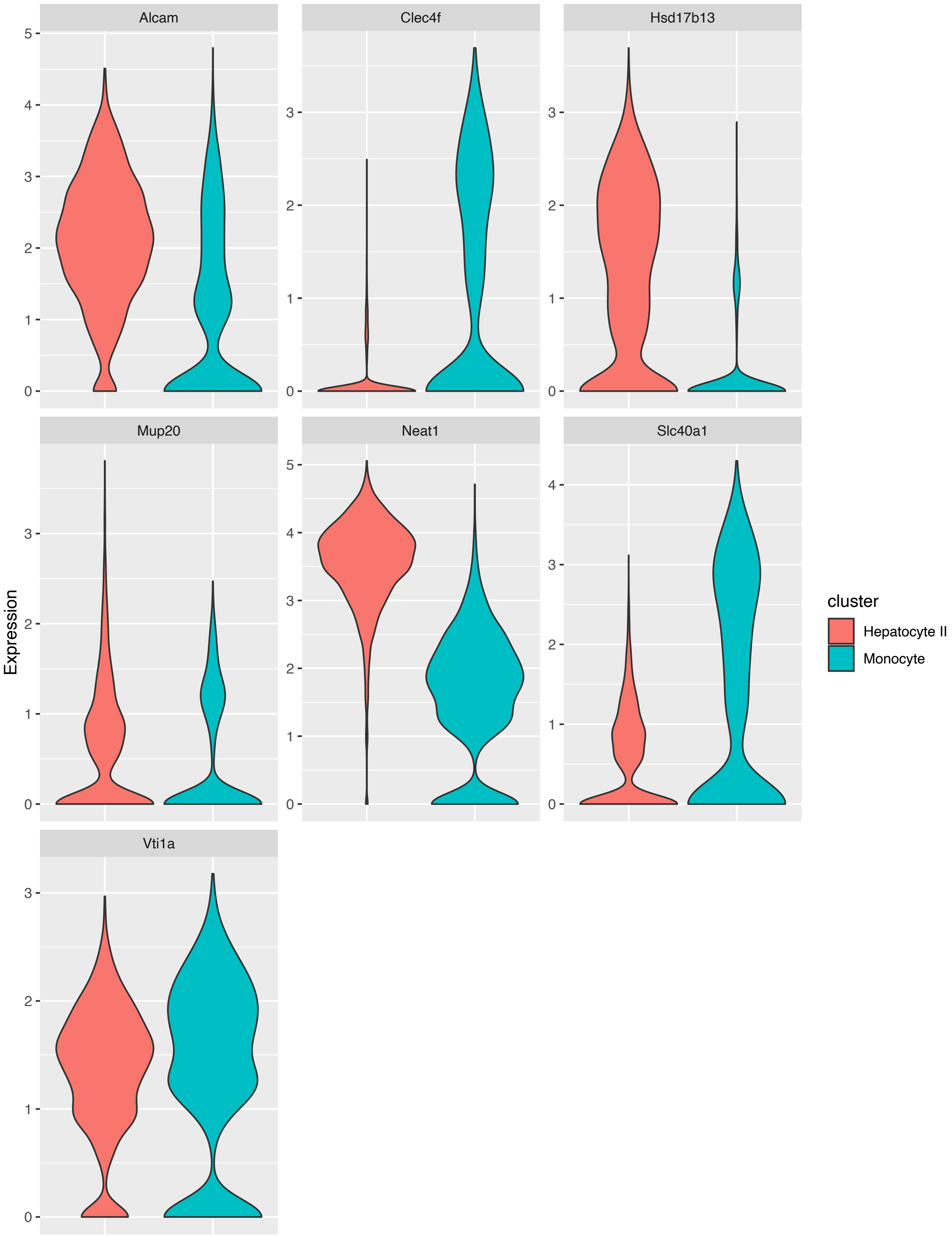

Alcam
Clec4f
Hsd17b13
Mup20
Neat1
Slc40a1
Vti1a
Expression
cluster
Hepatocyte II
Monocyte

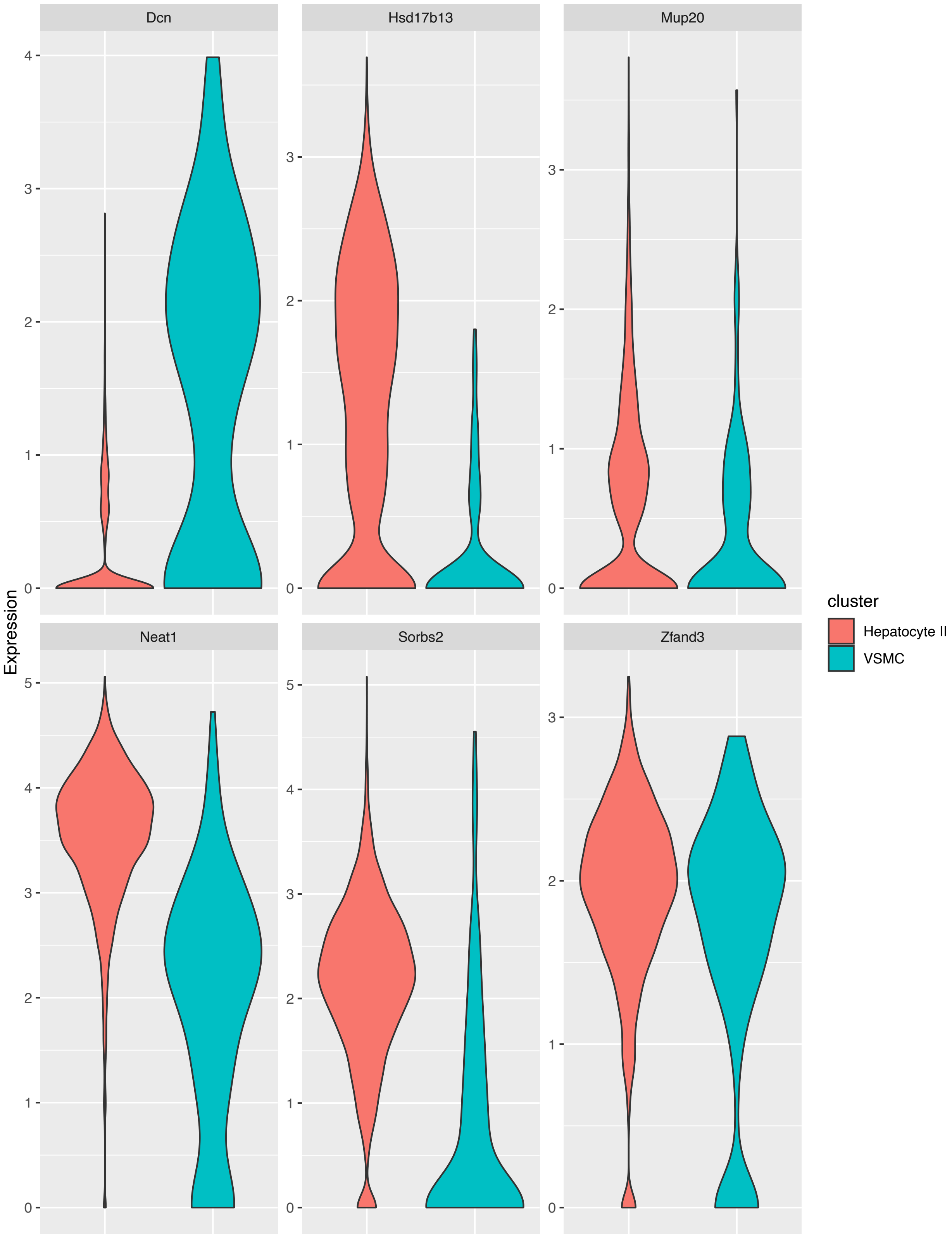

Dcn
Hsd17b13
Mup20
Neat1
Sorbs2
Zfand3
Expression
cluster
Hepatocyte II
VSMC

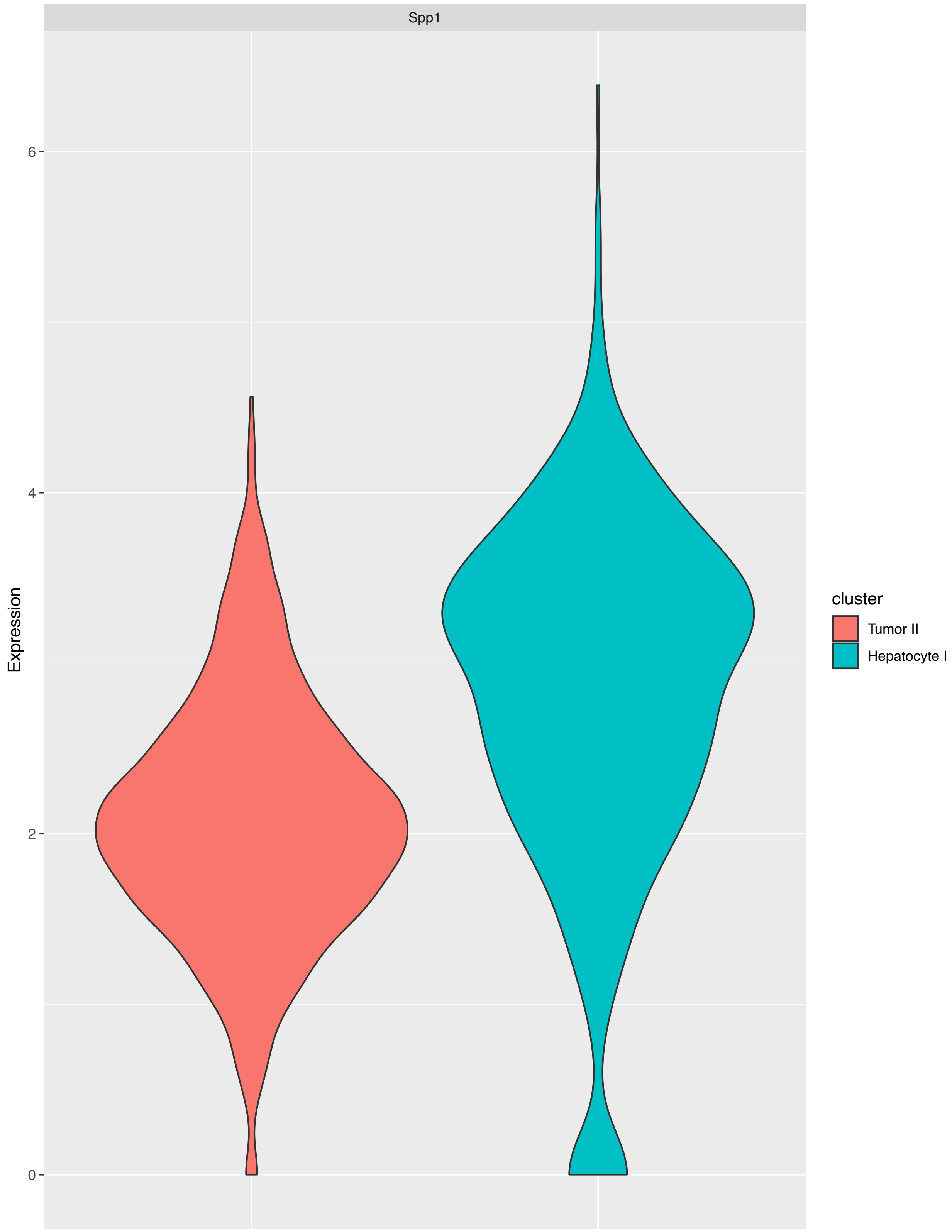

Spp1
Expression
6
4
2
0
cluster
Tumor II
Hepatocyte I

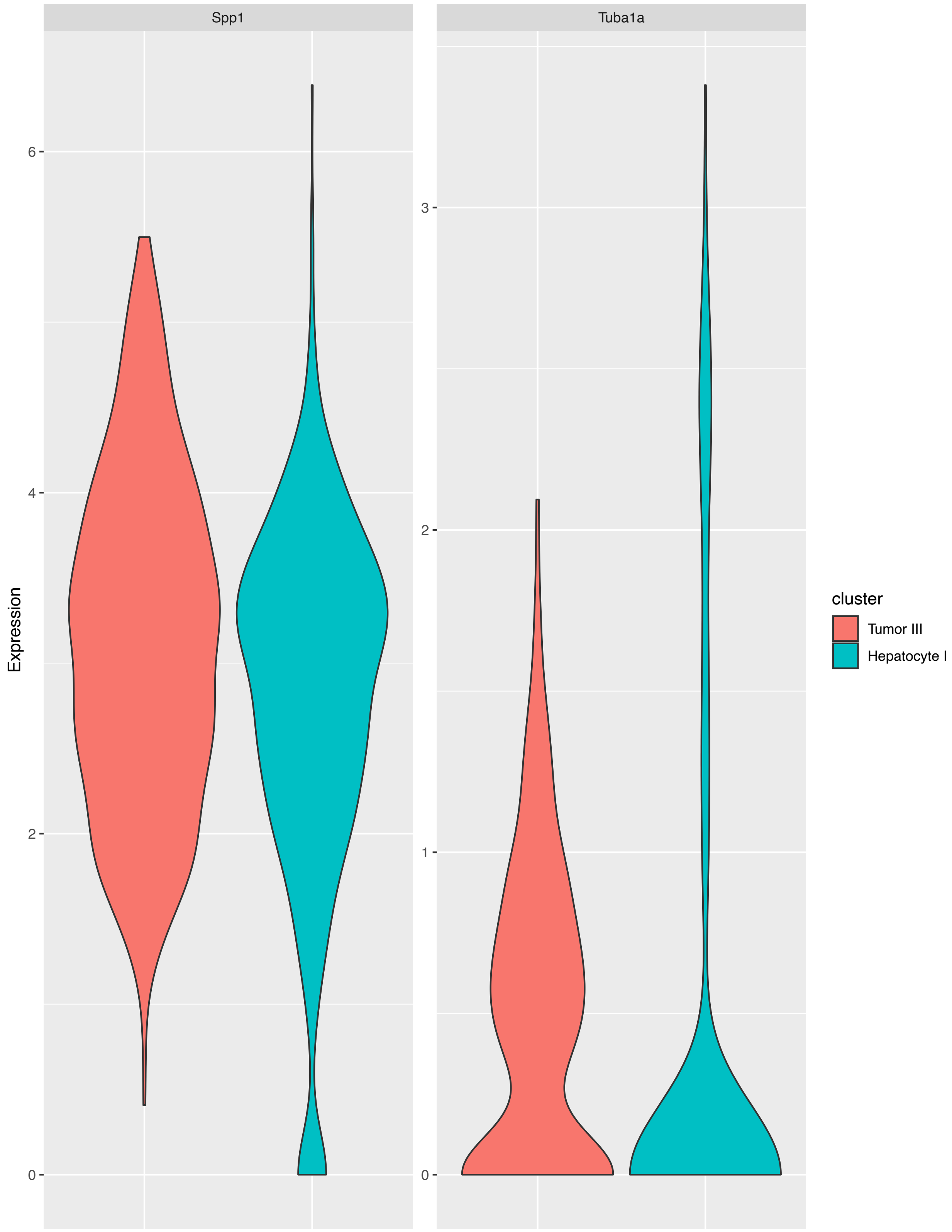
Spp1
Tuba1a
Expression
0
2
4
6
0
1
2
3
cluster
Tumor III
Hepatocyte I

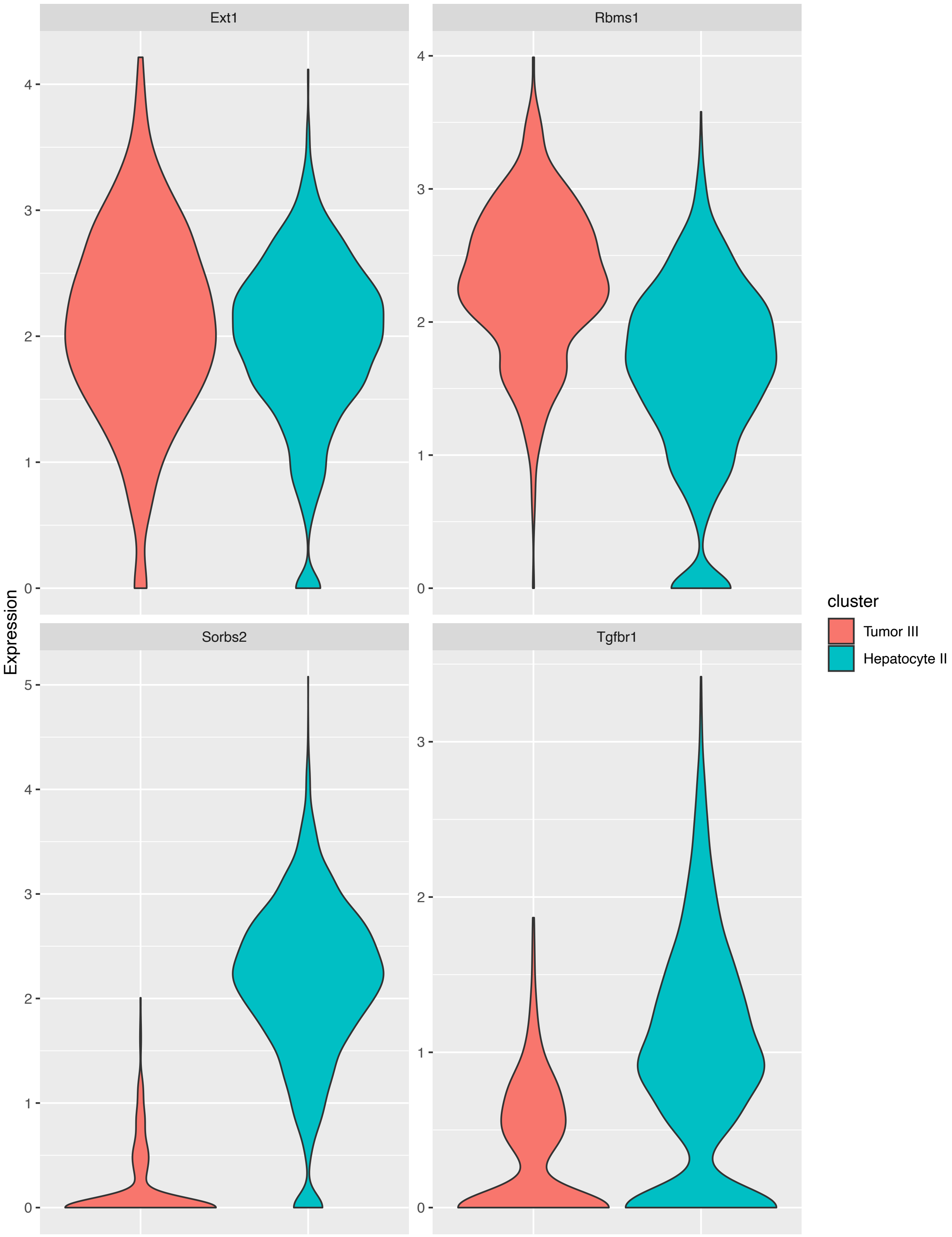
Ext1
Rbms1
Sorbs2
Tgfbr1
Expression
0
1
2
3
4
5
cluster
Tumor III
Hepatocyte II

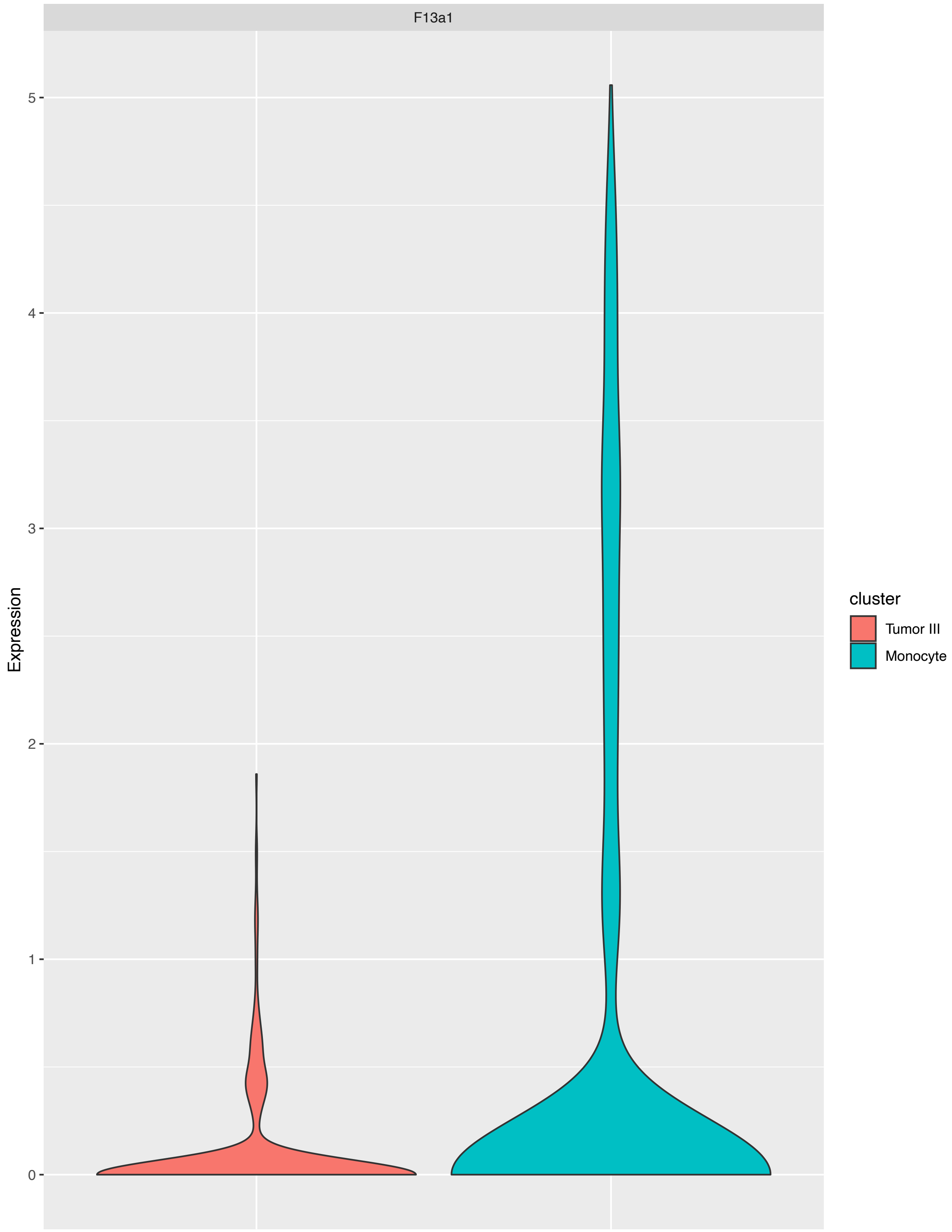
F13a1
Expression
5
4
3
2
1
0
cluster
Tumor III
Monocyte

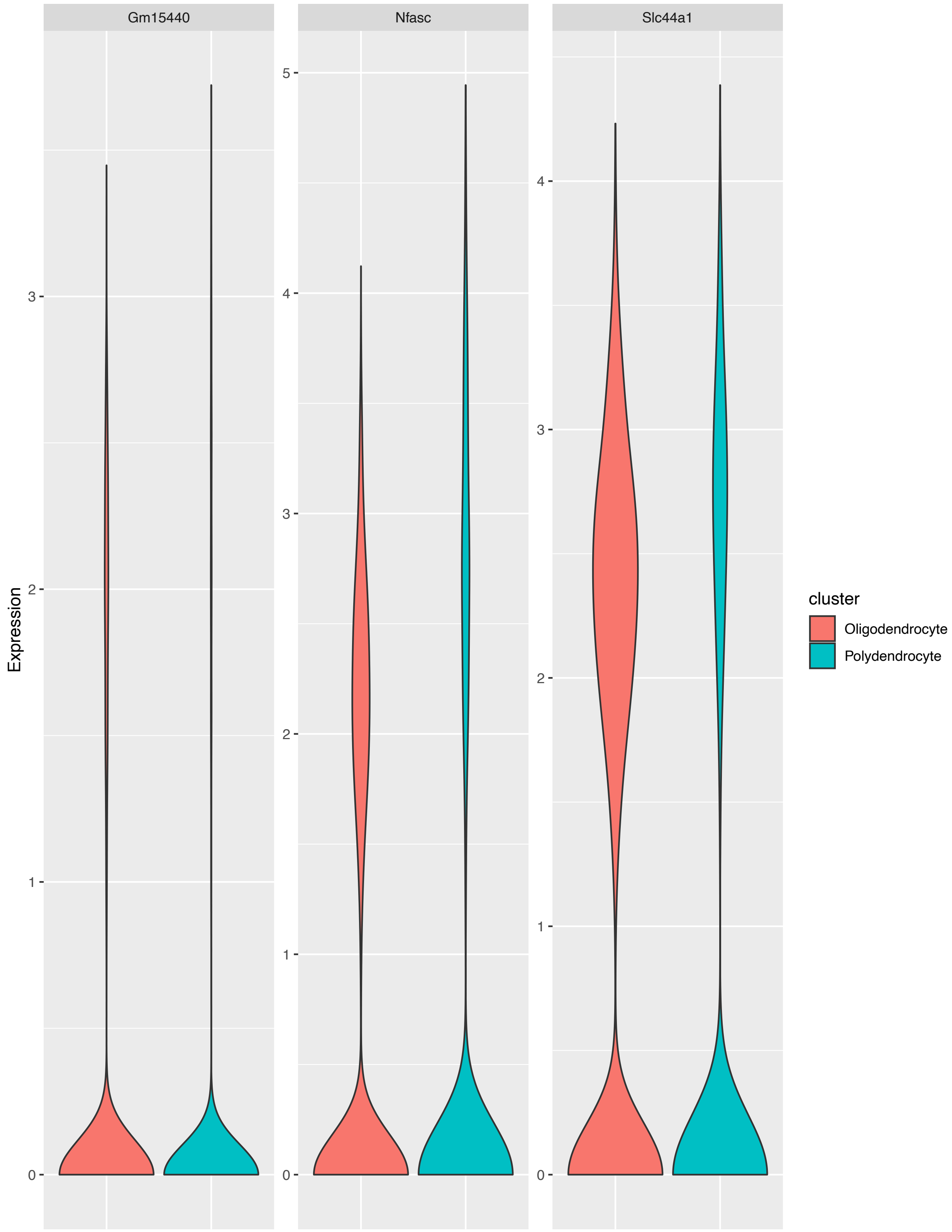
Gm15440
Nfasc
Slc44a1
Expression
0
1
2
3
4
5
cluster
Oligodendrocyte
Polydendrocyte

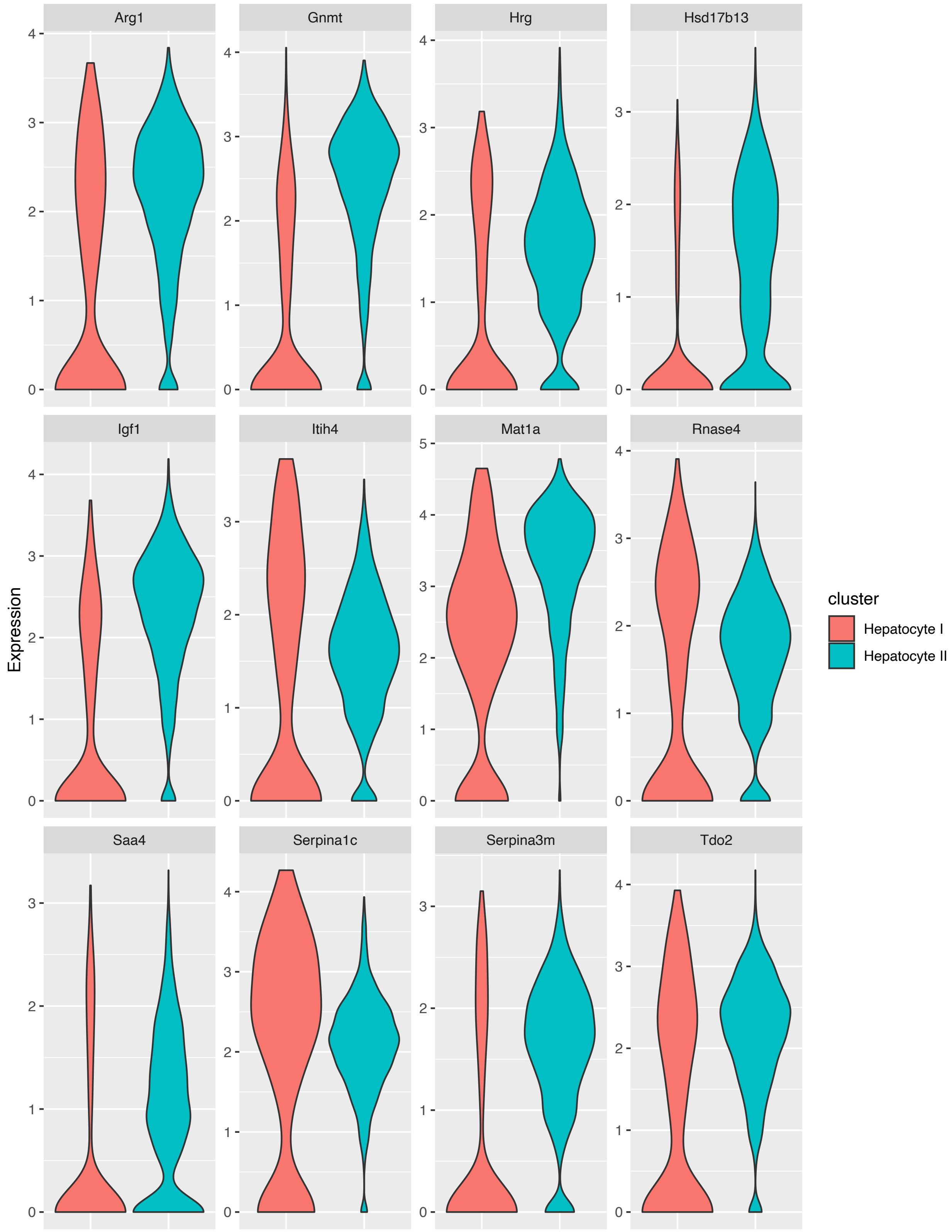
Arg1
Gnmt
Hrg
Hsd17b13
Igf1
Itih4
Mat1a
Rnase4
Saa4
Serpina1c
Serpina3m
Tdo2
Expression
cluster
Hepatocyte I
Hepatocyte II

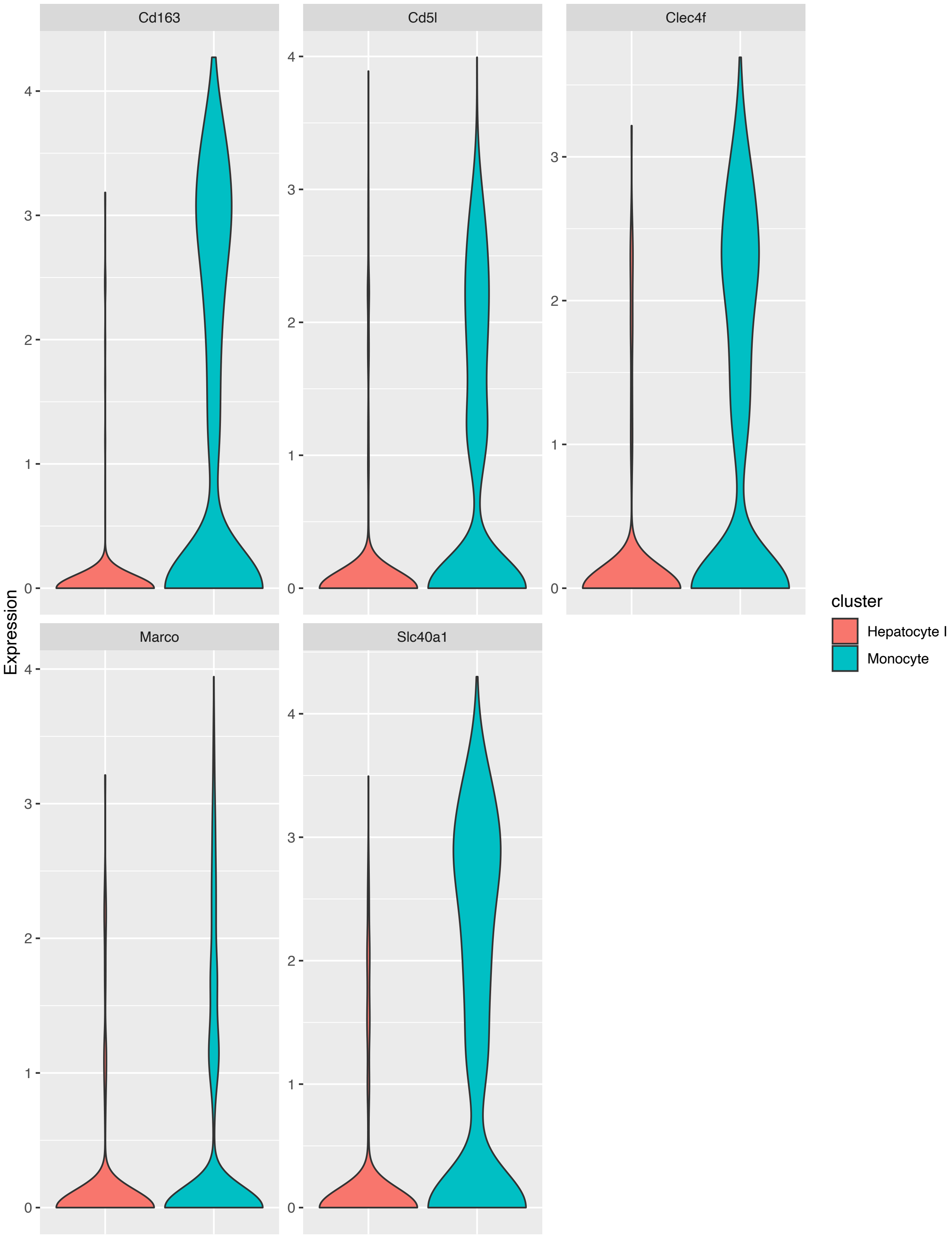

Cd163
Cd5l
Clec4f
Marco
Slc40a1
Expression
0
1
2
3
4
cluster
Hepatocyte I
Monocyte

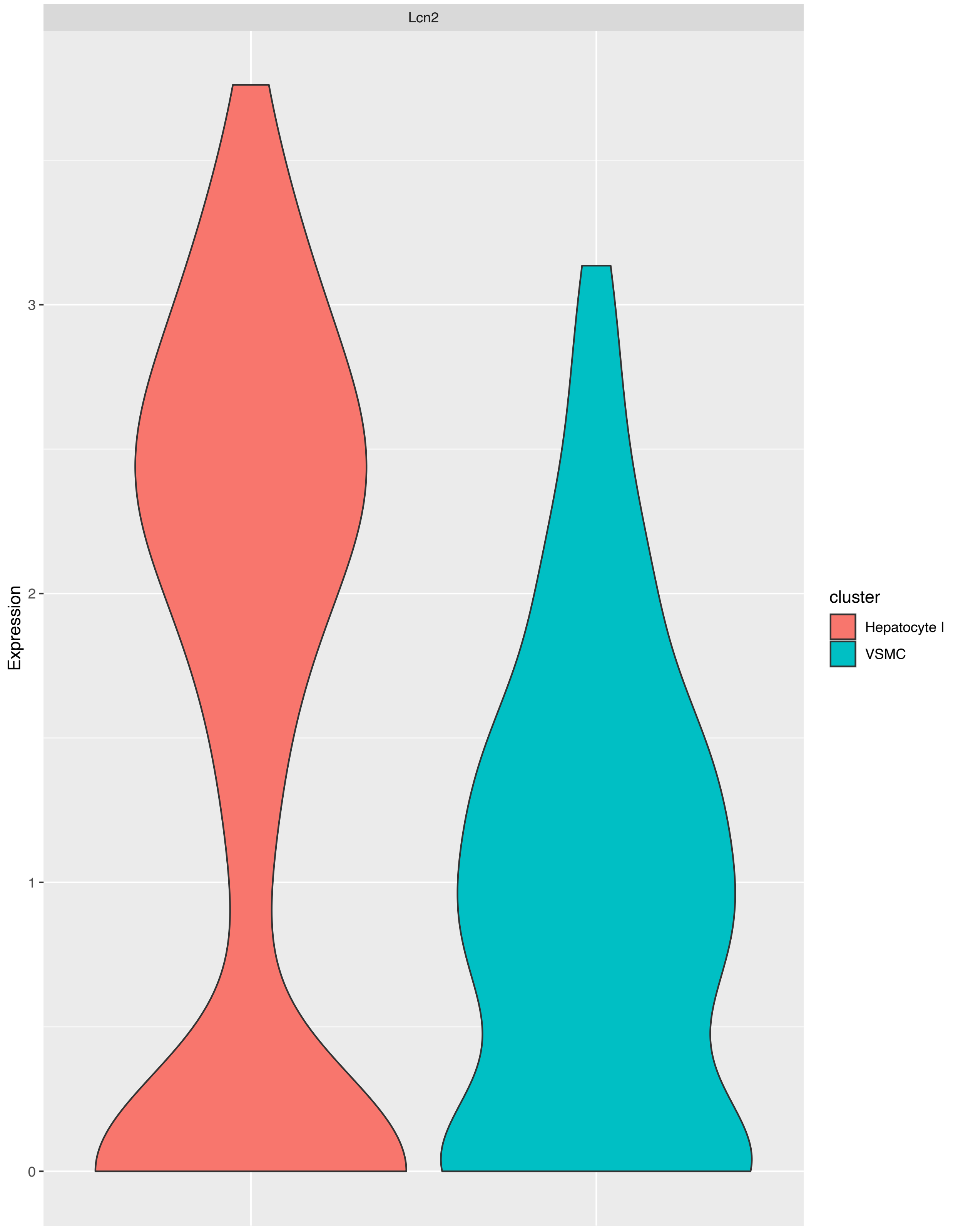
Lcn2
Expression
0
1
2
3
cluster
Hepatocyte I
VSMC

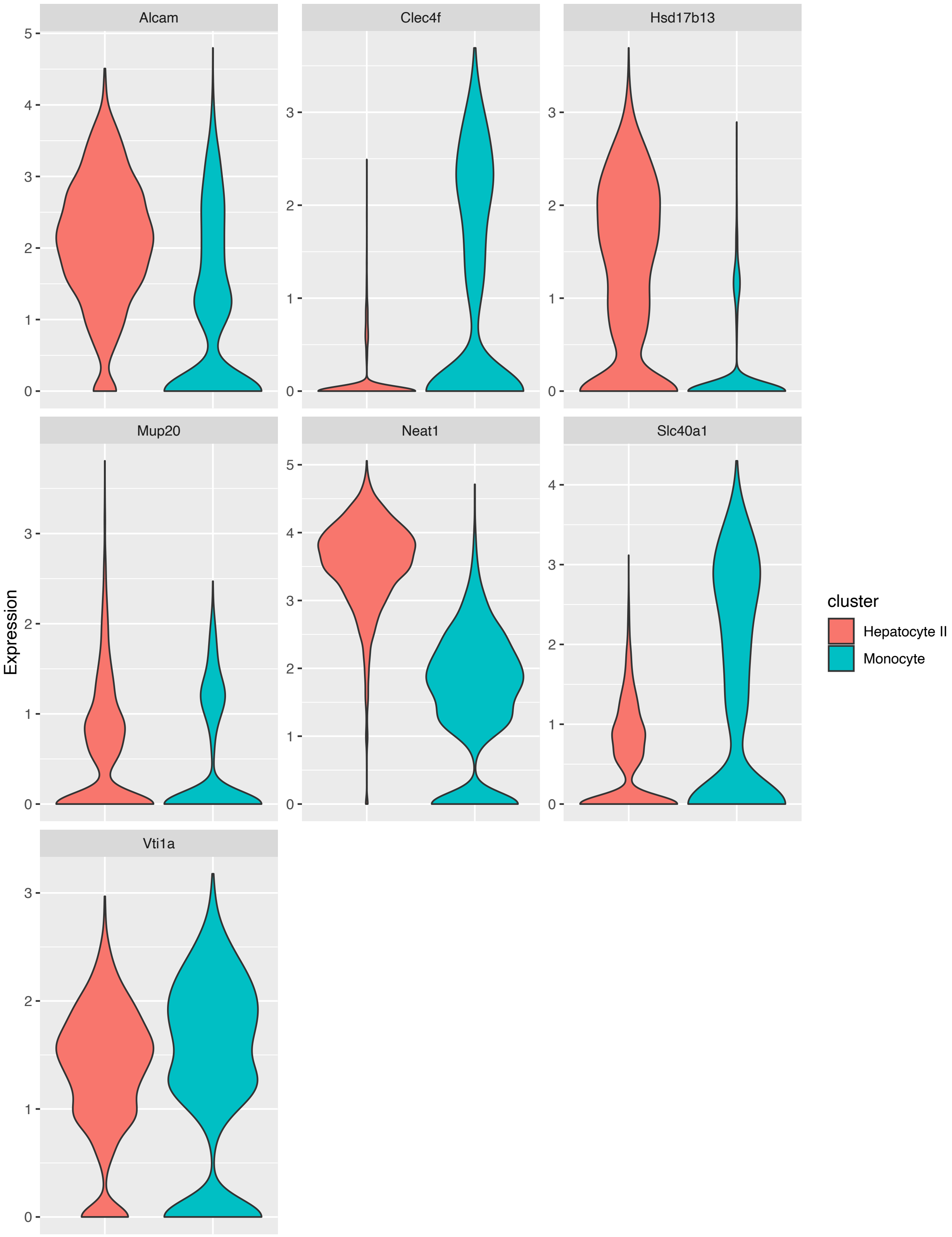
Alcam
Clec4f
Hsd17b13
Mup20
Neat1
Slc40a1
Vti1a
Expression
cluster
Hepatocyte II
Monocyte

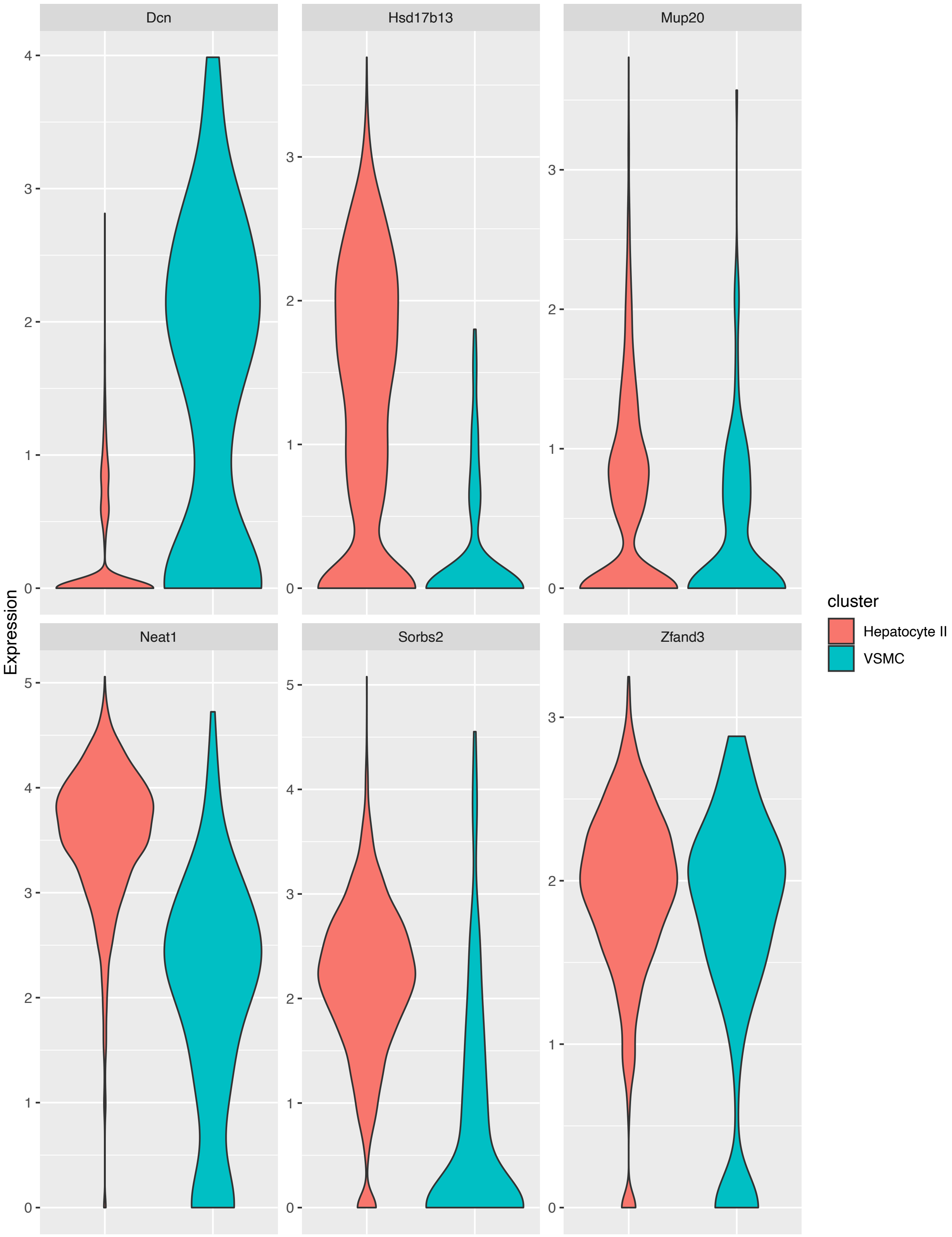
Dcn
Hsd17b13
Mup20
Neat1
Sorbs2
Zfand3
Expression
cluster
Hepatocyte II
VSMC

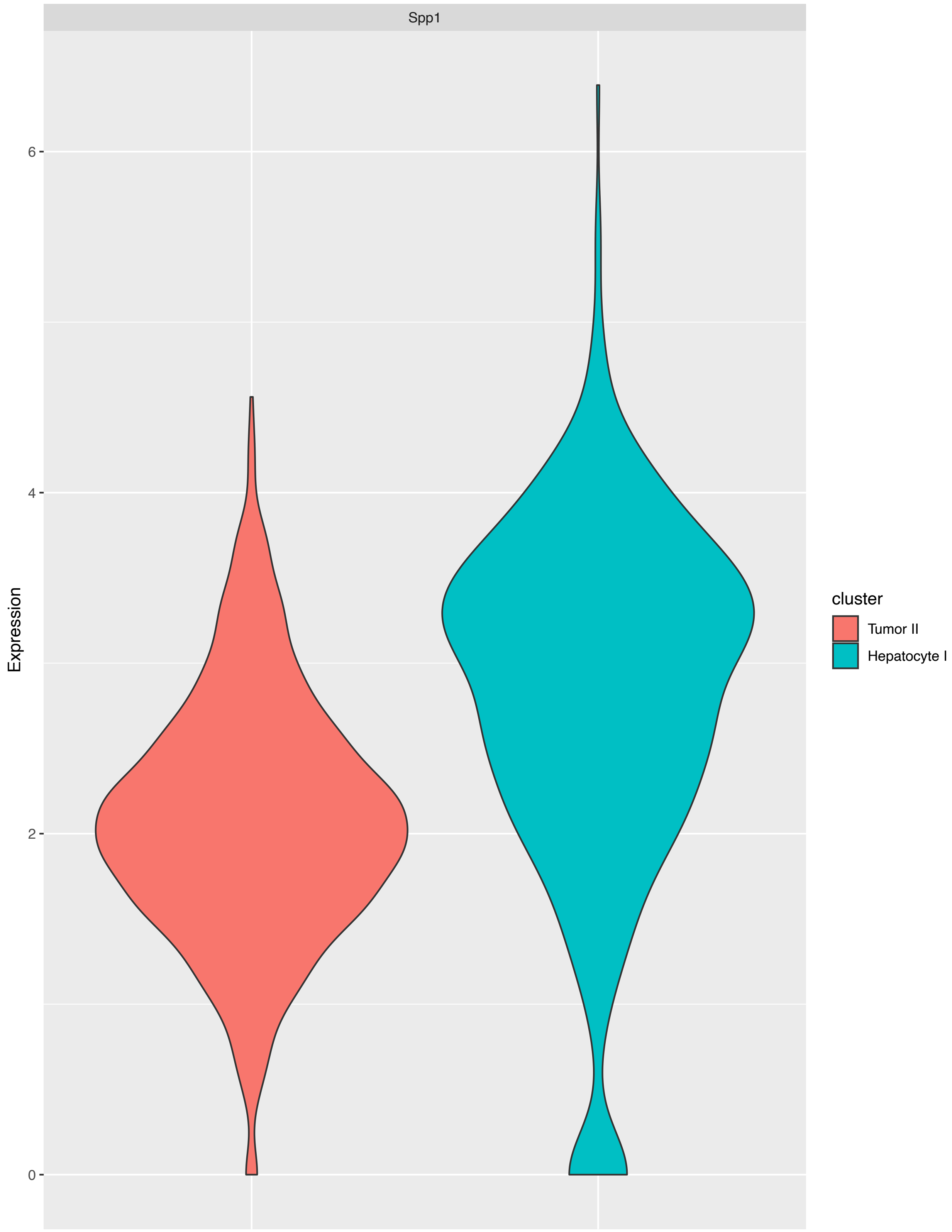
Spp1
Expression
0
2
4
6
cluster
Tumor II
Hepatocyte I

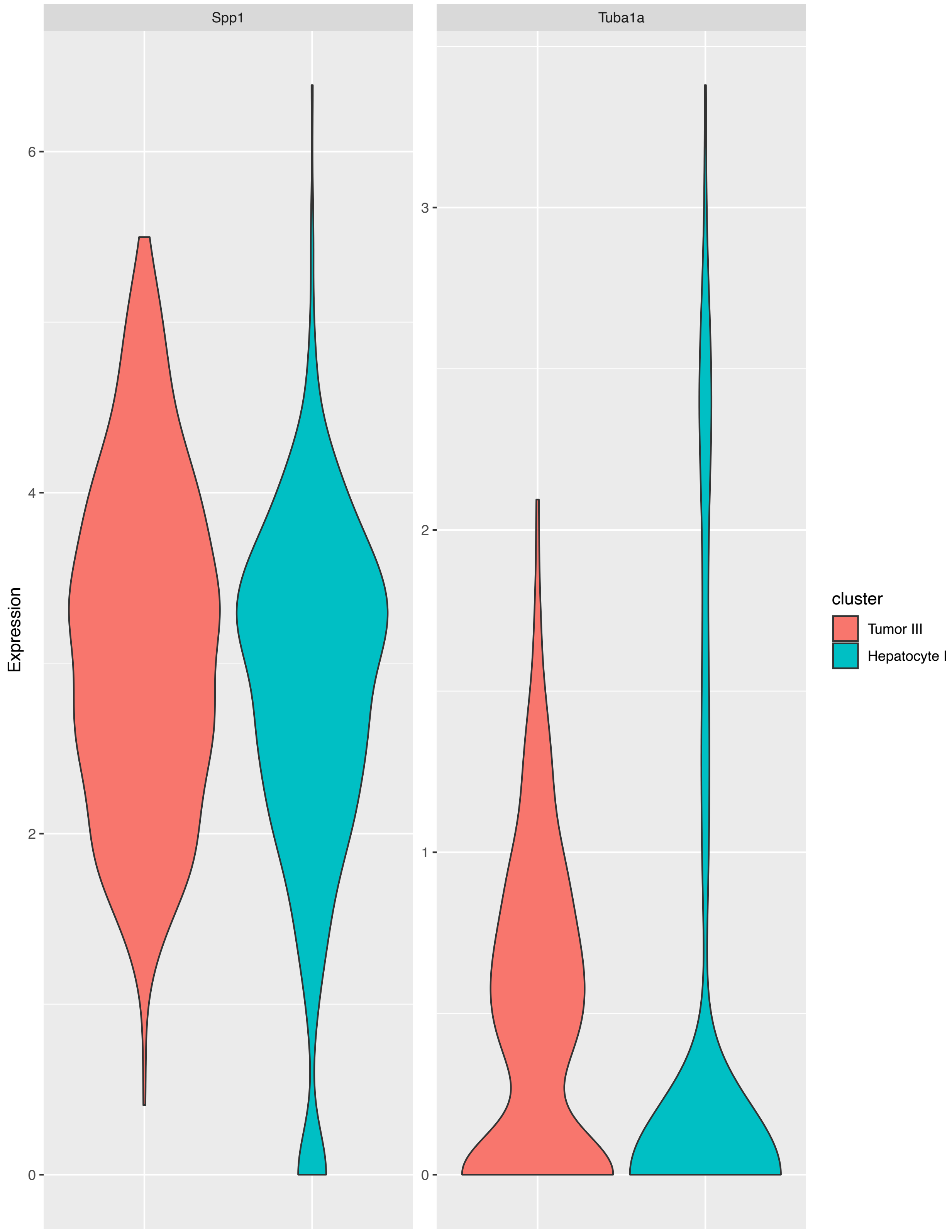

Spp1
Tuba1a
Expression
6
4
2
0
3
2
1
0
cluster
Tumor III
Hepatocyte I

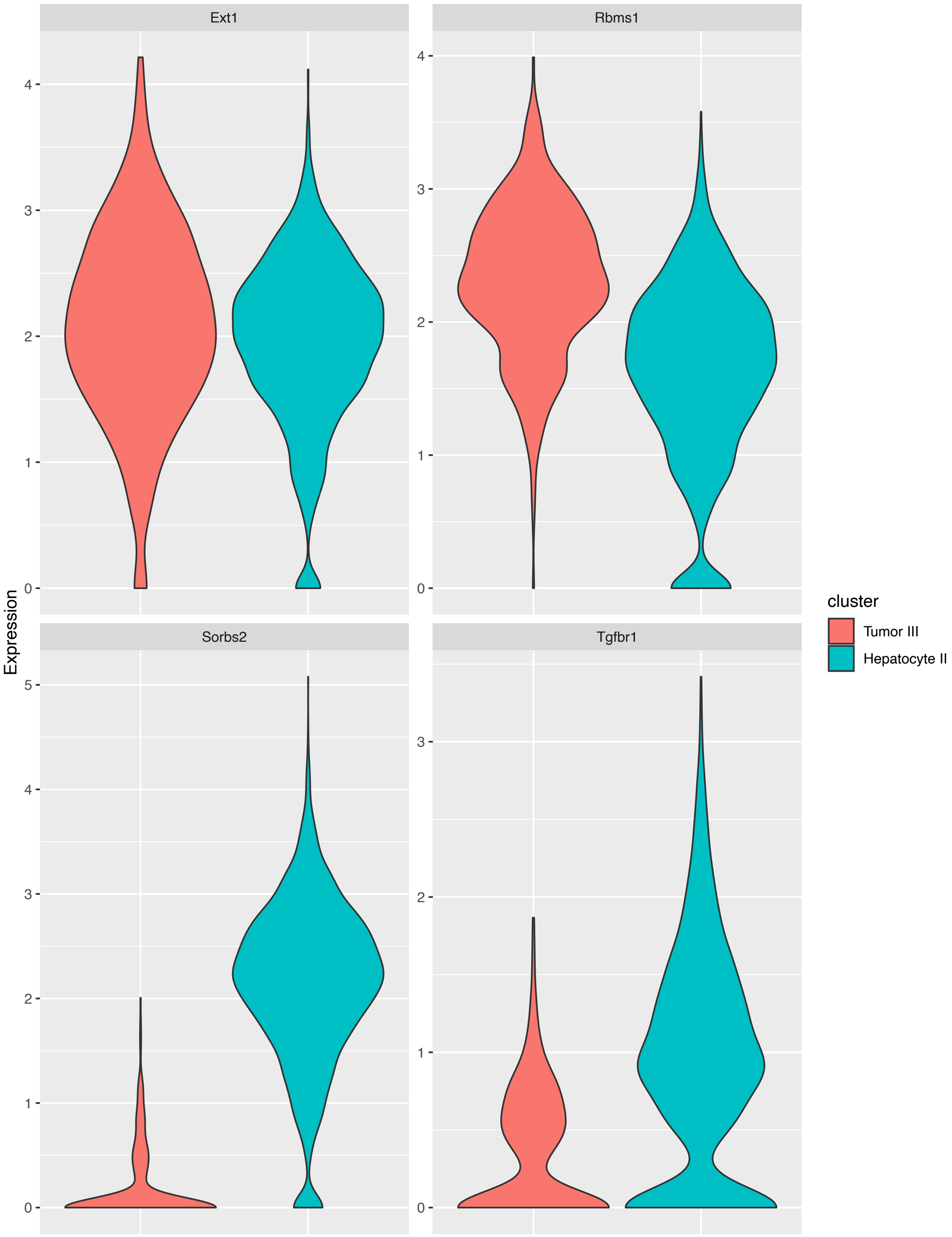

Ext1
Rbms1
Sorbs2
Tgfbr1
Expression
0
1
2
3
4
5
cluster
Tumor III
Hepatocyte II

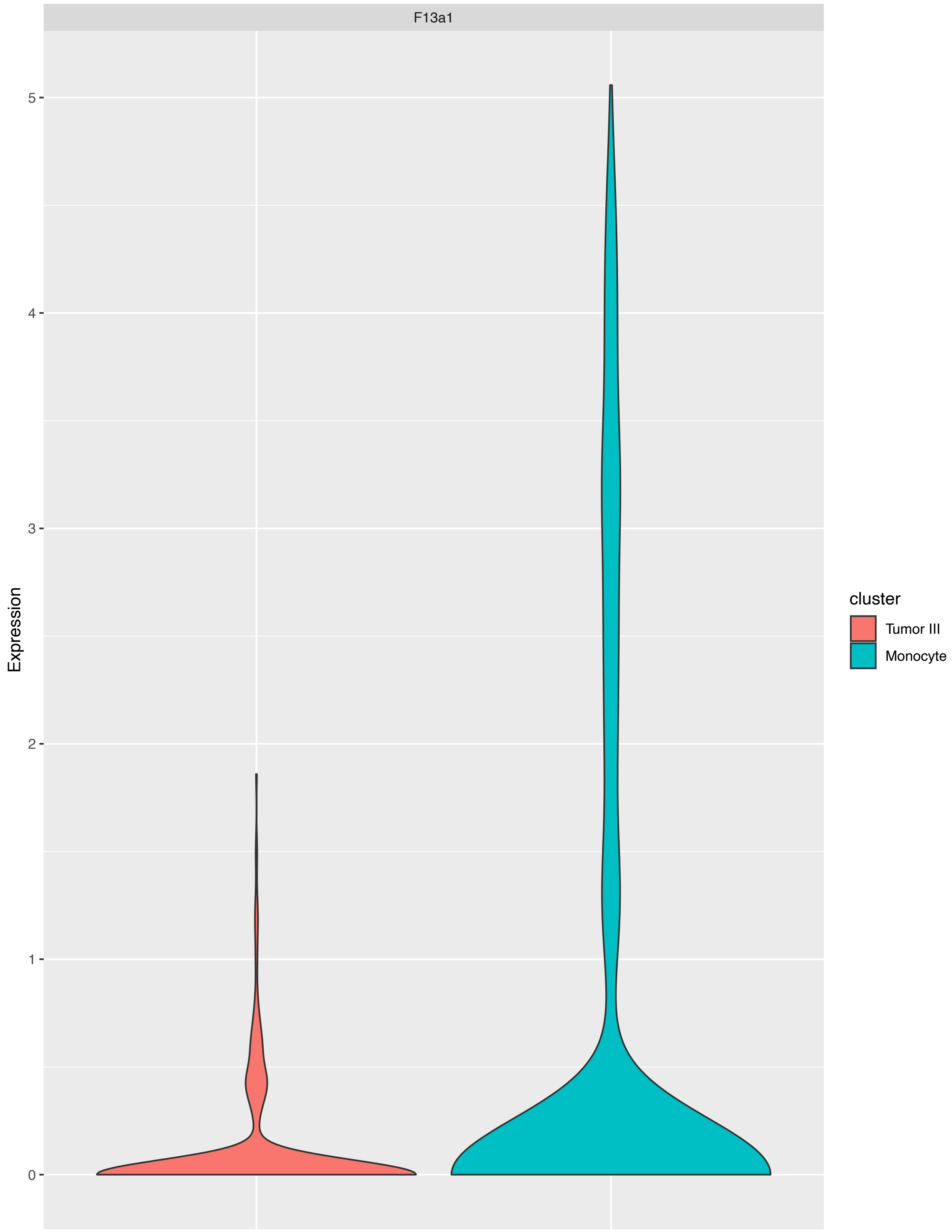
F13a1
Expression
5
4
3
2
1
0
cluster
Tumor III
Monocyte

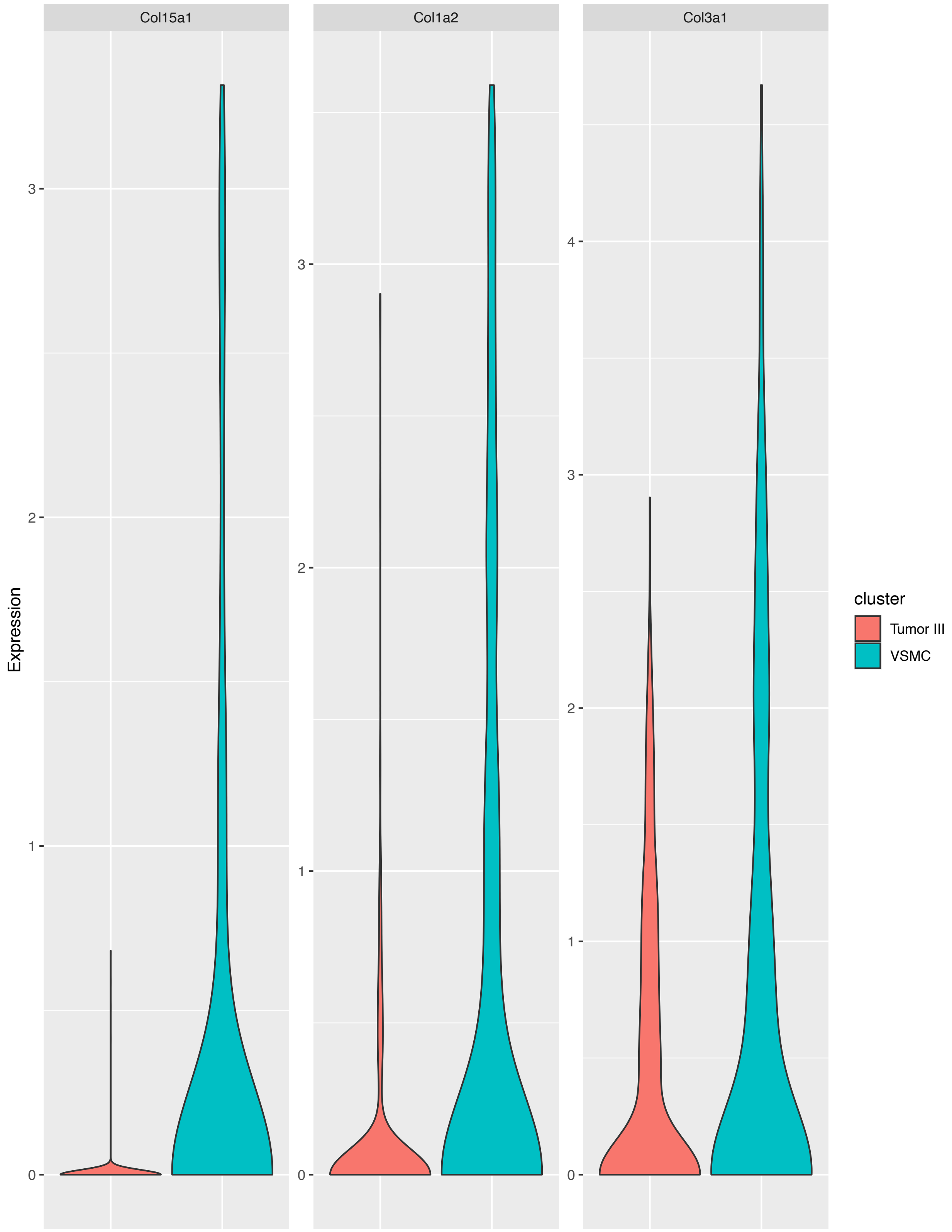
Col15a1
Col1a2
Col3a1
Expression
0
1
2
3
4
cluster
Tumor III
VSMC

**Appendix Figure S9. Origins of the expression of neighbor-dependent genes identified from single cell/nucleus data.**

A The violin plots show the expression of neighbor-dependent genes in scRNA-seq data from a mouse embryo. 28 up-regulated genes were identified from 9 heterotypic pairs. The violin plots display the distributions of the expression of 26 up-regulated genes except for *Crygs* because the scRNA-seq dataset does not contain its expression level.

B The violin plots show the expression of neighbor-dependent genes in scRNA-seq data from mouse hippocampus. 155 up-regulated genes were identified from 21 heterotypic pairs.

C The violin plots show the expression of neighbor-dependent genes in snRNA-seq data from mouse liver cancer. 42 up-regulated genes were identified from 10 heterotypic pairs.
