# Supplementary material for: CellNeighborEX: deciphering neighbor‐dependent gene expression from spatial transcriptomics data
Source: Mol Syst Biol. 2023 Oct 10;19(11):e11670. doi: 10.15252/msb.202311670 (PMC10632736; doi:10.15252/msb.202311670)
Supplement: Supplementary file 2 — Expanded View Figures PDF [file MSB-19-e11670-s006.pdf]

Expanded View Figures

Figure EV1. Neighbor dependent-genes identified by CellNeighborEX in mouse embryo seqFISH data.

A Gut tube cells adjacent to Neural crest cells (Gut tube/Neural crest) ($n = 694$) were compared with Gut tube cells proximal to other Gut tube cells (Gut tube/Gut tube; $n = 33$; log ratio > 0.4, FDR < 0.05). The statistical test was chosen among the two tailed Student's *t*-test, Welch's *t*-test, or Wilcoxon rank sum test depending on the sample size and heterogeneity of variance test. The volcano plot shows 23 up-regulated including *Pitx1* and 21 down-regulated genes including *Foxa1* in Gut tube/Neural crest.

B The spatial visualization displays that Gut tube cells adjacent to Neural crest more highly express *Pitx1*.

C For genes up-regulated by cell contact in the mouse embryo seqFISH data, GO analysis shows that the GO terms are associated with embryonic development. The bar plot presents top 20 GO terms.

© 2023 The Authors

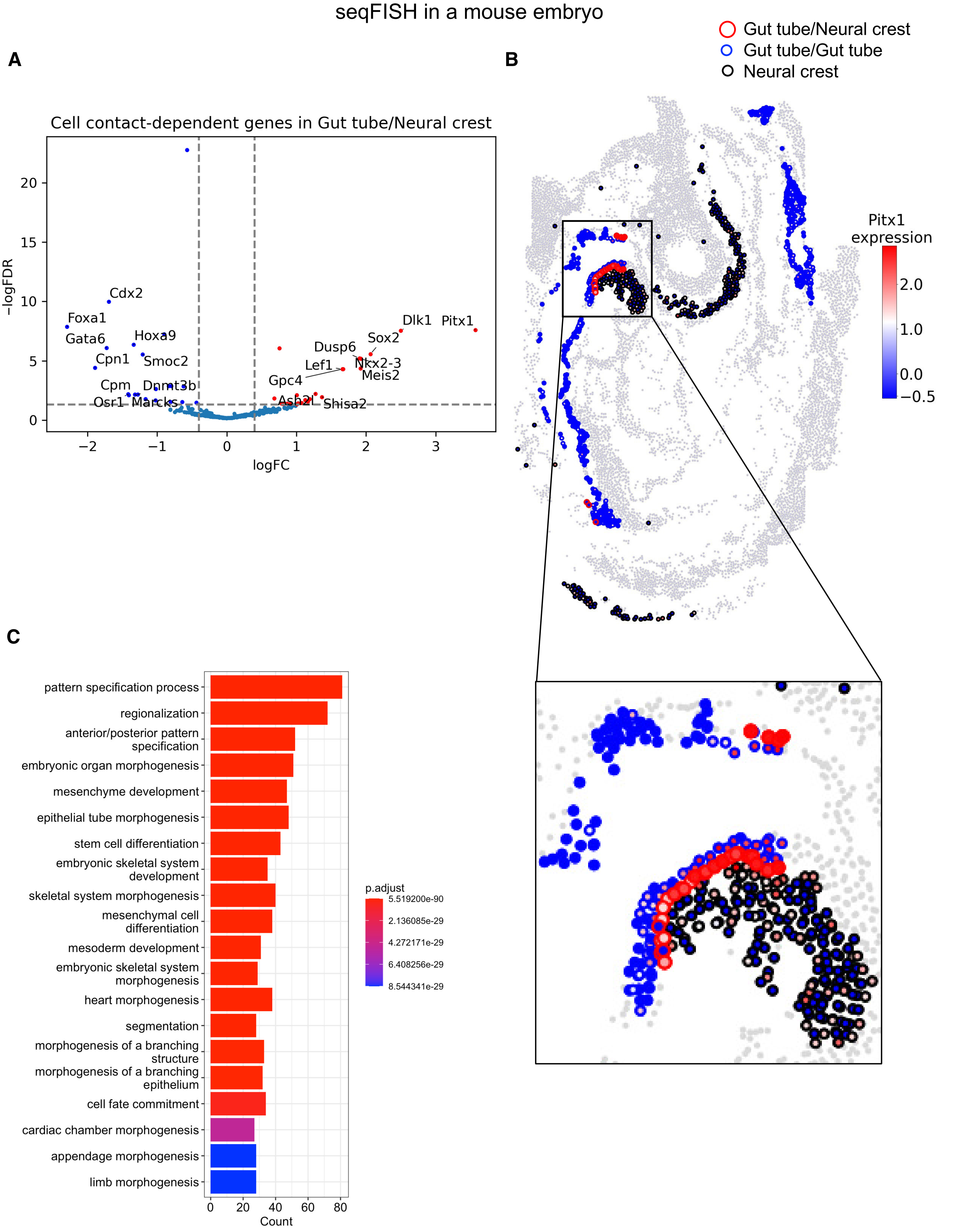

Figure EV1.

© 2023 The Authors

Figure EV2. Transcriptomic change due to direct cell contact in mouse embryo Slide-seq data.

A The heterotypic spots of Endothelial and Lens cells (En + L) (n = 120) were compared with the respective homotypic spots (En [n = 85], L [=52]; log ratio > 0.4, P-value < 0.01) and additionally with the artificial heterotypic spots (FDR < 0.01). The statistical test was chosen among the two tailed Student's t-test, Welch's t-test, or Wilcoxon rank sum test depending on the sample size and heterogeneity of variance test. The volcano plot displays 17 up-regulated genes including *Cd24a*. In the heatmap, the genes are more highly expressed in En + L. The heterotypic spots also express both En and L markers.

B The spatial visualization shows the higher expression level of *Cd24a* in En + L.

C In the mouse embryo scRNA-seq data, it was confirmed that *Cd24a* is expressed in En.

D In GO analysis, the GO terms for the genes up-regulated by neighbors are associated with embryonic development. The bar plot shows top 20 GO terms.

© 2023 The Authors

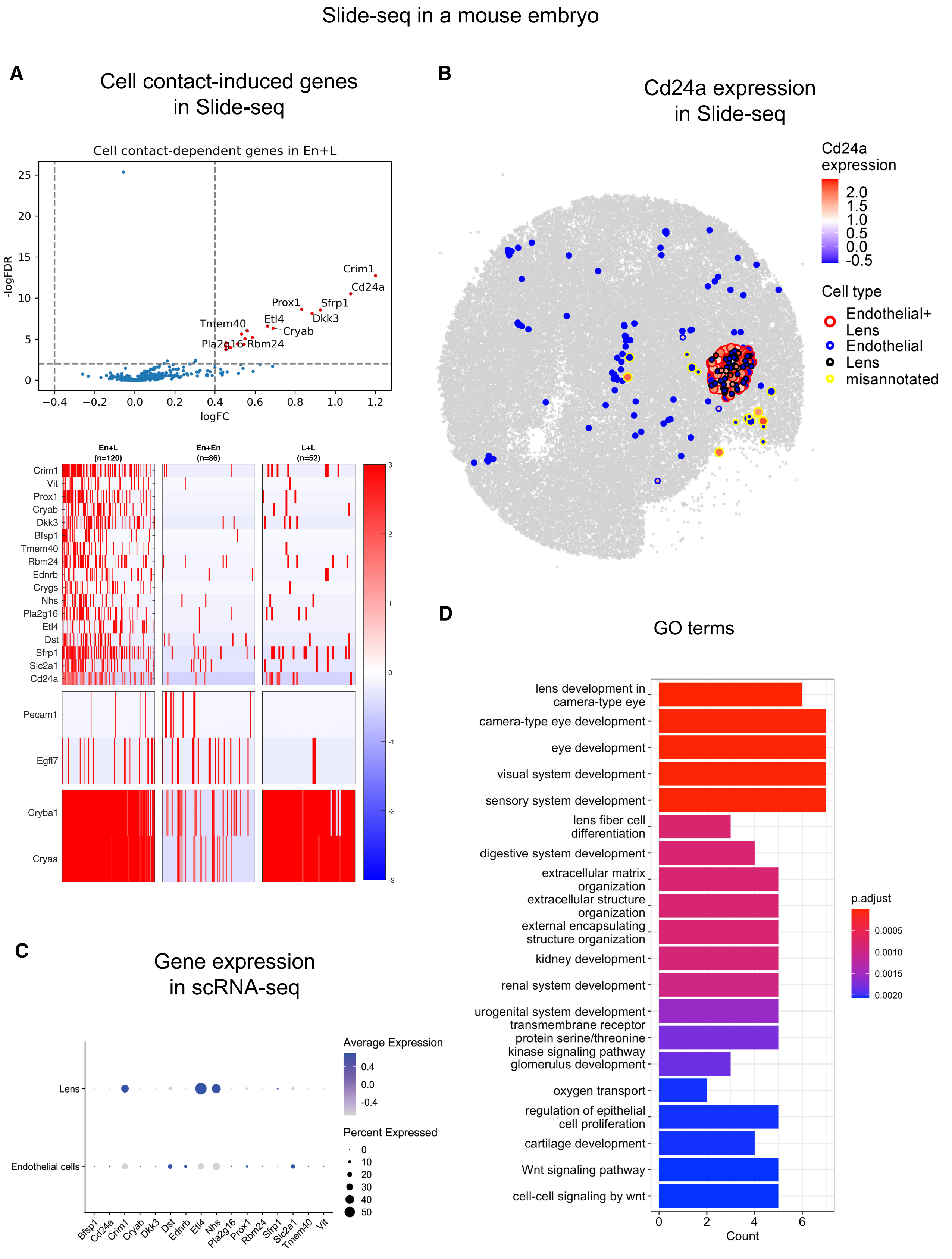

Figure EV2.

© 2023 The Authors

Figure EV3. CellNeighborEx identified genes influenced by TME.

A The heterotypic spots of Tumor III and Monocyte cells (Tumor III + Monocyte; $n = 742$) were compared with the respective homotypic spots (Tumor III [$n = 4,206$], Monocyte [$n = 218$]; log ratio > 0.4, P-value < 0.01) and additionally with the artificial heterotypic spots (FDR < 0.01). The statistical test was chosen among the two tailed Student's t-test, Welch's t-test, or Wilcoxon rank sum test depending on the sample size and heterogeneity of variance test. The volcano plot displays that *F13a1* is an up-regulated gene. In the heatmap, *F13a1* is more highly expressed in Tumor III + Monocyte. The heterotypic spots also express both Tumor III and Monocyte markers.

B The spatial visualization shows the higher expression level of *F13a1* in Tumor III + Monocyte.

C In the mouse liver cancer snRNA-seq data, it was confirmed that *F13a1* is mostly expressed in Monocyte cells.

D GO analysis in the mouse liver cancer data shows the terms associated with tumor metastasis. The bar plot presents top 20 GO terms.

© 2023 The Authors

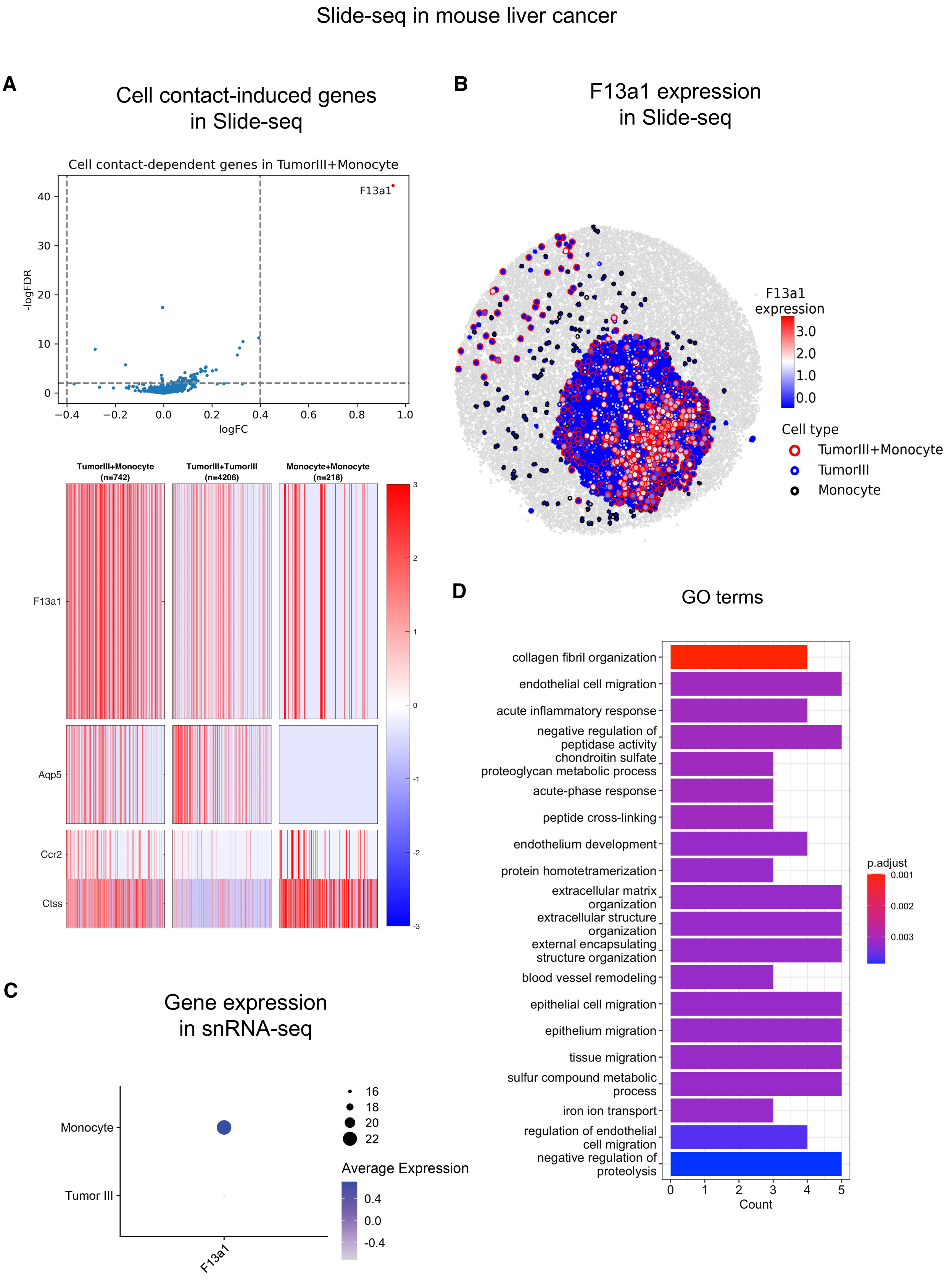

Figure EV3.

 © 2023 The Authors

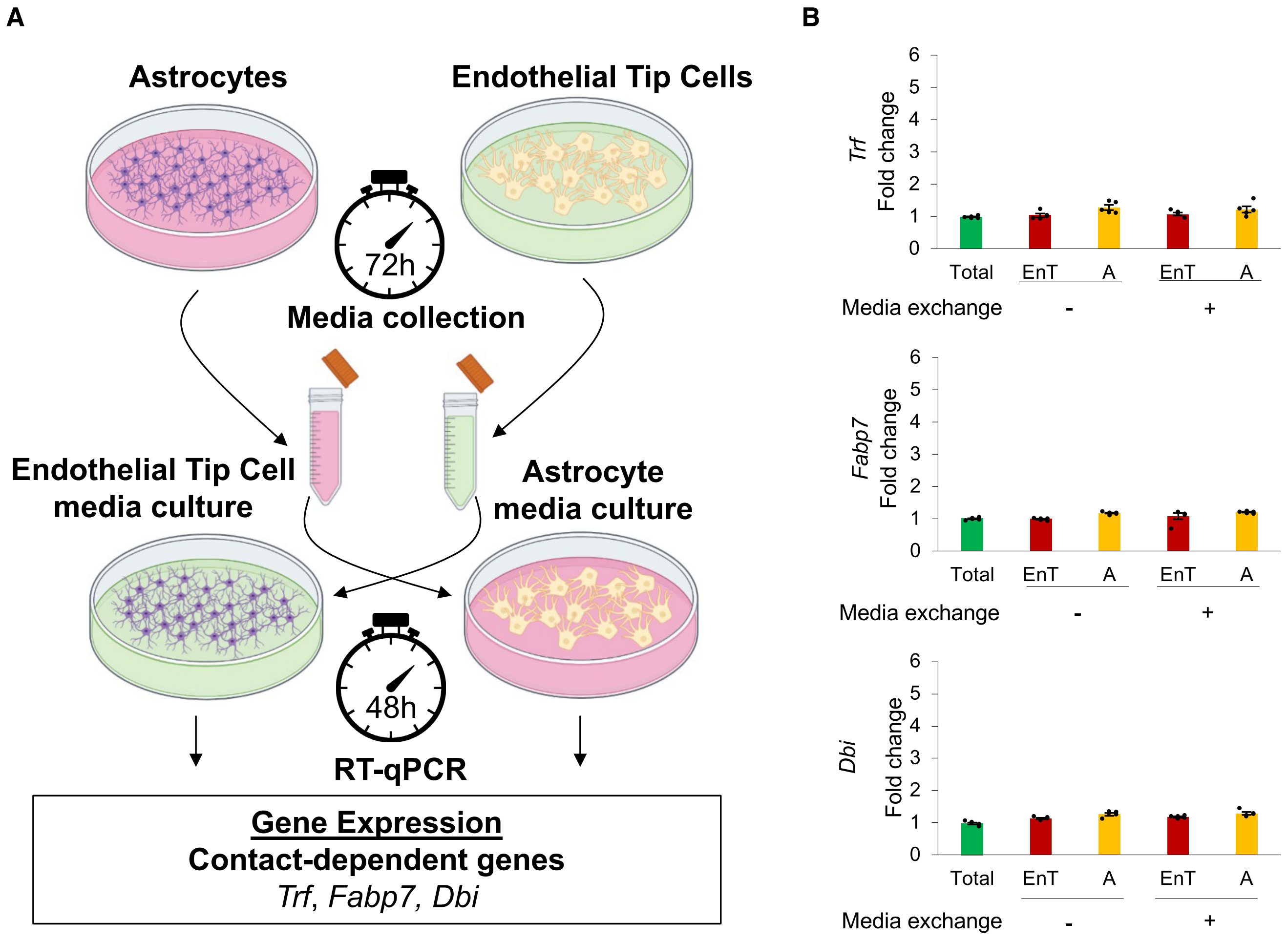

Figure EV4. Validation of neighbor-dependent gene expression upon media exchange of cells derived from mouse hippocampus.

A Model depicting the experimental methodology applied to analyze the expression of the predicted neighbor-dependent genes in the mouse hippocampus upon the exchange of cell-derived medias. Briefly, the isolated Astrocyte and Endothelial Tip (EnT) cells from mouse hippocampus were cultured separately for 72 h. Then, the media derived from Astrocyte was transferred to culture EnT cells, while the media from EnT cells was used to culture Astrocyte for 48 h. The cells were harvested and used to perform RT–qPCR. This model validates the expression of the predicted neighbor-dependent genes *Trf*, *Fabp7*, and *Dbi*.

B The analysis of qPCR-based mRNA expression of Astrocyte (red bars) and EnT cells (yellow bars) upon the media exchange shows no statistically significant differences between the expression of the predicted neighbor-dependent genes in Astrocyte and EnT cells before and after the media exchange. The expression of these genes in total mouse hippocampus represents the control (green bars). The expression of the predicted neighbor-dependent genes was normalized for the expression of these genes in total mouse hippocampus ($N = 5$; bars represent averaged fold changes of gene expression relative to the control; error bars indicate mean ± SE). The expression of all genes follows a normal distribution, which was calculated by the Shapiro–Wilk test and the *F*-test was performed to study the equality of variances. The *P*-values shown in the bar plot were obtained by performing the two-tailed Student's *t*-test with a confidence interval of 95%.

Source data are available online for this figure.

Figure EV5. Heterogeneity of endothelial tip (EnT) cells in mouse hippocampus Slide-seq data.

A Neighboring cell type-dependent gene expression of EnT cells. EnT cells dominantly express *Igfbp7* (red) when proximal to Choroid (EnT + Ch), *Trf* (green) when proximal to Astrocyte (EnT + A), and *Plp1* (blue) when proximal to Interneuron (EnT + In).

B Expression of neighbor-dependent genes in the mouse hippocampus scRNA-seq data. It confirms that the three genes are expressed from EnT.

C UMAP of EnT cells. 4 clusters were obtained through clustering analysis: Cluster 0 to 3. *Igfbp7* is mostly expressed in Cluster 3, *Trf* in Cluster 0, *Plp1* in Cluster 2, and none of them is expressed in Cluster 1.

D Heterogeneity of EnT cells explained by niche-specific gene expression. Cluster 3 is EnT cells adjacent to Ch, Cluster 0 is EnT adjacent to A, Cluster 2 is EnT adjacent to In, and Cluster 0 is EnT adjacent to another EnT.

© 2023 The Authors

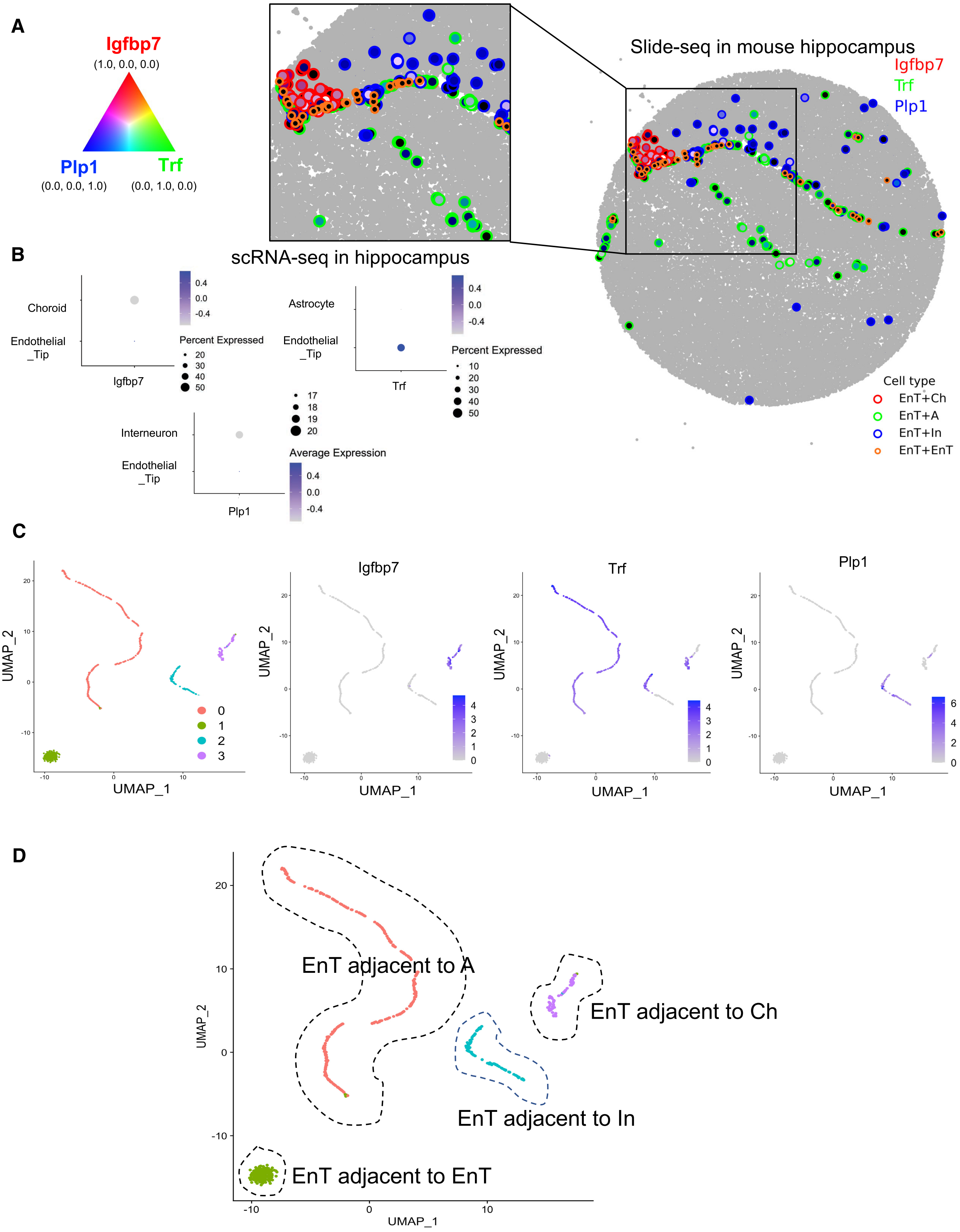

Figure EV5.

 © 2023 The Authors
