# Supplementary material for: CellNeighborEX: deciphering neighbor‐dependent gene expression from spatial transcriptomics data
Source: Mol Syst Biol. 2023 Oct 10;19(11):e11670. doi: 10.15252/msb.202311670 (PMC10632736; doi:10.15252/msb.202311670)
Supplement: Supplementary file 9 — PDF+ [file MSB-19-e11670-s001.pdf]

Method

SOURCE DATA | TRANSPARENT PROCESS | OPEN ACCESS

molecular systems biology

CellNeighborEX: deciphering neighbor-dependent gene expression from spatial transcriptomics data

Hyobin Kim[1,2,†], Amit Kumar[3,4,5,†], Cecilia Lövkvist[6], António M Palma[3,4,7], Patrick Martin[1,2], Junil Kim[8], Praveen Bhoopathi[3,4,5], Jose Trevino[3,9], Paul Fisher[3,4,5], Esha Madan[3,4,5,9], Rajan Gogna[3,4,5,9,*] & Kyoung Jae Won[1,2,**]

Abstract

Cells have evolved their communication methods to sense their microenvironments and send biological signals. In addition to communication using ligands and receptors, cells use diverse channels including gap junctions to communicate with their immediate neighbors. Current approaches, however, cannot effectively capture the influence of various microenvironments. Here, we propose a novel approach to investigate *cell neighbor*-dependent gene *ex*pression (CellNeighborEX) in spatial transcriptomics (ST) data. To categorize cells based on their microenvironment, CellNeighborEX uses direct cell location or the mixture of transcriptome from multiple cells depending on ST technologies. For each cell type, CellNeighborEX identifies diverse gene sets associated with partnering cell types, providing further insight. We found that cells express different genes depending on their neighboring cell types in various tissues including mouse embryos, brain, and liver cancer. Those genes are associated with critical biological processes such as development or metastases. We further validated that gene expression is induced by neighboring partners via spatial visualization. The neighbor-dependent gene expression suggests new potential genes involved in cell–cell interactions beyond what ligand-receptor co-expression can discover.

Keywords cell–cell interactions; cellular communication; neighbor-dependent genes; spatial transcriptomics

Subject Categories Chromatin, Transcription & Genomics; Computational Biology; Methods & Resources

DOI 10.15252/msb.202311670 | Received 23 March 2023 | Revised 14 September 2023 | Accepted 18 September 2023 | Published online 10 October 2023

Mol Syst Biol. (2023) 19: e11670

Introduction

Cells communicate with their microenvironment in various ways including the release of soluble molecules and direct cell contact (Yang *et al*, 2021), actively changing their transcriptomes in response to external signals (Cable *et al*, 2021; Fischer *et al*, 2022). To gain insight into critical biological processes such as diseases and development, it is essential to understand the various ways of cell–cell communication. Experimental approaches to cell–cell communication usually require an elaborate and intricate setup (Nishida-Aoki & Gujral, 2019).

Genome-scale study on cell–cell interactions has been recently possible by using ligand-receptor co-expression on single cell RNA-sequencing (scRNA-seq); Browaeys *et al*, 2020; Efremova *et al*, 2020) and spatial transcriptomics (ST) data (preprint: Pham *et al*, 2020; Garcia-Alonso *et al*, 2021; Li *et al*, 2021; Shao *et al*, 2022). The use of ligand-receptor co-expression enabled inferring interacting cell type pairs and identifying intercellular signaling pathways without relying on a complicated experimental setup. However, it cannot elucidate the gene expression of individual cells changed by direct cell contact.

A growing body of studies on cell communication has demonstrated that cells are influenced by their microenvironment and neighboring cells (Barone *et al*, 2017; Hannezo & Heisenberg, 2019). Grafting experiments in developing embryos manifested that direct cell contact can induce signals for the development of a specific tissue type (Spemann & Mangold, 2003; Solini *et al*, 2017). More recently, RNA sequencing of physically interacting cells (PIC-seq) has revealed that cells express different genes depending on the types of neighboring cells during mouse development (Kim *et al*, 2023). This study suggests that cells have distinct expression

1 Department of Computational Biomedicine, Cedars-Sinai Medical Center, Hollywood, CA, USA
2 Biotech Research and Innovation Centre (BRIC), University of Copenhagen, Copenhagen, Denmark
3 Massey Cancer Center, Virginia Commonwealth University, Richmond, VA, USA
4 School of Medicine, Institute of Molecular Medicine, Virginia Commonwealth University, Richmond, VA, USA
5 Department of Human and Molecular Genetics, School of Medicine, Virginia Commonwealth University, Richmond, VA, USA
6 Novo Nordisk Foundation Center for Stem Cell Medicine, reNEW, University of Copenhagen, Copenhagen, Denmark
7 Instituto Superior Tecnico, Universidade de Lisboa, Lisboa, Portugal
8 School of Systems Biomedical Science, Soongsil University, Seoul, Korea
9 Department of Surgery, School of Medicine, Virginia Commonwealth University, Richmond, VA, USA
*Corresponding author. Tel: +16282526501; E-mail: rajangogna@gmail.com
**Corresponding author. Tel: +13104233967; E-mail: kyoungjae.won@cshs.org
[†]These authors contributed equally to this work

© 2023 The Authors. Published under the terms of the CC BY 4.0 license

profiles through direct cell contact independently from ligand-receptor-mediated communication.

Recent development in ST has opened potential ways to explore the role of the microenvironment. The spatial gene expression profile has made it possible to study the transcriptional activity of a cell together with that of the neighborhood within intact tissues. There are largely two types of ST data with their own advantages and limitations. Image-based approaches including MERFISH (Chen *et al*, 2015) and seqFISH (Lubeck *et al*, 2014; Eng *et al*, 2019) use fluorescence *in situ* hybridization (FISH) to visualize RNA species of interest. While image-based ST approaches can quantify RNAs at cellular resolution, the number of detectable RNA species is still limited. The next-generation-sequencing (NGS)-based ST approaches such as Visium (Ståhl *et al*, 2016) and Slide-seq (Rodriques *et al*, 2019; Stickels *et al*, 2021a; Zhao *et al*, 2022a) leverage spatially barcoded beads. While NGS-based approaches can unbiasedly profile the transcriptome, a barcode can be linked to the mixture of transcriptome of multiple cells or cell portions depending on the position and the resolution of the barcoded spots, making it hard to detect gene expression changed by the cellular microenvironment.

Many computational tools have been developed to understand cell–cell interactions from ST data. CellphoneDB v.3.0 (Garcia-Alonso *et al*, 2021), MESSI (Li *et al*, 2021), SpaTalk (Shao *et al*, 2022), and stLearn (preprint: Pham *et al*, 2020) use the co-expression of ligand-receptor pairs to study cellular communication. However, ligand-receptor co-expression cannot completely capture cell–cell interactions due to direct contact. SVCA (Arnol *et al*, 2019) decomposes the sources of gene expression variation into intrinsic effects, environmental effects, and cell–cell interactions. It explains the relationship between gene expression and cell–cell interactions. However, SVCA does not have a function to detect gene expression change associated with cell contact, and their strategy has been only optimized for image-based ST data. As MISTy (Tanevski *et al*, 2022) quantifies the contributions of different spatial contexts to the expression of markers of interest, the influence of immediate neighborhoods on the expression of markers can be investigated. However, MISTy requires to pre-select the list of marker genes to find potential interactions and it has not been designed to identify gene expression change related to cell contact in an unbiased way. DeepLinc (Li & Yang, 2022) reconstructs a cell interaction network from ST data. Regarding three nearest neighbors as direct contact, DeepLinc finds signature genes contributing to interactions between cell types and infers proximal interactions between them. However, it does not uncover specific relationships between the signature genes and interacting cell types. C-SIDE (Cable *et al*, 2022) examines up- and down-regulated genes depending on proximity to a certain cell type. Because the interaction between cell types is defined based on cell density rather than cell contact, C-SIDE does not work for studying cell contact-dependent gene expression. NCEM (Fischer *et al*, 2022) investigates transcriptomic change depending on local environments but it has not been designed to study the influence of cell contact on gene expression, particularly for NGS-based data. NCEM considers one barcoded-spot a single cell type even in low resolution Visium data, so it does not look into the influence of direct contact between multiple cell types within one spot. Although spatial context has been applied to study cell–cell interactions, transcriptomic change associated with cell contact has not been fully explored yet. It is still challenging to detect genes influenced by cell contact regardless of the data types.

Here, we propose a universal approach called CellNeighborEX to identify genes influenced by neighboring cells in ST data. CellNeighborEX dissects the transcriptome of cells with their immediate neighbors to categorize cells based on the neighboring cell types. For NGS-based ST data where exact cell locations are not available, CellNeighborEX actively uses the mixture of transcriptome to identify immediate neighbors. CellNeighborEX has been applied to various ST data from mouse embryos, hippocampus, and liver cancer to identify neighbor-dependent genes. These transcriptomic changes have been confirmed in the spatial context. We showed that cells express specific genes depending on their neighboring cell types. The neighbor-dependent gene expression suggests new potential genes involved in cell–cell interactions beyond what ligand-receptor co-expression can discover and gives clues on complex biological processes.

Results

CellNeighborEX categorizes transcriptome to investigate the influence of neighboring cell types

CellNeighborEX defines immediate neighbors differently for image- and NGS-based ST data. In image-based ST data where exact cell locations are available, CellNeighborEX finds the nearest neighbors using Delaunay triangulation (Delaunay, 1934), radial distance, or k-nearest neighbors (KNN; Fix & Hodges, 1951; Cover & Hart, 1967; Fig 1A). Both radial distance and KNN require prior knowledge. KNN is useful for cells evenly distributed on a tissue. Radial distance is useful when we know the distance between the cells of interest. Delaunay triangulation can be used alone or in combination with radial distance.

CellNeighborEX classifies all cells into two groups based on the cell types of the nearest neighbors. *Homotypic neighbors* consist of the same cell type. *Heterotypic neighbors* are composed of different cell types. The influence of neighboring cells can be measured by comparing the transcriptome of heterotypic neighbors against that of homotypic neighbors (log ratio > 0.4, P-value < 0.01, FDR < 0.05). Parametric (*i.e.*, Student's t-test, Welch's t-test) or non-parametric (*i.e.*, Mann–Whitney U test) statistical tests are performed as differential expression (DE) analysis depending on the sample size of the groups (see Materials and Methods). This strategy also works for the NGS-based ST approach with high resolution (< 1 μm resolution) after cell segmentation.

The locations of cells are not explicitly given for NGS-based ST approaches and a barcoded spot contains the mixed transcriptome from multiple cells. CellNeighborEX capitalizes on spots with multiple cell types (or heterotypic spots) as the evidence for cell contact (Fig 1B). This strategy is effective in studying neighbor-dependent gene expression when the diameter of a spot is near cellular resolution. For instance, Slide-seq, a ST approach with 10 μm resolution, has *homotypic spots* (the same cell type in a bead) and *heterotypic spots* (two or more cell types in a bead), and the majority of the heterotypic spots are composed of two cell types (97% of the spots are composed of one or two cell types) (Rodriques *et al*, 2019). CellNeighborEX uses RCTD (Cable *et al*, 2021) to decompose the cell

 © 2023 The Authors

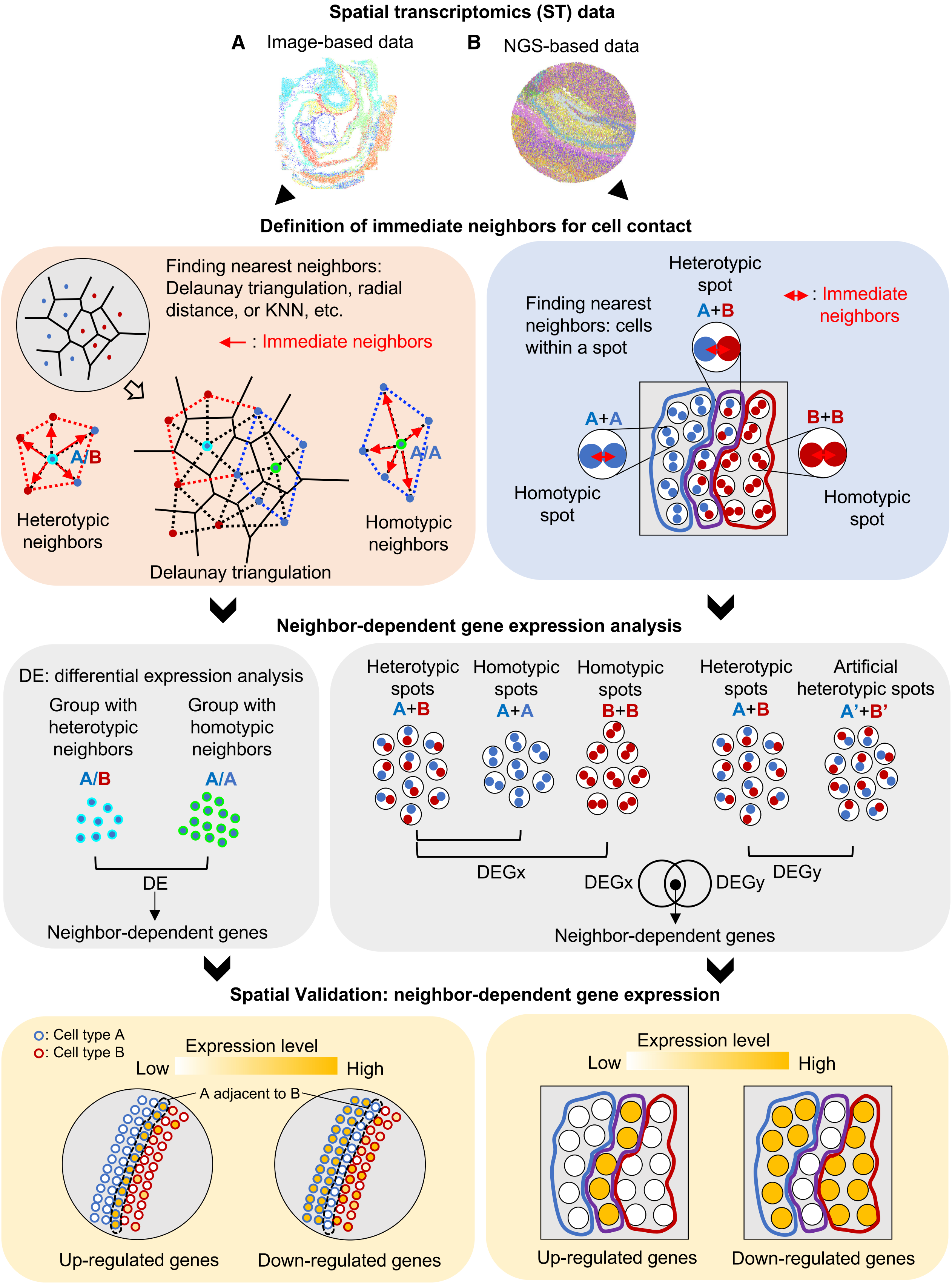
Spatial transcriptomics (ST) data
A Image-based data
B NGS-based data
Definition of immediate neighbors for cell contact
Finding nearest neighbors: Delaunay triangulation, radial distance, or KNN, etc.
: Immediate neighbors
A/B
Heterotypic neighbors
Delaunay triangulation
A/A
Homotypic neighbors
Finding nearest neighbors: cells within a spot
Heterotypic spot
A+B
: Immediate neighbors
A+A
Homotypic spot
B+B
Homotypic spot
Neighbor-dependent gene expression analysis
DE: differential expression analysis
Group with heterotypic neighbors
Group with homotypic neighbors
A/B
A/A
DE
Neighbor-dependent genes
Heterotypic spots
A+B
Homotypic spots
A+A
Homotypic spots
B+B
Heterotypic spots
A+B
Artificial heterotypic spots
A'+B'
DEGx
DEGx
DEGy
DEGy
Neighbor-dependent genes
Spatial Validation: neighbor-dependent gene expression
: Cell type A
: Cell type B
Expression level
Low
High
A adjacent to B
Up-regulated genes
Down-regulated genes
Expression level
Low
High
Up-regulated genes
Down-regulated genes

Figure 1.

Figure 1. Workflow of CellNeighborEX.

A In image-based ST data, immediate neighbors for cell contact are determined by algorithms such as Delaunay triangulation, radial distance, and KNN. Based on their cell types, homotypic and heterotypic neighbors are defined. CellNeighborEX detects genes influenced by neighbors by comparing the transcriptome of heterotypic neighbors with that of homotypic neighbors.

B In NGS-based ST data, there are homotypic spots (the same cell type in a bead) and heterotypic spots (multiple cell types in a bead). The heterotypic spots are regarded as evidence for cell contact. CellNeighborEX compares the heterotypic spots with the homotypic ones to detect neighbor-dependent genes. Additional statistical tests with null models are applied for validation.

types of spots in Slide-seq data. RCTD also estimates the cell type proportions for each spot in ST data based on scRNA-seq data. The transcriptome of heterotypic spots is compared with that of homotypic spots to identify neighbor-dependent genes (log ratio > 0.4, *P*-value < 0.01). To further confirm the statistical significance of the identified neighbor-dependent genes, CellNeighborEX generates a null model by creating artificial heterotypic spots (see Materials and Methods—Appendix Fig S1). The artificial heterotypic spots are created by merging two random homotypic spots using information on the heterotypic spots' cell type proportions estimated by RCTD. The artificial heterotypic spots represent two different cell types just combined without cell–cell interactions. We confirmed that our neighbor-dependent genes were also differentially expressed against these artificial spots (FDR < 0.01).

CellNeighborEX identifies neighbor-dependent genes related to embryonic development in seqFISH data from a mouse embryo

We applied CellNeighborEX to seqFISH data from a mouse embryo. We used the seqFISH data pre-processed by Squidpy (Palla *et al*, 2022a; Data ref: Palla *et al*, 2022b). After defining immediate neighbors using Delaunay triangulation (Delaunay, 1934), CellNeighborEX categorized cells based on neighboring cell types. After the DE analysis, CellNeighborEX detected 354 up-regulated genes from 22 types of heterotypic neighbors and 429 down-regulated genes from 22 types of heterotypic neighbors (Dataset EV1A and B).

For instance, we found 694 Gut tube cells surrounded by the same cell type (homotypic neighbors) and 33 Gut tube cells adjacent to Neural crest cells among heterotypic neighbors. CellNeighborEX found that Gut tube cells express *Pitx1* when adjacent to Neural crest cells (*P*-value < 0.01 and FDR < 0.05; Fig EV1A). To confirm this result, we visualized the expression of *Pitx1* for the Gut tube cells together with the Neural crest cells. The Gut tube cells adjacent to Neural crest (Gut tube/Neural crest) are represented in red boundaries, while other Gut tube cells (Gut tube/Gut tube) are represented in blue boundaries. The Neural crest cells are shown in black boundaries. The rest of the cells are shown in gray (Fig EV1B). The visualization demonstrated the clear expression of *Pitx1* in the Gut tube cells adjacent to Neural crest (Gut tube/Neural crest) but not in the Gut tube cells adjacent to other Gut tube cells (Gut tube/Gut tube; Fig EV1B). It was previously observed that cells are differentiated from neural cells through stomodeum to gut cells and *Pitx1* is highly expressed during the differentiation process (Lanctôt *et al*, 1997; Chung *et al*, 2010; Tran & Kioussi, 2021).

For the 354 up-regulated genes, we carried out Gene Ontology (GO) analysis using the enrichGO function from the clusterProfiler R package. We selected top 20 biological process GO terms based on the adjusted *P*-values. Here, the *P*-value represents the probability of observing the association between the set of genes and the GO terms by chance. We found that the top 20 GO terms were associated with biological terms such as "pattern specification process" and "embryonic organ morphogenesis" (Fig EV1C), suggesting that cell contact with other cells- or tissue types may regulate the coordinated developmental processes.

CellNeighborEX detects neighbor-dependent genes in slide-seq data from a mouse embryo

We analyzed Slide-seq V2 data from a mouse embryo. For this NGS-based data, CellNeighborEX uses heterotypic beads to define immediate neighbors. To find heterotypic spots, we applied the RCTD (Cable *et al*, 2021) deconvolution tool using single cell RNA-sequencing (scRNA-seq) data from a mouse embryo (Cao *et al*, 2019a; Data ref: Cao *et al*, 2019b) as a reference. As a result, RCTD identified 8,094 homotypic and 34,268 heterotypic spots in the mouse embryo. The decomposed cell types of the heterotypic spots were additionally validated by the expression of cell type markers and correlation analysis (see Materials and Methods). We confirmed whether the respective cell type markers are expressed in the annotated heterotypic spots. We also examined correlation between true heterotypic spots and artificial ones. We generated many combinatorial types of artificial heterotypic spots (Appendix Fig S2). We checked if the cell types of true heterotypic spots are the same as those of artificial heterotypic spots with the largest correlation. For the validated heterotypic spots, comparing the gene expression levels of heterotypic spots with the expression of homotypic spots, CellNeighborEX detected neighbor-dependent genes. Finally, CellNeighborEX found 28 up-regulated genes from 9 heterotypic pairs, and 28 down-regulated genes from 11 heterotypic pairs in the embryo (Dataset EV1C and D).

For example, CellNeighborEX identified 17 genes including *Cd24a*, which are highly expressed in the heterotypic spots of Endothelial and Lens cells compared with their respective homotypic spots (Fig EV2A). The heterotypic spots expressed marker genes in both Endothelial (*Pecam1* and *Egfl7*) and Lens cells (*Cryba1* and *Cryaa*). We confirmed in the spatial visualization that *Cd24a* is more highly expressed in the heterotypic spots of Endothelial+Lens cells (red boundaries) compared with the homotypic spots of Endothelial (blue) and Lens (black) cells (Fig EV2B).

To find from which cell type the expression of *Cd24a* comes between Endothelial cells and Lens cells, we used a regression model in which gene expression is shown along the proportion of one cell type in the heterotypic spots (see Materials and Methods). If gene expression increases as the proportion of one cell type grows, we regarded the cell type as the source of the expression. Otherwise, the other cell type was considered the origin of the expression. We found that the expression level of *Cd24a* increased as the proportion of Endothelial cells (against Lens cells) increased (Appendix Fig S3A). We further confirmed that *Cd24a* is expressed mainly from Endothelial cells when examining scRNA-

 © 2023 The Authors

seq data from a mouse embryo (Cao *et al*, 2019a; Data ref: Cao *et al*, 2019b; Fig EV2C). The *Cd24a* (*CD24*) protein is known as a ligand for P-selectin and a cell adhesion molecule (CAM) involved in cellular binding in Endothelial cells (Sammar *et al*, 1994).

For the 28 up-regulated genes, the GO analysis showed terms related to tissue development including "eye development" and "digestive system development" (Fig EV2D), further suggesting the role of cell contact in the developmental processes.

CellNeighborEX detects genes influenced by the tumor microenvironment (TME) in slide-seq data from mouse liver cancer

For Slide-seq data in mouse liver metastases, RCTD used single-nucleus RNA-sequencing (snRNA-seq) data in mouse liver cancer (Zhao *et al*, 2022a; Data ref: Zhao *et al*, 2022b) as a reference. It identified 16,557 homotypic and 6,284 heterotypic spots. After validating the cell type annotation of heterotypic spots (see Materials and Methods—Appendix Fig S2), CellNeighborEX detected 42 up-regulated genes from 10 heterotypic pairs and 3 down-regulated genes from 2 heterotypic pairs (Dataset EV1E and F).

For instance, CellNeighborEX found that *F13a1* is highly expressed when Monocyte cells contact Tumor cells (Fig EV3A). Its spatial mapping displays the higher expression in the heterotypic spots than the homotypic ones (Fig EV3B). Using the regression model and snRNA-seq data, we checked that the expression of *F13a1* is derived from Monocyte cells (Appendix Fig S3B and Fig EV3C). Previous global proteomic analysis on small extracellular vesicles identified that *F13a1* is associated with liver cancers (Dong *et al*, 2022). Besides, *F13a1* promotes lung squamous cancer (Porrello *et al*, 2018) and is a biomarker for colorectal cancers (Peltier *et al*, 2018).

For the 42 up-regulated genes, the GO analysis showed terms such as "collagen fibril organization" and "endothelial cell migration" (Fig EV3D). Specifically, *Col1a2*, *Col3a1*, *Ext1*, and *Tgfbr1* are associated with collagen fibril organization. *Col1a2* and *Col3a1* are more expressed when Vascular smooth muscle cells contact Tumor III cells (VSMC+Tumor III). *Ext1* and *Tgfbr1* are more expressed when Tumor III cells contact Hepatocyte II cells (Tumor III + Hepatocyte II). These four genes are all highly expressed when non-tumor and tumor cells interact with each other. Based on previous studies showing that collagen influences cancer cell behaviors such as metastasis, tumorigenesis, and proliferation (Xu *et al*, 2019), we can infer that the four genes might affect liver cancer by controlling the collagen arrangement among VSMC, Hepatocyte, and Tumor cells. A vitro study actually showed that the down-regulation of *Col1a2* suppressed hepatocellular cancer (Ji *et al*, 2010), which supports that neighbor-dependent genes are indeed associated with critical biological processes. It is notable that CellNeighborEX can detect genes influenced by TME in an unbiased way.

CellNeighborEX discovers neighbor-dependent genes in slide-seq data from mouse brain

We also analyzed Slide-seq V2 data from mouse brain. Using scRNA-seq data for mouse hippocampus (Saunders *et al*, 2018a; Data ref: Saunders *et al*, 2018b) as a reference, we ran RCTD (Cable *et al*, 2021). In total, 12,013 homotypic and 29,331 heterotypic spots were identified in the mouse hippocampus. We additionally validated the decomposed cell types of the heterotypic spots through the expression of cell type markers and correlation analysis (see Materials and Methods—Appendix Fig S2). CellNeighborEX found 155 up-regulated genes from 21 heterotypic pairs and 55 down-regulated genes from 8 heterotypic pairs for this dataset (Dataset EV1G and H).

For example, CellNeighborEX detected 3 up-regulated genes in the heterotypic spots of Endothelial tip (EnT) and Astrocyte cells including *Fabp7* (Fig 2A). Its spatial visualization shows that *Fabp7* is more expressed in the heterotypic spots than the respective homotypic ones (Fig 2B). Using the scRNA-seq data from hippocampus (Saunders *et al*, 2018a; Data ref: Saunders *et al*, 2018b), we examined the potential cell types expressing these genes (Fig 2C). For the 155 up-regulated genes, the GO analysis showed terms such as "regulation of metal ion transport" and "dopamine secretion", suggesting that cell contact may contribute to neuronal processes in the mouse brain (Fig 2D).

To validate the accuracy of our findings, we designed a set of experiments to observe the expression of the neighbor-dependent genes, *Trf*, *Fabp7*, and *Dbi*, which were shown to be upregulated in our computational predictions when Astrocyte are adjacent to EnT cells. We isolated Astrocyte and EnT cells from mouse hippocampus as demonstrated graphically by Fig 3A. This strategy leverages specific cell membrane markers such as CD34-positive, VEGFR2-positive, and VEGFR1-negative for EnT cells and ACSA2-positive and CD11b-negative for Astrocyte (Fig 3B). We used two different approaches to analyze the expression of *Trf*, *Fabp7*, and *Dbi* via qPCR-based mRNA expression analysis of Astrocyte and EnT cells isolated from the mouse hippocampus. In our first approach, we labeled Astrocyte with GFP using comet-pD2109-CMV lentiviral particles. Next, we used monocultures of EnT cells, Astrocyte GFP-positive, and 48 h co-cultures of these two cell types in a 1:1 ratio. After sorting Astrocyte and EnT cells based on their GFP expression (Fig 3C), we used monocultured and co-cultured Astrocyte and EnT cells to determine the expression of *Trf*, *Fabp7*, and *Dbi*. Through this experimental approach, we observed that the expression of *Trf*

Figure 2. Neighbor dependent-genes identified by CellNeighborEX in mouse hippocampus Slide-seq data.

A The heterotypic spots of Endothelial tip and Astrocyte cells (EnT + A) ($n = 121$) were compared with the respective homotypic spots (EnT [$n = 60$], Astrocyte [$n = 2,909$]; log ratio > 0.4, P-value < 0.01) and additionally with the artificial heterotypic spots (FDR < 0.01). The statistical test was chosen among the two tailed Student's t-test, Welch's t-test, or Wilcoxon rank sum test depending on the sample size and heterogeneity of variance test. The volcano plot displays 3 up-regulated genes including *Fabp7*. In the heatmap, the genes are more highly expressed in EnT + A. The heterotypic spots also express both EnT and Astrocyte markers.

B The spatial visualization shows the higher expression level of *Fabp7* in EnT + A.

C In the mouse hippocampus scRNA-seq data, *Fabp7* is mostly expressed in Astrocyte.

D In GO analysis, the GO terms are related to neuronal regulation. The bar plot presents top 20 GO terms.

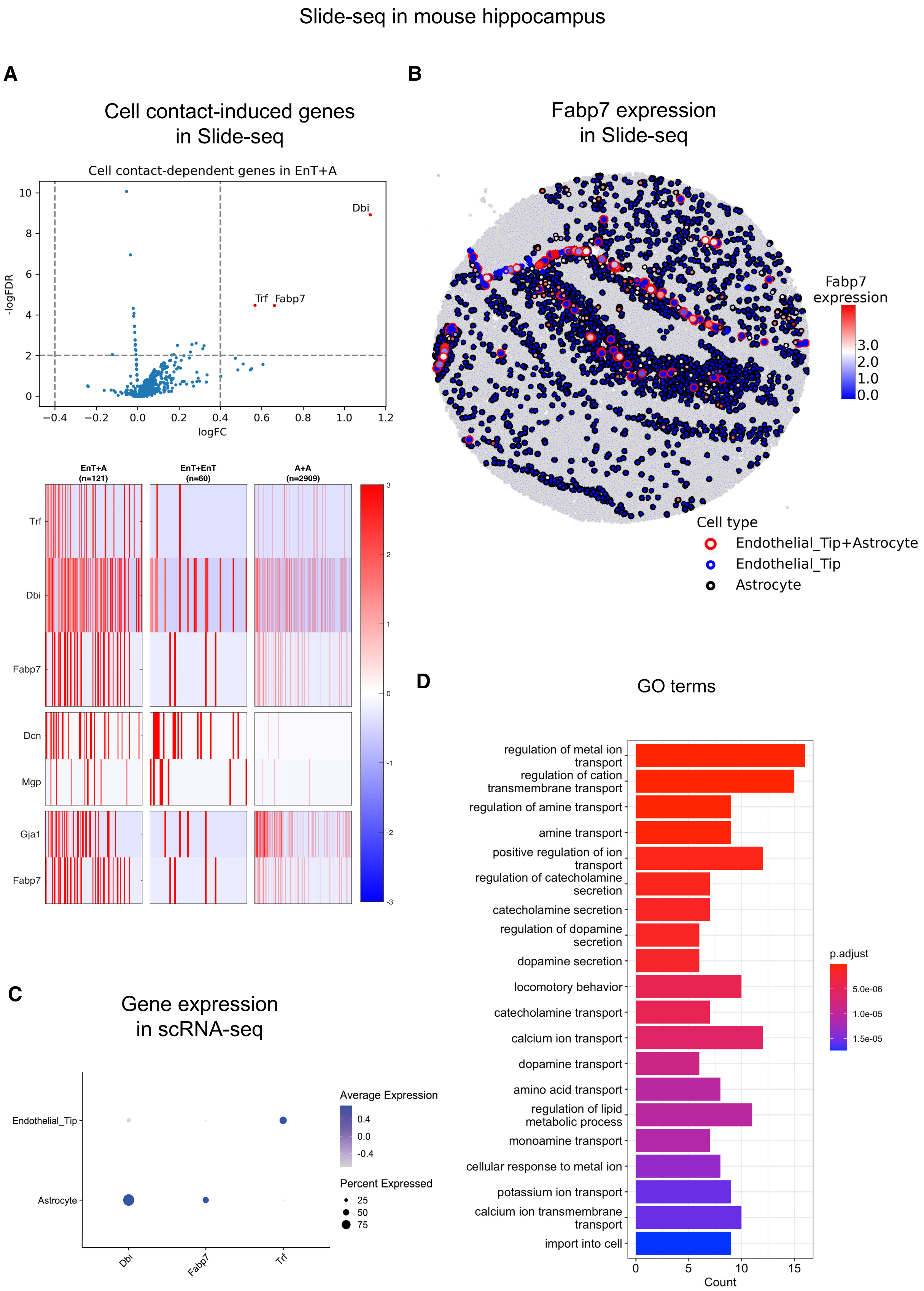

Figure 2.

 © 2023 The Authors

is up-regulated by approximately 4-fold only in EnT cells co-cultured with Astrocyte (Fig 3D). We also observed that both *Fabp7* and *Dbi* have an approximately 5-fold increased expression only in Astrocyte co-cultured with EnT cells (Fig 3D).

To show that the expression of our tested genes was due to not the release of cellular factors but direct cell contact, we performed a media exchange assay in which we cultured EnT cells with Astrocyte-derived media, and cultured Astrocyte with EnT

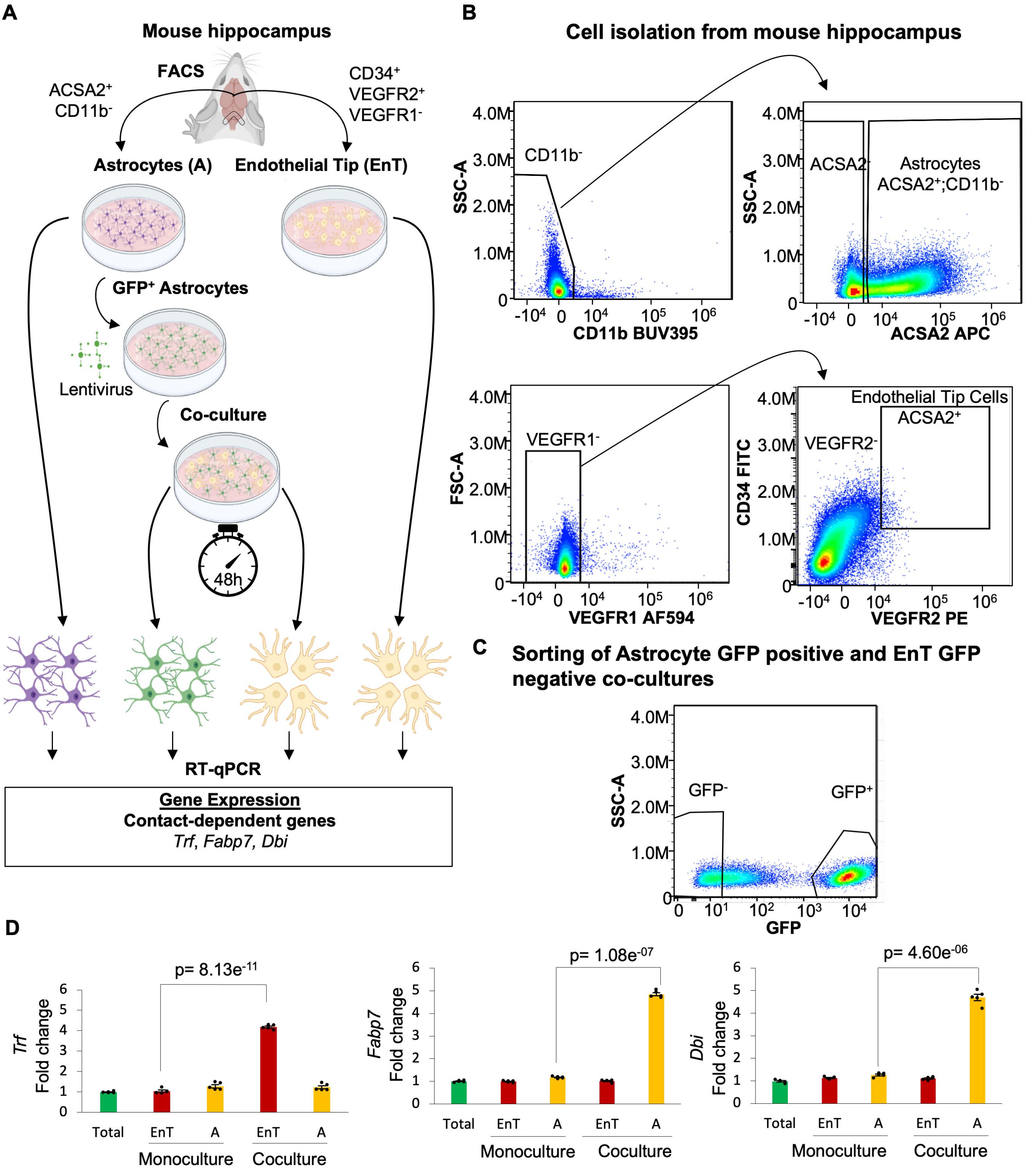

Figure 3.

Figure 3. Validation of neighbor-dependent gene expression predictions upon co-culture of cells derived from mouse hippocampus.

A Model depicting the experimental methodology applied to analyze the expression of the predicted neighbor-dependent genes in the mouse hippocampus. Briefly, we isolated Astrocyte and EnT cells from mouse hippocampus through cell sorting with specific cell membrane markers for these cell types. Then, we labeled Astrocyte with GFP and performed monocultures of Astrocyte and EnT as well as co-culture of both cell types for 48 h, followed by sorting of Astrocyte and EnT cells based on their GFP expression. The sorted cells and the monocultures were used to perform RT–qPCR to determine the expression of the predicted neighbor-dependent genes *Trf*, *Fabp7*, and *Dbi*.

B FACS of Astrocyte and EnT cells from mouse hippocampus. The top-left gate shows the selection of CD11b negative cells, which were used to further select those with a positive expression of ACSA2 (top-right gate), which represent the Astrocyte population. Additionally, we selected VEGFR1 negative cells (bottom-left gate) to sort those that were double positive for VEGFR2 and CD34 as our EnT cell population.

C Sorting of Astrocyte and EnT cells based on GFP expression upon Astrocyte GFP positive and EnT GFP negative co-cultures.

D The analysis of qPCR-based mRNA expression of Astrocyte and EnT cells coculture shows that, upon co-culture, the upregulation of *Trf* is specific for EnT cells (red bars, $P = 8.13E-11$), while the expression of *Fabp7* ($P = 1.08E-07$) and *Dbi* ($P = 4.6E-06$) is specific for Astrocyte (yellow bars). The expression of these genes in total mouse hippocampus represents the control (green bars). The expression of the predicted neighbor-dependent genes was normalized for the expression of these genes in total mouse hippocampus ($N = 5$; bars represent averaged fold changes of gene expression relative to the control; error bars indicate mean ± SE). The *P*-values shown in the bar plot were obtained by performing the two-tailed Student's *t*-test. The expression of all genes follows a normal distribution, which was calculated by the Shapiro–Wilk test and the *F*-test was performed to study the equality of variances. The *P*-value shown in the bar plot for the expression of *Trf* was obtained by performing the two-tailed Student's *t*-test, whereas the *P*-values obtained for *Fabp7* and *Dbi* were calculated by the two-tailed Welch's *t*-test with a confidence interval of 95%.

Source data are available online for this figure.

cells-derived media for 48 h (Fig EV4A). Then, we analyzed the expression of *Trf*, *Fabp7*, and *Dbi*. In these conditions, we did not observe significant changes in their expression of these genes compared to their expression in the mouse hippocampus when Astrocyte and EnT cells were cultured in their respective media (Fig EV4B). These results suggest that the induction of *de novo* gene expression results from the communication established by EnT and Astrocyte.

Neighbor-dependent genes are new potential genes involved in cell–cell interactions

Ligand-receptor co-expression has been used to study cell–cell interactions in ST data (preprint: Pham *et al*, 2020; Garcia-Alonso *et al*, 2021; Li *et al*, 2021; Shao *et al*, 2022). To see if the use of ligand-receptor pairs and the downstream genes mediated by them can recover our genes detected by CellNeighborEX, we ran NicheNet (Browaeys *et al*, 2020) on the seqFISH and Slide-seq datasets (see Materials and Methods). NicheNet detected ligand-receptor pairs and their target genes on each dataset based on prior knowledge of signaling and gene regulatory networks. We found 174, 1, 12, and 11 genes commonly detected between the two approaches, respectively (Appendix Fig S4 and Dataset EV2). To see if the interactions are valid, we calculated minimum distances between the two interacting cell types using the spatial coordinates of the datasets, and obtained its distribution. The estimated distances ranged from 60 to 1,600 μm on average (Appendix Fig S5). This suggests that the interactions identified by NicheNet may include many false predictions. NicheNet only finds frequently interacting cell types based on the averaged gene expression and it does not examine interactions between individual cells.

To demonstrate the usefulness of using the heterotypic spots in Slide-seq, we additionally ran NicheNet on the heterotypic spots. We set the heterotypic spots as a receiver as well as a sender (autocrine mode in NicheNet). We found that 2 genes (of 28 up-regulated genes from 9 heterotypic pairs) in the embryo, 15 genes (of 42 up-regulated genes from 10 heterotypic pairs) in the liver cancer, and additional 11 genes (of 155 up-regulated genes from 21 heterotypic pairs) in the hippocampus common to our neighbor-dependent genes (Dataset EV3), suggesting that the use of heterotypic beads are useful in identifying genes related to cell communication.

Neighbor-dependent genes demonstrate niche-specific expression

We further examined if neighbor-dependent genes are able to show that cells express specific sets of genes depending on their niches. By running CellNeighborEX on the mouse embryo seqFISH data (Fig 4A), we found that Gut tube cells highly express *Tbx1* when adjacent to Cranial mesoderm, *Pitx1* when adjacent to Neural crest, and *Foxf1* when adjacent to Splanchnic mesoderm in the mouse embryo (Fig 4B). To investigate niche-specific gene expression, we colored the boundary of Gut tube cells based on its neighboring cell types: red when proximal to Cranial mesoderm, green when to Neural crest, blue when to Splanchnic mesoderm, and orange when to another Gut tube. To easily distinguish gene expression change depending on neighboring cell types, we defined the neighboring cell type-specific genes using RGB color channels (see Materials and Methods): *Tbx1* (red), *Pitx1* (green), and *Foxf1* (blue). Then, we represented the expression of these three genes using the combination of each color channel. Among the three genes, *Tbx1* (red inside the boundary) is dominantly expressed when Gut tube cells are next to Cranial mesoderm (Gut-tube/Cranial mesoderm, red boundary), *Pitx1* (green inside the boundary) is dominantly expressed when Gut tube cells are next to Neural crest (Gut-tube/Neural crest, green boundary), and *Foxf1* (blue inside the boundary) is dominantly expressed when Gut tube cells are next to Splanchnic mesoderm (Gut-tube/Splanchnic-mesoderm; Fig 4B). These results indicate that Gut tube cells vary the expression levels of these genes depending on their neighboring cell types.

Figure 4. Niche-specific gene expression in seqFISH.

A The cells of the seqFISH data consist of 21 cell types except for Low quality (*i.e.*, unidentified cells due to low quality).

B The spatial mapping with RGB channels simultaneously visualizes the expression of three neighbor-dependent genes. Gut tube cells express *Tbx1* (red) when adjacent to Cranial mesoderm, *Pitx1* (green) when adjacent to Neural crest, and *Foxf1* (blue) when adjacent to Splanchnic mesoderm.

© 2023 The Authors

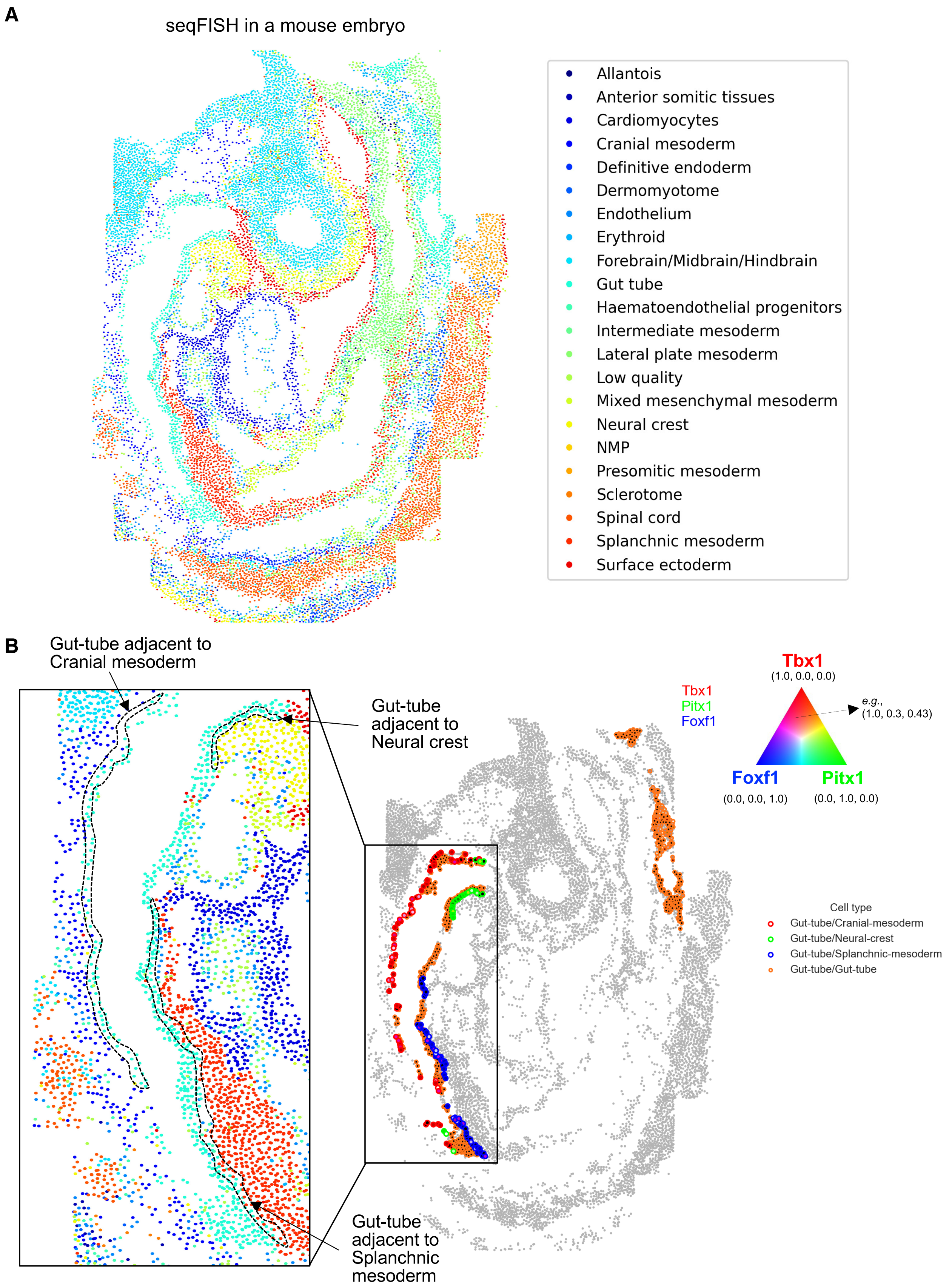
A
seqFISH in a mouse embryo
Allantois
Anterior somitic tissues
Cardiomyocytes
Cranial mesoderm
Definitive endoderm
Dermomyotome
Endothelium
Erythroid
Forebrain/Midbrain/Hindbrain
Gut tube
Haematoendothelial progenitors
Intermediate mesoderm
Lateral plate mesoderm
Low quality
Mixed mesenchymal mesoderm
Neural crest
NMP
Presomitic mesoderm
Sclerotome
Spinal cord
Splanchnic mesoderm
Surface ectoderm
B
Gut-tube adjacent to Cranial mesoderm
Gut-tube adjacent to Neural crest
Gut-tube adjacent to Splanchnic mesoderm
Tbx1
Pitx1
Foxf1
Tbx1
(1.0, 0.0, 0.0)
e.g.,
(1.0, 0.3, 0.43)
Foxf1
(0.0, 0.0, 1.0)
Pitx1
(0.0, 1.0, 0.0)
Cell type
Gut-tube/Cranial-mesoderm
Gut-tube/Neural-crest
Gut-tube/Splanchnic-mesoderm
Gut-tube/Gut-tube

Figure 4.

In the Slide-seq data, we also observed similar niche-specific expression changes: in the embryo (Appendix Fig S6A), *Hba-a1* (red) and *Cdkn1c* (green) are primarily expressed in Primitive erythroid lineage when contacting Definitive erythroid lineage (PEL + DEL) and Limb mesenchyme (PEL + LM), respectively (Fig 5A). A regression study and investigation of scRNA-seq further confirmed that PEL cells mostly express these two genes (Cao *et al*, 2019a; Data ref: Cao *et al*, 2019b; Appendix Fig S3C and D, and Fig 5B). In the hippocampus (Appendix Fig S6B), *Nnat* (red), *Gda* (green), and *Atp2b1* (blue) are mostly expressed in Entorhinal cells when contacting Choroid (Ento+Ch), Neuron.Slc17a6 (Ento+N), and Interneuron (Ento+In), respectively (Fig 5C). We confirmed that the expression of the three genes was derived from Ento in the hippocampus scRNA-seq data (Saunders *et al*, 2018a; Data ref: Saunders *et al*, 2018b; Fig 5D). In the liver cancer (Appendix Fig S6C), *Marco* (red), *Vti1a* (green), *F13a1* (blue) are largely expressed in Monocyte cells when contacting Hepatocyte I (Monocyte+Hepatocyte I), Hepatocyte II (Monocyte+Hepatocyte II), and Tumor III (Monocyte+Tumor III), respectively (Fig 5E). Through the regression models and snRNA-seq data in the liver cancer, we confirmed that Monocyte cells dominantly express the three genes (Appendix Fig S3B, E, and F, and Fig 5F). Our results indicate that cells actively respond to their microenvironment and communicate with their neighboring cells.

Niche specific-gene expression accounts for cellular heterogeneity

Cellular heterogeneity is caused by a number of reasons in different contexts. scRNA-seq is useful in describing cell heterogeneity but cannot explain the cause of heterogeneity. We tested if the niche specific genes obtained from ST study can account for cellular heterogeneity in scRNA-seq. We selected a cell type showing neighbor-dependent gene expression. Figure EV5A shows an example of neighboring cell type-dependent gene expression in the hippocampus Slide-seq V2 data. Endothelial tip cells dominantly express *Igfbp7* (red) when proximal to Choroid (EnT + Ch), *Trf* (green) when proximal to Astrocyte (EnT + A), and *Plp1* (blue) when proximal to Interneuron (EnT + In). In the hippocampus scRNA-seq data, it was confirmed that the three genes were mostly expressed from EnT (Fig EV5B).

To investigate the heterogeneity of EnT cells, we separately selected EnT cells in the scRNA-seq data. For the EnT cells, we performed clustering analysis using the expression values of the three genes and identified 4 clusters (Fig EV5C). The UMAP plot shows the expression of *Igfbp7*, *Trf*, and *Plp1* in the corresponding clusters. We further labeled them based on the neighboring cell type information (Fig EV5D). We also found an additional example for the heterogeneity of Interneuron cells in the hippocampus Slide-seq V2 data (Appendix Fig S7). These findings suggest a possibility that cellular heterogeneity might be stemmed from neighboring cell type-dependent gene expression.

Discussion

Cell communication is a fundamental process related to various functions such as cell growth, development, and diseases (Yang *et al*, 2021). Cell communication coordinates the functions of multicellular organisms (Radhakrishnan *et al*, 2010). Even with its importance, a systematic study of cell communication was not easy without a well-curated experimental setup until scRNA-seq is available. To study cell–cell interactions in scRNA-seq data, co-expression of ligand-receptor pairs has been used (Browaeys *et al*, 2020; Efremova *et al*, 2020). However, it was not possible to study other types of cell communication such as direct contact due to the loss of spatial information in scRNA-seq.

RNA sequencing of physically interacting multi-cells or PIC-seq has provided the transcriptomic landscapes of cells contacting each other (Boisset *et al*, 2018; Giladi *et al*, 2020; Kim *et al*, 2023). In our previous study using PIC-seq from mouse embryos, we found that direct cell contact can induce the expression of specific gene sets depending on the neighboring cell types (Kim *et al*, 2023). This unbiased approach systematically studied cell contact-dependent expression during mouse development. However, PIC-seq can be biased to cell interactions more strongly bound to each other.

ST technologies have enabled spatial mapping of gene expression and have provided opportunities to look into cellular microenvironments. Accordingly, a number of computational methods that investigate cell–cell interactions in the spatial domain have been developed (Arnol *et al*, 2019; preprint: Pham *et al*, 2020; Garcia-Alonso *et al*, 2021; Li *et al*, 2021; Cable *et al*, 2022; Fischer *et al*, 2022; Li & Yang, 2022; Shao *et al*, 2022; Tanevski *et al*, 2022). However, cell communication through direct cell contact has not been thoroughly explored yet. Still, most methods focus on finding frequently interacting cell type pairs based on ligand-receptor co-expression (preprint: Pham *et al*, 2020; Garcia-Alonso *et al*, 2021; Li *et al*, 2021; Shao *et al*, 2022). Here, we presented CellNeighborEX to analyze the influence of direct cell contact on the transcriptome of cells in ST data. CellNeighborEX was designed to work for both image- and NGS-based ST data by defining immediate neighbors differently (Fig 1). For image-based ST data where exact cell locations

Figure 5. Niche-specific gene expression in Slide-seq.

A The spatial mapping with RGB channels displays the simultaneous expression of neighbor-dependent genes in the mouse embryo. Primitive erythroid lineage (PEL) cells dominantly express *Hba-a1* (red) when contacting Definitive erythroid lineage (DEL), and *Cdkn1c* (green) when contacting Limb mesenchyme (LM).
B In the mouse embryo scRNA-seq data, *Hba-a1* and *Cdkn1c* are expressed from PEL.
C In the Spatial mapping for the mouse hippocampus, Entorhinal (Ento) cells express *Nnat* (red) when contacting Choroid (Ch), *Gda* (green) when contacting Neuron.Slc17a6 (N), and *Atp2b1* (blue) when contacting Interneuron (In).
D In the mouse hippocampus scRNA-seq data, *Nnat*, *Gda*, and *Atp2b1* are mostly expressed from Ento.
E In the spatial mapping for the mouse liver cancer, Monocyte cells dominantly express *Marco* (red) when contacting Hepatocyte I, *Vti1a* (green) when contacting Hepatocyte II, and *F13a1* (blue) when contacting Tumor III.
F In the mouse liver cancer snRNA-seq data, *Marco*, *Vti1a*, and *F13a1* are expressed from Monocyte cells.

 © 2023 The Authors

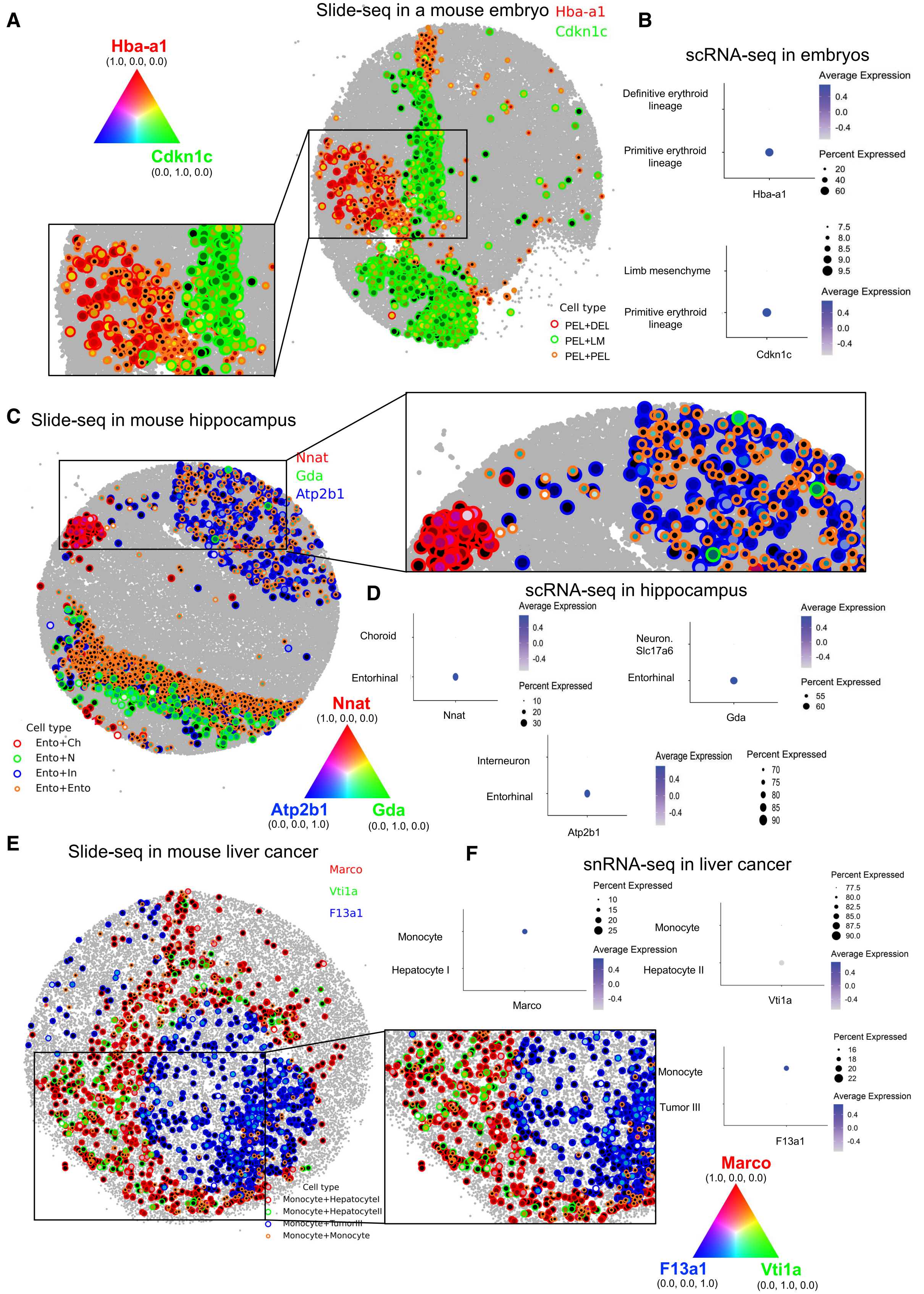
A
Slide-seq in a mouse embryo
Hba-a1
Cdkn1c
Hba-a1
(1.0, 0.0, 0.0)
Cdkn1c
(0.0, 1.0, 0.0)
Cell type
PEL+DEL
PEL+LM
PEL+PEL
B
scRNA-seq in embryos
Definitive erythroid lineage
Primitive erythroid lineage
Hba-a1
Average Expression
0.4
0.0
-0.4
Percent Expressed
20
40
60
Limb mesenchyme
Primitive erythroid lineage
Cdkn1c
7.5
8.0
8.5
9.0
9.5
C
Slide-seq in mouse hippocampus
Nnat
Gda
Atp2b1
Cell type
Ento+Ch
Ento+N
Ento+In
Ento+Ento
Nnat
(1.0, 0.0, 0.0)
Atp2b1
(0.0, 0.0, 1.0)
Gda
(0.0, 1.0, 0.0)
D
scRNA-seq in hippocampus
Choroid
Entorhinal
Nnat
Percent Expressed
10
20
30
Neuron. Slc17a6
Entorhinal
Gda
55
60
Interneuron
Entorhinal
Atp2b1
70
75
80
85
90
E
Slide-seq in mouse liver cancer
Marco
Vti1a
F13a1
Cell type
Monocyte+HepatocyteI
Monocyte+HepatocyteII
Monocyte+TumorIII
Monocyte+Monocyte
F
snRNA-seq in liver cancer
Monocyte
Hepatocyte I
Marco
Percent Expressed
10
15
20
25
Monocyte
Hepatocyte II
Vti1a
77.5
80.0
82.5
85.0
87.5
90.0
Monocyte
Tumor III
F13a1
16
18
20
22
Marco
(1.0, 0.0, 0.0)
F13a1
(0.0, 0.0, 1.0)
Vti1a
(0.0, 1.0, 0.0)

Figure 5.

are available, CellNeighborEX can use various algorithms including Delaunay triangulation, radial distance, and k-nearest neighbors (KNN) to find immediate neighbors. For NGS-based ST data where exact cell locations are not available, we used the heterotypic beads.

To leverage CellNeighborEX effectively, there are three considerations to keep in mind. Firstly, CellNeighborEX works for ST data with single cell or near-cellular resolution. Using heterotypic beads, it successfully identified neighboring cell type-dependent genes in Slide-seq with 10 µm resolution. However, it is not suitable to detect neighbor-dependent genes in ST data with low resolution such as Visium (Ståhl *et al*, 2016) as there could be more than 2 cell types. For the higher resolution ST data such as Seq-Scope (Cho *et al*, 2021), it is possible to use heterotypic beads by rescaling them to 10 µm resolution. Second, careful cell type annotation is needed. The identification of cell contact-dependent genes is heavily affected by cell type annotation as CellNeighborEX categorizes cells according to defined cell types. To minimize yielding false positive, it is recommended to validate annotated cell types by confirming the expression of cell type markers and investigating correlations between true heterotypic spots and artificial heterotypic ones. Third, the number of neighbor-dependent genes can vary across the datasets. Image-based approaches such as seqFISH provide more accurate measurement of RNA quantity against NGS-based ones, which can allow more statistical power in detecting neighbor dependent-genes. However, the number of detectable genes is limited in the image-based approaches, making it hard to detect unknown genes influenced by neighbors.

CellNeighborEX identified neighbor-dependent genes in various ST datasets including mouse embryos (Figs EV1 and EV2), liver cancer (Fig EV3), and mouse hippocampus (Fig 2). Our results indicate that neighbor-dependent genes are found in most of the cell types and tissues, further expanding our observation in the developing mouse embryos (Kim *et al*, 2023).

Interestingly, we found that genes influenced by neighbors were associated with important cell functions. For instance, CellNeighborEX found that neighbor-dependent genes are associated with embryonic development in both seqFISH (Fig EV1D) and Slide-seq V2 (Fig EV2D) data. Our results may suggest that cell contact triggers genes important for further development. CellNeighborEX also provided information about cell types and genes influenced by TME. For instance, CellNeighborEX found that *F13a1* is highly expressed when Monocyte cells contact Tumor cells. *F13a1* has been known to be associated with various cancers including liver cancer (Peltier *et al*, 2018; Porrello *et al*, 2018; Dong *et al*, 2022). In the GO analysis, we found that the neighbor-dependent genes are further associated with cancer metastases (Fig EV3D). Our results show that CellNeighborEX is a useful tool to study the influence of TME in an unbiased way. Besides, the experiment using a co-culture system clearly demonstrated the higher expression of neighbor-dependent genes (Fig 3D). It is also of note that we saw a little overlap with the results obtained from ligand-receptor pairs (Appendix Fig S4). These findings indicate that studying direct cell contact is important to understand cell–cell interactions more thoroughly.

We used heterotypic beads as evidence for cell contact in NGS-based ST data. The neighbor-dependent genes that we identified in Slide-seq data were more highly expressed than artificially generated null models (Appendix Fig S1). From the regression model using the heterotypic beads, we predicted cell types expressing the neighbor-dependent genes (Appendix Fig S3). Our strategy suggests new ways to utilize heterotypic beads in the high-resolution ST data such as Slide-seq.

We observed that cells express specific sets of genes depending on their neighbors (Figs 4 and 5). This niche-specific gene expression partly explains the cause of cellular heterogeneity shown in scRNA-seq data (Fig EV5 and Appendix Fig S7). Also, this suggests that we can annotate sub-clusters of cells based on their neighboring cell types, and further predict neighboring cell types of cells using niche-specific genes.

ST information has been used to understand cell communication. NCEM (Fischer *et al*, 2022) is a graph neural network model to investigate the influence of neighboring cells on gene expression. NCEM uses neighbors in an intermediate range while we focus on immediate neighbors, thereby having lower complexity of neighboring cell types. That allows studying the influence of direct contact between two different cell types. Based on this, the gene expression changes can be explicitly validated in the spatial domain, suggesting niche-specific expression.

To sum up, CellNeighborEX is a new approach to explore transcriptomic changes caused by direct cell contact in ST data. Studying cell contact-dependent gene expression provides opportunities to understand cell–cell interactions between two adjacent cells from a new perspective. It enabled the identification of new genes potentially involved in intercellular communication beyond previous approaches that use ligand-receptor pairs. It also demonstrated gene expression varies depending on neighboring cell types, explaining cellular heterogeneity.

Materials and Methods

Reagents and Tools table

Reagent/Resource	Reference or source	Identifier or catalog number
**Experimental models**		
C57BL/6J (M. musculus)	Envigo	C57BL/6NHsd
**Recombinant DNA**		
pD2109-CMV-GFP	ATUM	361593
**Antibodies**		
CD34:FITC	BD Biosciences	#562608

 © 2023 The Authors

Reagents and Tools table (continued)

Reagent/Resource	Reference or source	Identifier or catalog number
VEGFR2:PE	BD Biosciences	#121905
VEGFR1:Alexa594	Novus Biological	#NB100-527AF594
CD11b: BUV395	BD Biosciences	#565976
ACSA2:APC	Miltenyi Biotec	#130-117-535
**Oligonucleotides and sequence-based reagents**		
Gapdh (F)	This study	5′-CATCACTGCCACCCAGAAGACTG-3′
Gapdh (R)	This study	5′-ATGCCAGTGAGCTTCCCGTTCAG-3′
Fabp7 (F)	This study	5′-TGGGAAACGTGACCAAACCA-3′
Fabp7 (R)	This study	5′-AGCTTGTCTCCATCCAACCG-3′
Trf (F)	This study	5′-AGACTTCGAGTTGCTCTGCC-3′
Trf (R)	This study	5′-CAGAAATTGCCGGTGCAGTC-3′
Dbi (F)	This study	5′-TGCGCTCTGTGACTTGATTG-3′
Dbi (R)	This study	5′-ATCGCCCACAGTAGCTTGTTT-3′
**Software**		
Spectraflo 1.0.5	https://cytekbio.com/pages/spectro-flo	
CellNeighborEX	https://github.com/hkim240/CellNeighborEX	
Seurat 3.2.2	https://satijalab.org/seurat/	
Scanpy 1.9.1	https://github.com/scverse/scanpy	
Squidpy 1.2.3	https://github.com/scverse/squidpy	
RCTD (Spacexr)	https://github.com/dmcable/spacexr	
NicheNet 1.0.0	https://github.com/saeyslab/nichenetr	
DoubletFinder 2.0	https://github.com/chris-mcginnis-ucsf/DoubletFinder	
clusterProfiler 4.6.0	https://github.com/YuLab-SMU/clusterProfiler	
**Other**		
Cytek Aurora Cell Sorter	Cytekbio	
QuantStudioTM 5	Applied Biosystems	
PureLink RNA Mini kit	Thermo Fisher Scientific	#12183020
PowerUp SYBR Green master mix	Thermo Fisher Scientific	#A25741
Superscript Vilo cDNA synthesis kit	Thermo Fisher Scientific	#11755050

Methods and Protocols

Data preprocessing

For seqFISH data from a mouse embryo (Lohoff *et al*, 2022), we used the gene expression data and annotated cell types pre-processed by Squidpy (Palla *et al*, 2022a; Data ref: Palla *et al*, 2022b). For Slide-seq data from a mouse embryo (Stickels *et al*, 2021a; Data ref: Stickels *et al*, 2021b), hippocampus (Stickels *et al*, 2021a; Data ref: Stickels *et al*, 2021b), and liver cancer (Zhao *et al*, 2022a; Data ref: Zhao *et al*, 2022b), we obtained them from Puck_190926_03, Puck_200115_08, and mouse_liver_met_2_rna_201002_04, respectively. For the embryo and liver cancer, samples that have unique feature counts < 200 were filtered out. For the hippocampus, all samples were used without the filtering because the samples with unique feature counts < 200 take up a considerable percentage of the total samples (*i.e.*, about 40%). The values of the count matrix per dataset were log-normalized and then top 2000 variable genes as well as cell type markers (Dataset EV4) were selected.

For liver cancer snRNA-seq data, we pre-processed paired dataset (Zhao *et al*, 2022a; Data ref: Zhao *et al*, 2022b) given with the Slide-seq data from the mouse liver cancer. The genes expressed in < 3 nuclei were filtered out. The samples that have unique feature counts < 200 and mitochondrial RNA larger than 1 % were filtered out. Additionally, approximately 10 percent of doublets detected by DoubletFinder (McGinnis *et al*, 2019) were removed. After clustering analysis, we annotated cell clusters using the information of cell type markers accompanied with the snRNA-seq dataset. The analyses mentioned above were all performed with Seurat 3.2.2 (Satija *et al*, 2015).

Cell type inference of slide-seq spots

We used RCTD (Cable *et al*, 2021) to identify the cell types of spots in Slide-seq. To run RCTD, we trained RCTD with scRNA-seq or snRNA-seq datasets with annotated cell types. For the embryo, we used a scRNA-seq dataset (Cao *et al*, 2019a; Data ref: Cao *et al*, 2019b) at E12.5 equivalent to the developmental stage of Slide-seq embryo. It consists of 26,183 genes and 270,197 cells assigned into 37 cell types

(Appendix Fig S8A). For the hippocampus, we obtained a scRNA-seq dataset (Saunders *et al*, 2018a; Data ref: Saunders *et al*, 2018b) from DropViz. It is composed of 27,953 genes and 113,507 cells assigned into 17 cell types (Appendix Fig S8B). For the liver cancer data, we used the paired snRNA-seq dataset (Zhao *et al*, 2022a; Data ref: Zhao *et al*, 2022b) given with the Slide-seq data from mouse liver cancer. The pre-processed snRNA-seq dataset consists of 24,098 genes and 11,683 nuclei assigned into 14 cell types (Appendix Fig S8C). Training RCTD, we predicted the cell types of spots in Slide-seq. The simulation was performed under doublet mode (Cable *et al*, 2021) that constraints each spot to contain up to two cell types, which is recommended for data with fine resolution such as Slide-seq. Using RCTD, we identified the cell types of spots and further estimated the cell type proportions for each spot.

The results on the inferred cell types were additionally validated through correlation analysis. We examined correlation between the true heterotypic spots annotated by RCTD and artificial heterotypic spots. Specifically, we generated artificial heterotypic spots by combining two homotypic spots based on the cell type proportions of heterotypic spots obtained by RCTD (Appendix Fig S1).

With repeated random sampling, we created 100 artificial heterotypic spots for each heterotypic spot. Next, we calculated Pearson's correlation coefficients based on the gene expression values between the true heterotypic spots and many combinatorial types of artificial heterotypic spots (Appendix Fig S2). If the cell types of true heterotypic spots are consistent with those of artificial ones with the largest Pearson's coefficient, we regarded the cell types of the true heterotypic spots as validated. On top of the correlation analysis, the inferred cell types were validated by cell type markers (Dataset EV4) accompanied with the scRNA-seq (Cao *et al*, 2019a; Data ref: Cao *et al*, 2019b; Saunders *et al*, 2018a; Data ref: Saunders *et al*, 2018b) or snRNA-seq (Zhao *et al*, 2022a; Data ref: Zhao *et al*, 2022b) datasets.

CellNeighborEX—Neighbor-dependent gene expression analysis

We studied neighbor-dependent gene expression by comparing heterotypic groups (heterotypic neighbors for seqFISH, heterotypic spots for Slide-seq) with homotypic groups (homotypic neighbors for seqFISH, homotypic spots for Slide-seq). We identified genes up- and down-regulated by direct cell contact. We carried out rigorous statistical analysis between the two groups. We determined whether to use parametric or non-parametric two-sided tests depending on the sample size of groups. Additionally, the normality test using the Shapiro–Wilk test is also available in CellNeighborEX. When both samples were larger than sample size 30, we chose parametric tests under the normality assumption. To be specific, we conducted the Student's *t*-test for equal variances and the Welch's *t*-test for unequal variances, where the two-sample *F*-test was used to test whether the variances are equal or not.

For the Student's *t*-test, the *t* statistic is calculated as follows:

$$t = \frac{\overline{X_1} - \overline{X_2}}{s_p\sqrt{\frac{1}{n_1} + \frac{1}{n_2}}}$$

where $\overline{X_i}$ is the mean of expression values in group i ($i = 1,2$). In the equations, group 1 and group 2 represent heterotypic group and homotypic group, respectively. n_i is the sample size of group i. s_p is the pooled standard deviation of the two groups:

$$s_p = \sqrt{\frac{(n_1-1)s_1^2 + (n_2-1)s_2^2}{n_1 + n_2 - 2}}$$

where s_i is the standard deviation of group i.

For the Welch's *t*-test, the *t* statistic is computed as follows:

$$t = \frac{\overline{X_1} - \overline{X_2}}{s_{\overline{\Delta}}}$$

where

$$s_{\overline{\Delta}} = \sqrt{\frac{s_1^2}{n_1} + \frac{s_2^2}{n_2}}$$

Meanwhile, when the sample size of at least one sample was smaller than 30, we performed the Mann–Whitney *U* test as a non-parametric test. The *U* statistic is calculated as follows:

$$U = \min(U_1, U_2)$$

$$U_1 = n_1 n_2 + \frac{n_1(n_1+1)}{2} - R_1$$

$$U_2 = n_1 n_2 + \frac{n_2(n_2+1)}{2} - R_2$$

where R_1 is the sum of the ranks for the heterotypic group, and R_2 is the sum of the ranks for the homotypic group.

In DE analysis, the log ratio > 0.4 and *P*-value < 0.01 were used as criteria for differential expression. For the seqFISH data, FDR < 0.05 was added as an additional criterion.

CellNeighborEX—Verification of cell–cell interactions in the heterotypic spots of slide-seq data

We developed a null model to verify that individual heterotypic spots represent two different cell types interacting with each other. Our null model refers to artificial heterotypic spots (Appendix Fig S1). In contrast with the true heterotypic spots, the artificial heterotypic spots indicate two different cell types just combined without cell–cell interactions. We compared the true heterotypic spots with the artificial heterotypic spots to confirm the statistical significance of the neighbor-dependent genes. The significant neighbor-dependent genes mean that their expression resulted from interacting two cell types. The same statistical tests as the neighbor-dependent gene expression analysis were applied. The log ratio > 0.4, *P*-value < 0.01, and FDR < 0.01 were used as criteria for differential expression.

Finding cell types expressing neighbor-dependent genes in the heterotypic spots of slide-seq

Heterotypic spots of Slide-seq represent interacting two different cell types. For the genes up-regulated by cell contact, it is challenging to find from which cell type the expression of the neighbor-dependent genes comes between the two cell types. We created linear regression models to find the origin of the expression. For instance, we suppose that g is a neighbor-dependent gene found from the heterotypic spots of $A + B$. For n data pairs $\{(x_i, y_i), i = 1, 2, \ldots, n\}$, the regression model is as follows:

© 2023 The Authors

$$y_i = \alpha + \beta x_i + \varepsilon_i$$

where n is the number of $A+B$ heterotypic spots expressing g. x_i is the proportion of A, $1-x_i$ is the proportion of B, and y_i is the expression value of g in heterotypic spot $A+B_i$. We use the ordinary least squares method to find the intercept $(\widehat{\alpha})$ and slope $(\widehat{\beta})$ illustrating the best fit line to the n data pairs. Estimated $\widehat{\alpha}$ and $\widehat{\beta}$ are computed as follows:

$$\widehat{\alpha} = \overline{y} - \left(\widehat{\beta \overline{x}}\right),$$

$$\widehat{\beta} = \frac{\sum_{i=1}^{n}(x_i - \overline{x})(y_i - \overline{y})}{\sum_{i=1}^{n}(x_i - \overline{x})^2}$$

where $\overline{x}$ and $\overline{y}$ are the mean of the x_i and y_i, respectively. If slope $\widehat{\beta}$ is positive, it indicates that the expression value of g increases as the proportion of cell type A grows. From this, we can infer that the expression of g comes from cell type A. If $\widehat{\beta}$ is negative, the expression of g decreases as the proportion of cell type A becomes larger. It means that the expression of g increases as the proportion of cell type B grows. That is, the expression of g comes from cell type B.

We applied the regression models to the neighbor-dependent genes obtained in Slide-seq data when the number of the heterotypic spots is large enough. We additionally used scRNA-seq or snRNA-seq data (Appendix Fig S9) and found that the cell types inferred from the single cell or single nucleus data are considerably consistent with the cell types predicted from the statistically significant regression models (*i.e.*, P-value < 0.05; Appendix Fig S3 and Dataset EV5).

Spatial visualization with RGB color channels

We used RGB coordinates (r, g, b) composed of values between 0 and 1 to observe how gene expression varies depending on neighboring cell types. We first selected one cell type and then collected multiple heterotypic groups where the selected cell type is included. For example, if the cell type of interest is A, heterotypic groups $A+B$, $A+C$, and $A+D$ are collected. If *gene* γ, *gene* δ, and gene ρ are neighbor-dependent genes found from the heterotypic spots of $A+B$, $A+C$, and $A+D$ respectively, red, green, and blue channels are assigned to the three neighbor-dependent genes: *gene* γ is red (R), *gene* δ is green (G), and gene ρ is blue (B). To normalize the expression values between 0 and 1, we divided them by the maximum expression value in each heterotypic group. In case that there are two genes, the value of the blue channel is fixed as zero. The expression of the three neighbor-dependent genes is simultaneously visualized by the RGB channels.

Identification of ligands, receptors, and downstream targets

We used NicheNet (Browaeys *et al*, 2020) to compare our neighbor-dependent genes with already known ligands, receptors, and their target genes. We ran NicheNet on the seqFISH and Slide-seq data to see if NicheNet can detect our neighbor-dependent genes. For the seqFISH, we investigated cells corresponding to the heterotypic neighbors (22 types of heterotypic neighbors in the embryo) where our neighbor-dependent genes were identified. We set the centered cell type as a sender and the neighboring cell type as a receiver, vice versa. In the case of Slide-seq, we used homotypic spots corresponding to the heterotypic pairs (9 heterotypic pairs in the embryo, 10 heterotypic pairs in the liver cancer, and 21 heterotypic pairs in the hippocampus). We set the respective homotypic spots as a sender and a receiver by turns.

To validate if the heterotypic spots in Slide-seq are useful to study cell–cell interactions, we examined the heterotypic spots. We set the heterotypic spots as a receiver as well as a sender (autocrine mode in NicheNet). Highly expressed ligand-receptor-target genes detected by NicheNet mean being expressed in at least 10% of cells in one cluster.

Flow cytometry and cell sorting of astrocyte and endothelial tip populations

We used C57BL/6 male mice obtained from Envigo. All animal studies were conducted using a protocol approved by the Virginia Commonwealth University Institutional Animal Care and Use Committee. Cells from the male C57BL/6 mouse hippocampus were isolated and incubated with cell surface antibodies specific to Endothelial Tip (EnT) and Astrocyte cell populations. Following the incubation, these populations were sorted using Cytek Aurora Cell Sorter, and data were analyzed by Spectraflo version 1.0.5 software as follows. FSC/SSC gates were used to define a homogeneous population, and FSC-H/FSC-A gates were used to sort singlets exclusively. For the purpose of isolating the EnT cells, the hippocampus cells were suspended in FACS buffer 2 (PBS, 1 mM EDTA, 25 mM HEPES pH 7, 2% FBS) and stained with CD34: FITC (BD Biosciences #562608), VEGFR2: PE (BD Biosciences #121905), and VEGFR1:Alexa594 (Novus Biological #NB100-527AF594). The VEGFR1-negative cells were selected using the SSC/VEGFR1 gate as described previously (Suchting *et al*, 2007), from which CD34/VEGFR2 (Siemerink *et al*, 2013) gate was used to select double-positive populations. For the purpose of isolating the Astrocyte cells, the hippocampus cells were suspended in FACS buffer 2 and stained with ACSA2: APC (Miltenyi Biotec #130-117-535) and CD11b: BUV395 (BD Biosciences #565976). Here, the SSC/CD11b gate was used to select CD11b negative (Pan & Wan, 2020) cells. Then we used the SSC/ACSA2 to specifically isolate Astrocyte cells by selecting the ACSA2 positive population (Pan & Wan, 2020). Isolated EnT and Astrocyte cells were maintained in culture using the Human Endothelial Serum Free Medium and Astrocytes Medium (Thermo Fisher) at 37°C, 5% CO2 incubator. A subpopulation of the Astrocyte cells was infected with 109 TU/ml of GFP comet-pD2109-CMV lentiviral particles (ATUM) and 5 μg/ml of polybrene. After 24 h infection, GFP-positive A cells were selected by GFP expression using Cytek Aurora Cell Sorter. Following a 48 h co-culture of GFP+ Astrocyte and GFP- EnT cells in a 1:1 ratio, the Cytek Aurora Cell Sorter was used to sort the GFP-positive Astrocyte cells and GFP-negative EnT cells according to their GFP expression using SSC/GFP gate. Alternatively, we performed a media exchange experiment in which EnT cells were cultured in Astrocyte cells-derived media while Astrocyte cells were cultured in EnT cells-derived media.

Endothelia tip and astrocyte contact-specific gene quantification by RT–qPCR

We isolated total RNA from EnT and Astrocyte cells individual cultures, cocultures, and cell exchange cultures using the PureLink RNA Mini kit, as per the manufacturer's instruction, and eluted total RNA

© 2023 The Authors

in 50 µl RNase/DNase-free H2O. Then, we reverse-transcribed to cDNA 10 ng of total RNA using Superscript Vilo cDNA synthesis kit. Finally, we performed real-time PCR (qPCR) in QuantStudioTM 5 (Applied Biosystems) using PowerUp SYBR Green master mix (Thermo Fisher Scientific) and the following reaction conditions. The initial denaturation step was performed at 95°C for 2 min, followed by 40 cycles of 95°C for 15 s and 60°C for 60 s. We used the comparative C_T method ($\Delta\Delta C_t$) to quantify relative gene expression, normalizing the expression of our target genes with the housekeeping gene *Gapdh*. All samples were run using the following primers: *Gapdh*: 5′-CATCACTGCCACCCAGAAGACTG-3′(F) and 5′-ATGCCAGTGAGCTT CCCGTTCAG-3′ (R); *Fabp7*: 5′-TGGGAAACGTGACCAAACCA-3′ (F) and 5′-AGCTTGTCTCCATCCAACCG-3′ (R); *Trf*: 5′-AGACTTCGAGTT GCTCTGCC-3′ (F) and 5′-CAGAAATTGCCGGTGCAGTC-3′ (R); and *Dbi*: 5′-TGCGCTCTGTGACTTGATTG-3′ (F) and 5′-ATCGCCCACAGT AGCTTGTTT-3′ (R). All genes were analyzed using 5 biological replicates. We conducted Shapiro–Wilk test to determine if the gene expression values followed a normal distribution and then performed the Student's *t*-test for equal variances and the Welch's *t*-test for unequal variances, where the two-sample *F*-test was used to test whether the variances are equal or not. The statistical analysis for gene expression was performed with a confidence interval of 95%.

Analyzed publicly available datasets

- Pre-processed subset seqFISH data in a mouse embryo: Squidpy (https://squidpy.readthedocs.io/en/stable/api/squidpy.datasets.seqfish.html).
- Slide-seq V2 data in a mouse embryo and hippocampus: Single Cell Portal (https://singlecell.broadinstitute.org/single_cell/study/SCP815/sensitive-spatial-genome-wide-expression-profiling-at-cellular-resolution#study-summary).
- Slide-seq and snRNA-seq data in mouse liver cancer: Single Cell Portal (https://singlecell.broadinstitute.org/single_cell/study/SCP1278/spatial-genomics-enables-multi-modal-study-of-clonal-heterogeneity-in-tissues).
- scRNA-seq data in a mouse embryo: Gene Expression Omnibus GSE119945 (https://www.ncbi.nlm.nih.gov/geo/query/acc.cgi?acc=GSE119945).
- scRNA-seq data in mouse hippocampus: DropViz (http://dropviz.org/).

Data availability

The datasets and codes for neighbor-dependent gene expression analysis are available at Figshare https://figshare.com/articles/dataset/Datasets_for_neighbor-dependent_gene_expression_analysis_with_CellNeighborEX/24130395 and Github https://github.com/hkim240/Cell NeighborEX.

Expanded View for this article is available online.

Acknowledgements
We appreciate Dr. Patrick Martin for critical reading of the manuscript. We appreciate the institutional support from Cedars-Sinai Medical Center and Lundbeck Foundation (R313-2019-421) to KJW. Authors are supported by NIH-NIC (R01CA242003, U54CA283762, U54CA233444, and U54CA23444-03S1) and the Joseph and Ann Matella Fund for Pancreatic Cancer Research to JT; VCU Massey Cancer Center and School of Medicine start-up funds, Swiss Cancer League, Seeds of Science, UAMS, SNSF, Fundação para a Ciência e a Tecnologia, Fundamental Mandates (Stichting tegen Kanker—Fondation contre le Cancer) to RG; VCU Massey Cancer Center and School of Medicine start-up funds, La Caixa Funding LCF/BQ/PR20/11770006 to EM.

Author contributions
Hyobin Kim: Conceptualization; software; formal analysis; investigation; visualization; methodology; writing – original draft; writing – review and editing. **Amit Kumar:** Formal analysis; investigation; visualization; writing – review and editing. **Cecilia Lövkvist:** Formal analysis; investigation; visualization; writing – review and editing. **António M Palma:** Formal analysis; investigation; visualization; writing – review and editing. **Patrick Martin:** Formal analysis; investigation; writing – review and editing. **Junil Kim:** Formal analysis; investigation; writing – review and editing. **Praveen Bhoopathi:** Formal analysis; investigation. **Jose Trevino:** Formal analysis; investigation. **Paul Fisher:** Formal analysis; investigation.
Esha Madan: Formal analysis; investigation. **Rajan Gogna:** Formal analysis; supervision; funding acquisition; investigation; writing – review and editing. **Kyoung Jae Won:** Conceptualization; supervision; funding acquisition; methodology; writing – original draft; project administration; writing – review and editing.

Disclosure and competing interests statement
The authors declare that they have no conflict of interest.

References

Arnol D, Schapiro D, Bodenmiller B, Saez-Rodriguez J, Stegle O (2019) Modeling cell-cell interactions from spatial molecular data with spatial variance component analysis. *Cell Rep* 29: 202–211.e6

Barone V, Lang M, Krens SG, Pradhan SJ, Shamipour S, Sako K, Sikora M, Guet CC, Heisenberg C-P (2017) An effective feedback loop between cell-cell contact duration and morphogen signaling determines cell fate. *Dev Cell* 43: 198–211.e12

Boisset J-C, Vivié J, Grün D, Muraro MJ, Lyubimova A, Van Oudenaarden A (2018) Mapping the physical network of cellular interactions. *Nat Methods* 15: 547–553

Browaeys R, Saelens W, Saeys Y (2020) NicheNet: modeling intercellular communication by linking ligands to target genes. *Nat Methods* 17: 159–162

Cable DM, Murray E, Zou LS, Goeva A, Macosko EZ, Chen F, Irizarry RA (2021) Robust decomposition of cell type mixtures in spatial transcriptomics. *Nat Biotechnol* 40: 517–526

Cable DM, Murray E, Shanmugam V, Zhang S, Zou LS, Diao M, Chen H, Macosko EZ, Irizarry RA, Chen F (2022) Cell type-specific inference of differential expression in spatial transcriptomics. *Nat Methods* 19: 1076–1087

Cao J, Spielmann M, Qiu X, Huang X, Ibrahim DM, Hill AJ, Zhang F, Mundlos S, Christiansen L, Steemers FJ (2019a) The single-cell transcriptional landscape of mammalian organogenesis. *Nature* 566: 496–502

Cao J, Spielmann M, Qiu X, Huang X, Ibrahim DM, Hill AJ, Zhang F, Mundlos S, Christiansen L, Steemers FJ (2019b) Gene Expression Omnibus GSE119945 (https://www.ncbi.nlm.nih.gov/geo/query/acc.cgi?acc=GSE119945). [DATASET]

Chen KH, Boettiger AN, Moffitt JR, Wang S, Zhuang X (2015) Spatially resolved, highly multiplexed RNA profiling in single cells. *Science* 348: aaa6090

 © 2023 The Authors

Cho C-S, Xi J, Si Y, Park S-R, Hsu J-E, Kim M, Jun G, Kang HM, Lee JH (2021) Microscopic examination of spatial transcriptome using Seq-Scope. *Cell* 184: 3559–3572.e22

Chung M-I, Nascone-Yoder NM, Grover SA, Drysdale TA, Wallingford JB (2010) Direct activation of Shroom3 transcription by Pitx proteins drives epithelial morphogenesis in the developing gut. *Development* 137: 1339–1349

Cover T, Hart P (1967) Nearest neighbor pattern classification. *IEEE Trans Inf Theory* 13: 21–27

Delaunay B (1934) Sur la Sphere Vide, bull. Acad. Science USSR VII: Class. *Sci Mat Nat* 1934: 793–800

Dong W, Xia Z, Chai Z, Qiu Z, Wang X, Yang Z, Wang J, Zhang T, Zhang Q, Jin J (2022) Proteomic analysis of small extracellular vesicles from the plasma of patients with hepatocellular carcinoma. *World J Surg Oncol* 20: 387

Efremova M, Vento-Tormo M, Teichmann SA, Vento-Tormo R (2020) CellPhoneDB: inferring cell–cell communication from combined expression of multi-subunit ligand–receptor complexes. *Nat Protoc* 15: 1484–1506

Eng C-HL, Lawson M, Zhu Q, Dries R, Koulena N, Takei Y, Yun J, Cronin C, Karp C, Yuan G-C *et al* (2019) Transcriptome-scale super-resolved imaging in tissues by RNA seqFISH+. *Nature* 568: 235–239

Fischer DS, Schaar AC, Theis FJ (2022) Modeling intercellular communication in tissues using spatial graphs of cells. *Nat Biotechnol* 41: 332–336

Fix E, Hodges J (1951) Discriminatory analysis: nonparametric discrimination: consistency properties. Report. 4. *T USAF School of Aviation Medicine*

Garcia-Alonso L, Handfield L-F, Roberts K, Nikolakopoulou K, Fernando RC, Gardner L, Woodhams B, Arutyunyan A, Polanski K, Hoo R *et al* (2021) Mapping the temporal and spatial dynamics of the human endometrium *in vivo* and *in vitro*. *Nat Genet* 53: 1698–1711

Giladi A, Cohen M, Medaglia C, Baran Y, Li B, Zada M, Bost P, Blecher-Gonen R, Salame T-M, Mayer JU *et al* (2020) Dissecting cellular crosstalk by sequencing physically interacting cells. *Nat Biotechnol* 38: 629–637

Hannezo E, Heisenberg C-P (2019) Mechanochemical feedback loops in development and disease. *Cell* 178: 12–25

Ji J, Zhao L, Budhu A, Forgues M, Jia H-L, Qin L-X, Ye Q-H, Yu J, Shi X, Tang Z-Y *et al* (2010) Let-7g targets collagen type I α2 and inhibits cell migration in hepatocellular carcinoma. *J Hepatol* 52: 690–697

Kim J, Rothová MM, Madan E, Rhee S, Weng G, Palma AM, Liao L, David E, Amit I, Hajkarim MC *et al* (2023) Neighbor-specific gene expression revealed from physically interacting cells during mouse embryonic development. *Proc Natl Acad Sci USA* 120: e2205371120

Lanctôt C, Lamolet B, Drouin J (1997) The bicoid-related homeoprotein Ptx1 defines the most anterior domain of the embryo and differentiates posterior from anterior lateral mesoderm. *Development* 124: 2807–2817

Li R, Yang X (2022) *De novo* reconstruction of cell interaction landscapes from single-cell spatial transcriptome data with DeepLinc. *Genome Biol* 23: 1–24

Li D, Ding J, Bar-Joseph Z (2021) Identifying signaling genes in spatial single-cell expression data. *Bioinformatics* 37: 968–975

Lohoff T, Ghazanfar S, Missarova A, Koulena N, Pierson N, Griffiths J, Bardot E, Eng C-H, Tyser R, Argelaguet R *et al* (2022) Integration of spatial and single-cell transcriptomic data elucidates mouse organogenesis. *Nat Biotechnol* 40: 74–85

Lubeck E, Coskun AF, Zhiyentayev T, Ahmad M, Cai L (2014) Single-cell in situ RNA profiling by sequential hybridization. *Nat Methods* 11: 360–361

McGinnis CS, Murrow LM, Gartner ZJ (2019) DoubletFinder: doublet detection in single-cell RNA sequencing data using artificial nearest neighbors. *Cell Syst* 8: 329–337.e4

Nishida-Aoki N, Gujral TS (2019) Emerging approaches to study cell–cell interactions in tumor microenvironment. *Oncotarget* 10: 785–797

Palla G, Spitzer H, Klein M, Fischer D, Schaar AC, Kuemmerle LB, Rybakov S, Ibarra IL, Holmberg O, Virshup I *et al* (2022a) Squidpy: a scalable framework for spatial omics analysis. *Nat Methods* 19: 171–178

Palla G, Spitzer H, Klein M, Fischer D, Schaar AC, Kuemmerle LB, Rybakov S, Ibarra IL, Holmberg O, Virshup I *et al* (2022b) Squidpy. https://squidpy.readthedocs.io/en/stable/api/squidpy.datasets.seqfish.html [DATASET]

Pan J, Wan J (2020) Methodological comparison of FACS and MACS isolation of enriched microglia and astrocytes from mouse brain. *J Immunol Methods* 486: 112834

Peltier J, Roperch J-P, Audebert S, Borg J-P, Camoin L (2018) Activation peptide of the coagulation factor XIII (AP-F13A1) as a new biomarker for the screening of colorectal cancer. *Clin Proteomics* 15: 1–11

Pham D, Tan X, Xu J, Grice LF, Lam PY, Raghubar A, Vukovic J, Ruitenberg MJ, Nguyen Q (2020) stLearn: integrating spatial location, tissue morphology and gene expression to find cell types, cell-cell interactions and spatial trajectories within undissociated tissues. *bioRxiv* https://doi.org/10.1101/2020.05.31.125658 [PREPRINT]

Porrello A, Leslie PL, Harrison EB, Gorentla BK, Kattula S, Ghosh SK, Azam SH, Holtzhausen A, Chao YL, Hayward MC *et al* (2018) Factor XIIIA—expressing inflammatory monocytes promote lung squamous cancer through fibrin cross-linking. *Nat Commun* 9: 1988

Radhakrishnan K, Halasz A, Vlachos D, Edwards JS (2010) Quantitative understanding of cell signaling: the importance of membrane organization. *Curr Opin Biotechnol* 21: 677–682

Rodriques SG, Stickels RR, Goeva A, Martin CA, Murray E, Vanderburg CR, Welch J, Chen LM, Chen F, Macosko EZ (2019) Slide-seq: a scalable technology for measuring genome-wide expression at high spatial resolution. *Science* 363: 1463–1467

Sammar M, Aigner S, Hubbe M, Schirrmacher V, Schachner M, Vestweber D, Altevogt P (1994) Heat-stable antigen (CD24) as ligand for mouse P-selectin. *Int Immunol* 6: 1027–1036

Satija R, Farrell JA, Gennert D, Schier AF, Regev A (2015) Spatial reconstruction of single-cell gene expression data. *Nat Biotechnol* 33: 495–502

Saunders A, Macosko EZ, Wysoker A, Goldman M, Krienen FM, de Rivera H, Bien E, Baum M, Bortolin L, Wang S *et al* (2018a) Molecular diversity and specializations among the cells of the adult mouse brain. *Cell* 174: 1015–1030.e16

Saunders A, Macosko EZ, Wysoker A, Goldman M, Krienen FM, de Rivera H, Bien E, Baum M, Bortolin L, Wang S *et al* (2018b) DropViz. http://dropviz.org/ [DATASET]

Shao X, Li C, Yang H, Lu X, Liao J, Qian J, Wang K, Cheng J, Yang P, Chen H *et al* (2022) Knowledge-graph-based cell-cell communication inference for spatially resolved transcriptomic data with SpaTalk. *Nat Commun* 13: 4429

Siemerink MJ, Klaassen I, Van Noorden CJ, Schlingemann RO (2013) Endothelial tip cells in ocular angiogenesis: potential target for anti-angiogenesis therapy. *J Histochem Cytochem* 61: 101–115

Solini GE, Dong C, Saha M (2017) Embryonic transplantation experiments: past, present, and future. *Trends Dev Biol* 10: 13–30

Spemann H, Mangold H (2003) Induction of embryonic primordia by implantation of organizers from a different species. 1923. *Int J Dev Biol* 45: 13–38

Ståhl PL, Salmén F, Vickovic S, Lundmark A, Navarro JF, Magnusson J, Giacomello S, Asp M, Westholm JO, Huss M *et al* (2016) Visualization and

© 2023 The Authors

analysis of gene expression in tissue sections by spatial transcriptomics. *Science* 353: 78–82

Stickels RR, Murray E, Kumar P, Li J, Marshall JL, Di Bella DJ, Arlotta P, Macosko EZ, Chen F (2021a) Highly sensitive spatial transcriptomics at near-cellular resolution with Slide-seqV2. *Nat Biotechnol* 39: 313–319

Stickels RR, Murray E, Kumar P, Li J, Marshall JL, Di Bella DJ, Arlotta P, Macosko EZ, Chen F (2021b) Single Cell Portal. https://singlecell.broadinstitute.org/single_cell/study/SCP815/sensitive-spatial-genome-wide-expression-profiling-at-cellular-resolution#study-summary [DATASET]

Suchting S, Freitas C, le Noble F, Benedito R, Bréant C, Duarte A, Eichmann A (2007) The Notch ligand Delta-like 4 negatively regulates endothelial tip cell formation and vessel branching. *Proc Natl Acad Sci USA* 104: 3225–3230

Tanevski J, Flores ROR, Gabor A, Schapiro D, Saez-Rodriguez J (2022) Explainable multiview framework for dissecting spatial relationships from highly multiplexed data. *Genome Biol* 23: 1–31

Tran TQ, Kioussi C (2021) Pitx genes in development and disease. *Cell Mol Life Sci* 78: 4921–4938

Xu S, Xu H, Wang W, Li S, Li H, Li T, Zhang W, Yu X, Liu L (2019) The role of collagen in cancer: from bench to bedside. *J Transl Med* 17: 1–22

Yang BA, Westerhof TM, Sabin K, Merajver SD, Aguilar CA (2021) Engineered tools to study intercellular communication. *Adv Sci* 8: 2002825

Zhao T, Chiang ZD, Morriss JW, LaFave LM, Murray EM, Del Priore I, Meli K, Lareau CA, Nadaf NM, Li J *et al* (2022a) Spatial genomics enables multi-modal study of clonal heterogeneity in tissues. *Nature* 601: 85–91

Zhao T, Chiang ZD, Morriss JW, LaFave LM, Murray EM, Del Priore I, Meli K, Lareau CA, Nadaf NM, Li J *et al* (2022b) Single Cell Portal. https://singlecell.broadinstitute.org/single_cell/study/SCP1278/spatial-genomics-enables-multi-modal-study-of-clonal-heterogeneity-in-tissues [DATASET]

License: This is an open access article under the terms of the Creative Commons Attribution License, which permits use, distribution and reproduction in any medium, provided the original work is properly cited.

© 2023 The Authors

Expanded View Figures

Figure EV1. Neighbor dependent-genes identified by CellNeighborEX in mouse embryo seqFISH data.

A Gut tube cells adjacent to Neural crest cells (Gut tube/Neural crest) ($n = 694$) were compared with Gut tube cells proximal to other Gut tube cells (Gut tube/Gut tube; $n = 33$; log ratio > 0.4, FDR < 0.05). The statistical test was chosen among the two tailed Student's t-test, Welch's t-test, or Wilcoxon rank sum test depending on the sample size and heterogeneity of variance test. The volcano plot shows 23 up-regulated including *Pitx1* and 21 down-regulated genes including *Foxa1* in Gut tube/Neural crest.

B The spatial visualization displays that Gut tube cells adjacent to Neural crest more highly express *Pitx1*.

C For genes up-regulated by cell contact in the mouse embryo seqFISH data, GO analysis shows that the GO terms are associated with embryonic development. The bar plot presents top 20 GO terms.

© 2023 The Authors

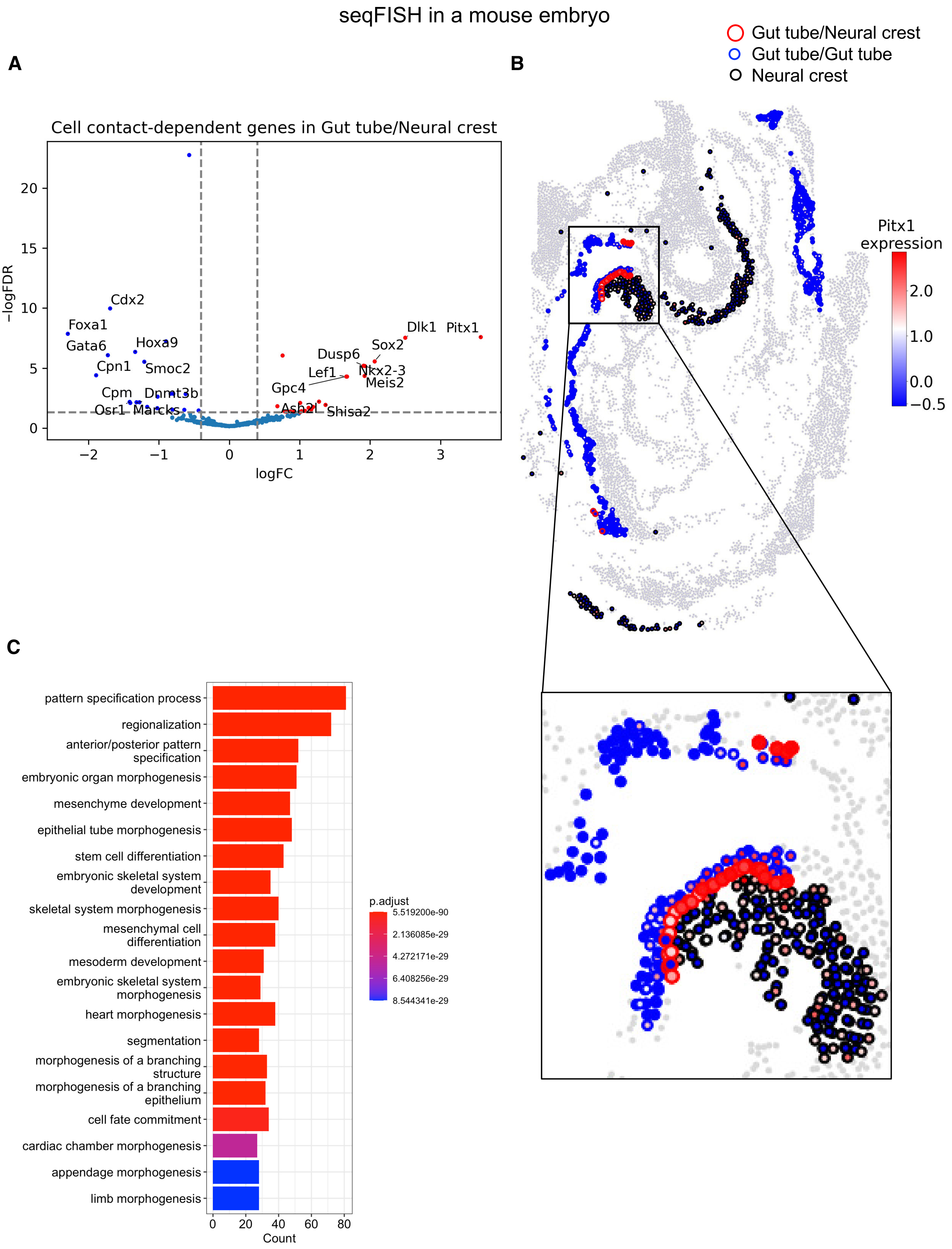

Figure EV1.

 © 2023 The Authors

Figure EV2. Transcriptomic change due to direct cell contact in mouse embryo Slide-seq data.

A The heterotypic spots of Endothelial and Lens cells (En + L) ($n = 120$) were compared with the respective homotypic spots (En [$n = 85$], L [=52]; log ratio > 0.4, *P*-value < 0.01) and additionally with the artificial heterotypic spots (FDR < 0.01). The statistical test was chosen among the two tailed Student's *t*-test, Welch's *t*-test, or Wilcoxon rank sum test depending on the sample size and heterogeneity of variance test. The volcano plot displays 17 up-regulated genes including *Cd24a*. In the heatmap, the genes are more highly expressed in En + L. The heterotypic spots also express both En and L markers.

B The spatial visualization shows the higher expression level of *Cd24a* in En + L.

C In the mouse embryo scRNA-seq data, it was confirmed that *Cd24a* is expressed in En.

D In GO analysis, the GO terms for the genes up-regulated by neighbors are associated with embryonic development. The bar plot shows top 20 GO terms.

© 2023 The Authors

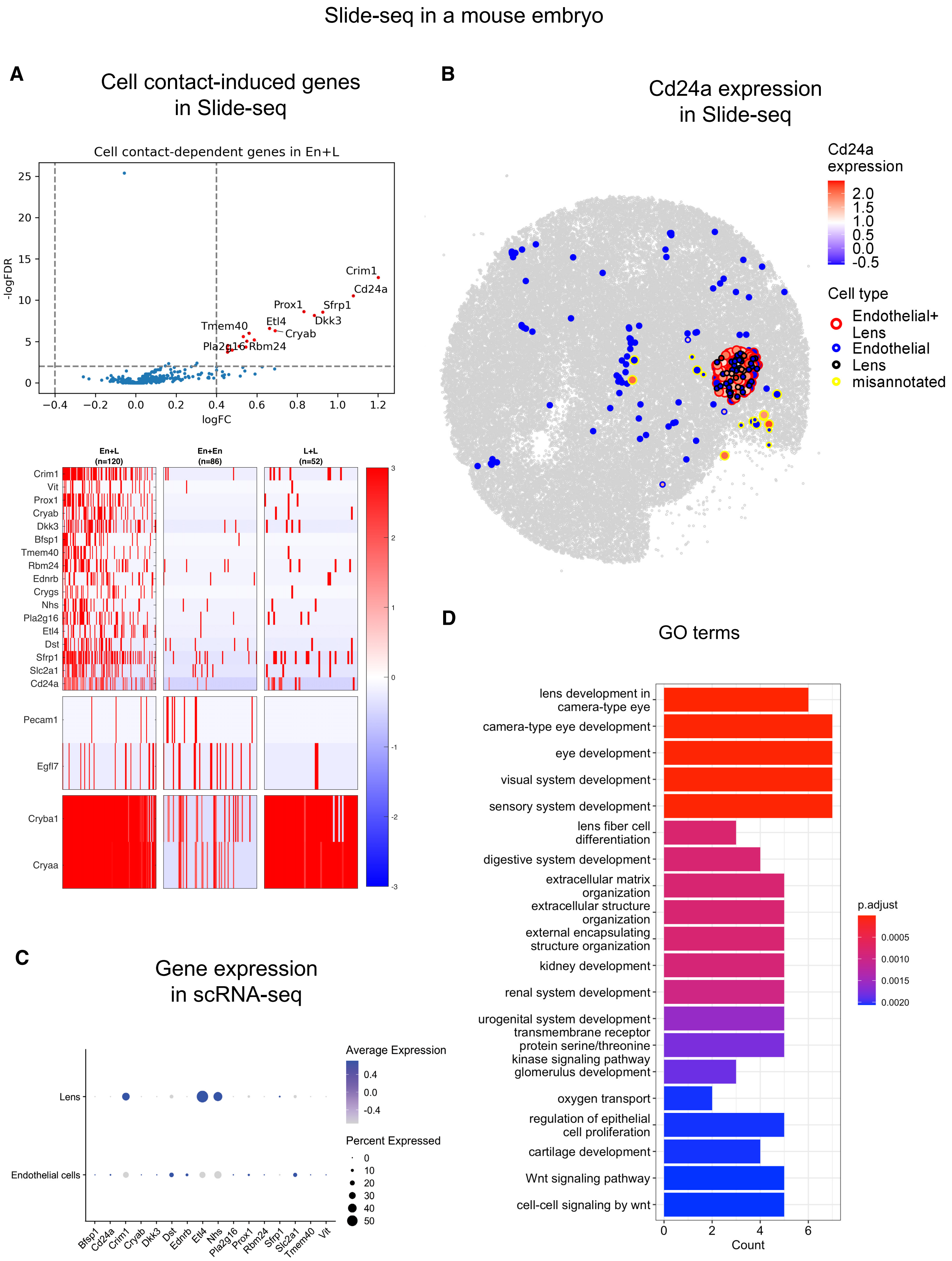

Figure EV2.

© 2023 The Authors

Figure EV3. CellNeighborEx identified genes influenced by TME.

A The heterotypic spots of Tumor III and Monocyte cells (Tumor III + Monocyte; $n = 742$) were compared with the respective homotypic spots (Tumor III [$n = 4{,}206$], Monocyte [$n = 218$]; log ratio > 0.4, P-value < 0.01) and additionally with the artificial heterotypic spots (FDR < 0.01). The statistical test was chosen among the two tailed Student's t-test, Welch's t-test, or Wilcoxon rank sum test depending on the sample size and heterogeneity of variance test. The volcano plot displays that *F13a1* is an up-regulated gene. In the heatmap, *F13a1* is more highly expressed in Tumor III + Monocyte. The heterotypic spots also express both Tumor III and Monocyte markers.

B The spatial visualization shows the higher expression level of *F13a1* in Tumor III + Monocyte.

C In the mouse liver cancer snRNA-seq data, it was confirmed that *F13a1* is mostly expressed in Monocyte cells.

D GO analysis in the mouse liver cancer data shows the terms associated with tumor metastasis. The bar plot presents top 20 GO terms.

© 2023 The Authors

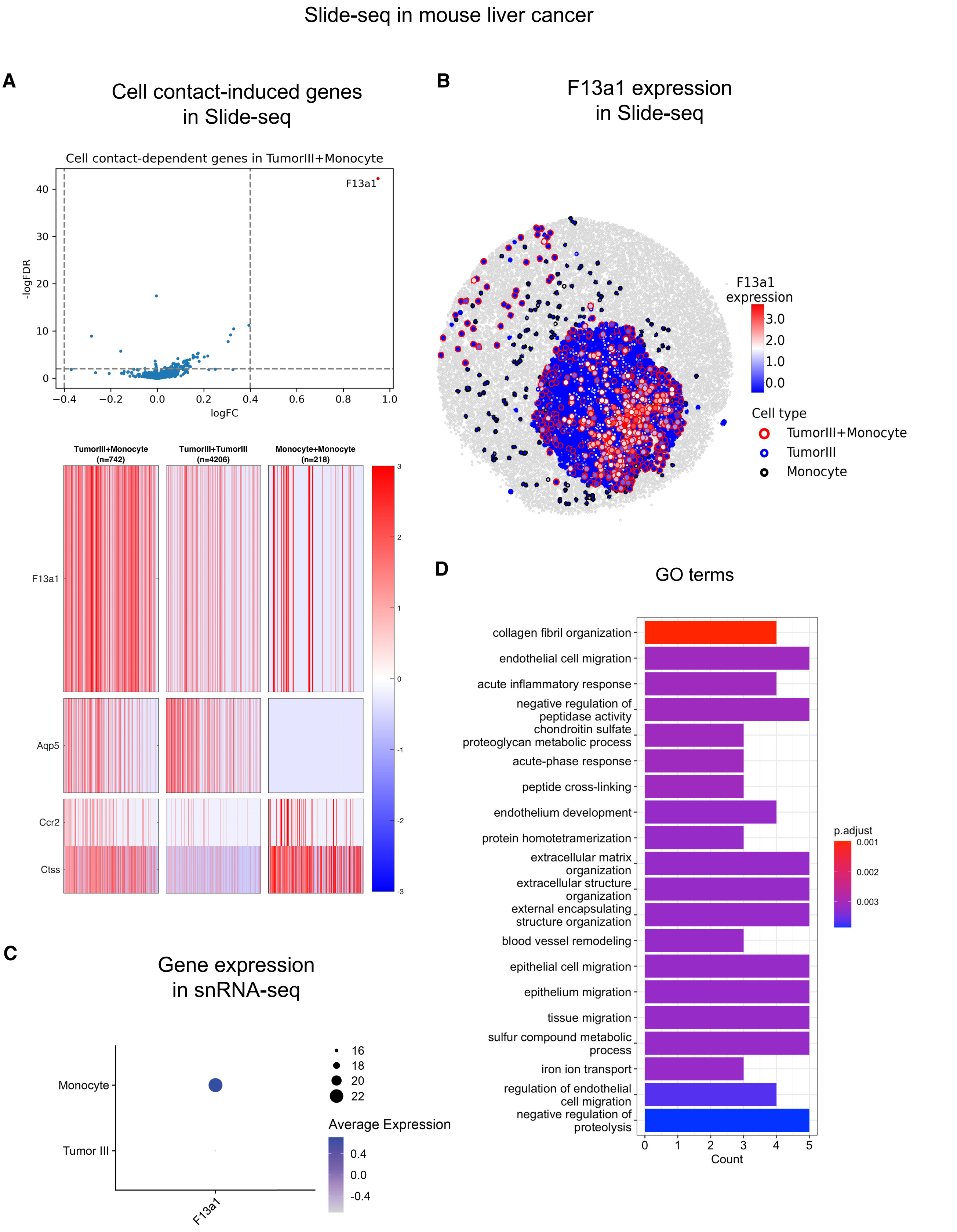

Figure EV3.

 © 2023 The Authors

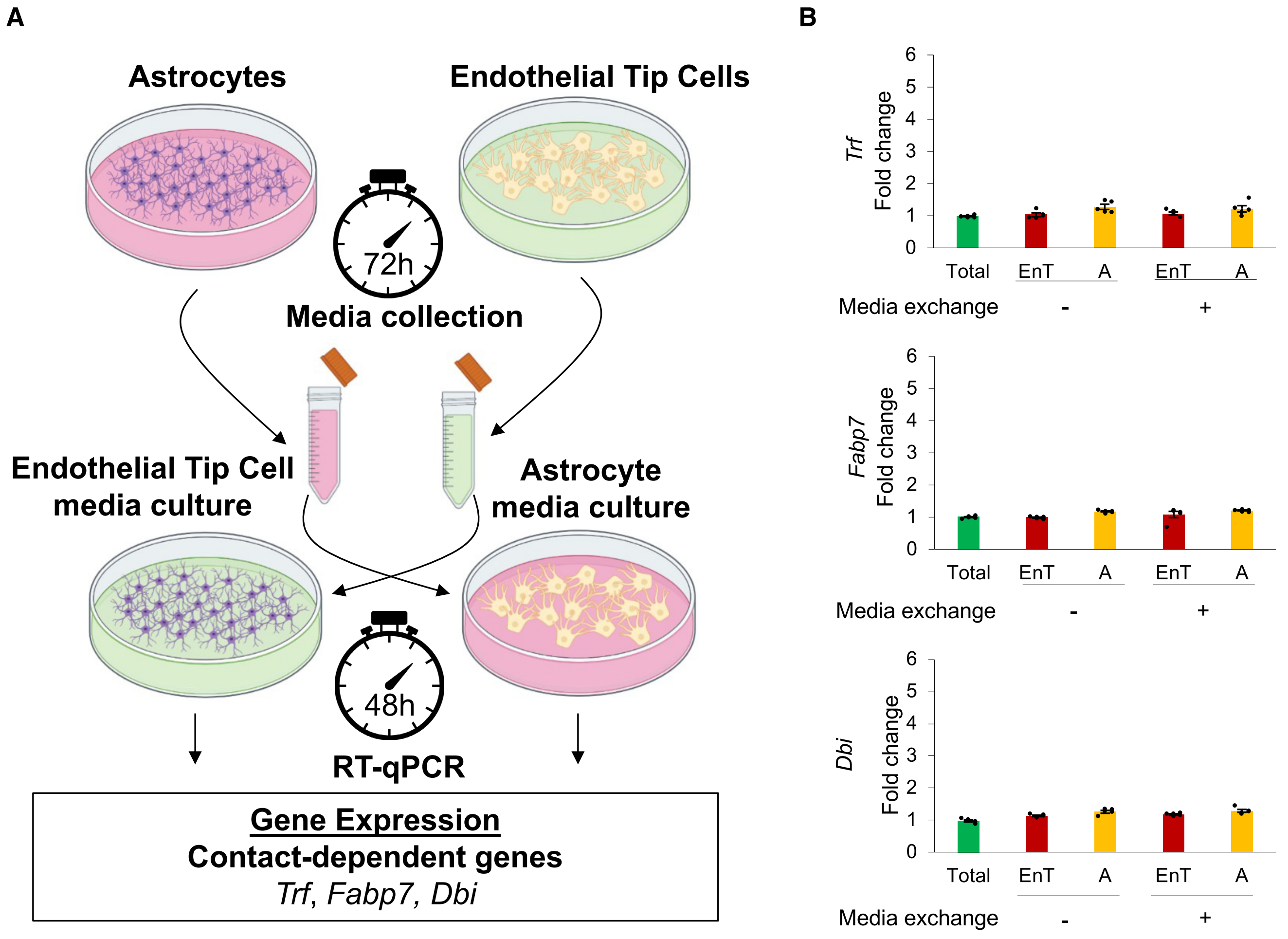

Figure EV4. Validation of neighbor-dependent gene expression upon media exchange of cells derived from mouse hippocampus.

A Model depicting the experimental methodology applied to analyze the expression of the predicted neighbor-dependent genes in the mouse hippocampus upon the exchange of cell-derived medias. Briefly, the isolated Astrocyte and Endothelial Tip (EnT) cells from mouse hippocampus were cultured separately for 72 h. Then, the media derived from Astrocyte was transferred to culture EnT cells, while the media from EnT cells was used to culture Astrocyte for 48 h. The cells were harvested and used to perform RT–qPCR. This model validates the expression of the predicted neighbor-dependent genes *Trf*, *Fabp7*, and *Dbi*.

B The analysis of qPCR-based mRNA expression of Astrocyte (red bars) and EnT cells (yellow bars) upon the media exchange shows no statistically significant differences between the expression of the predicted neighbor-dependent genes in Astrocyte and EnT cells before and after the media exchange. The expression of these genes in total mouse hippocampus represents the control (green bars). The expression of the predicted neighbor-dependent genes was normalized for the expression of these genes in total mouse hippocampus ($N = 5$; bars represent averaged fold changes of gene expression relative to the control; error bars indicate mean ± SE). The expression of all genes follows a normal distribution, which was calculated by the Shapiro–Wilk test and the F-test was performed to study the equality of variances. The P-values shown in the bar plot were obtained by performing the two-tailed Student's t-test with a confidence interval of 95%.

Source data are available online for this figure.

Figure EV5. Heterogeneity of endothelial tip (EnT) cells in mouse hippocampus Slide-seq data.

A Neighboring cell type-dependent gene expression of EnT cells. EnT cells dominantly express *Igfbp7* (red) when proximal to Choroid (EnT + Ch), *Trf* (green) when proximal to Astrocyte (EnT + A), and *Plp1* (blue) when proximal to Interneuron (EnT + In).

B Expression of neighbor-dependent genes in the mouse hippocampus scRNA-seq data. It confirms that the three genes are expressed from EnT.

C UMAP of EnT cells. 4 clusters were obtained through clustering analysis: Cluster 0 to 3. *Igfbp7* is mostly expressed in Cluster 3, *Trf* in Cluster 0, *Plp1* in Cluster 2, and none of them is expressed in Cluster 1.

D Heterogeneity of EnT cells explained by niche-specific gene expression. Cluster 3 is EnT cells adjacent to Ch, Cluster 0 is EnT adjacent to A, Cluster 2 is EnT adjacent to In, and Cluster 0 is EnT adjacent to another EnT.

© 2023 The Authors

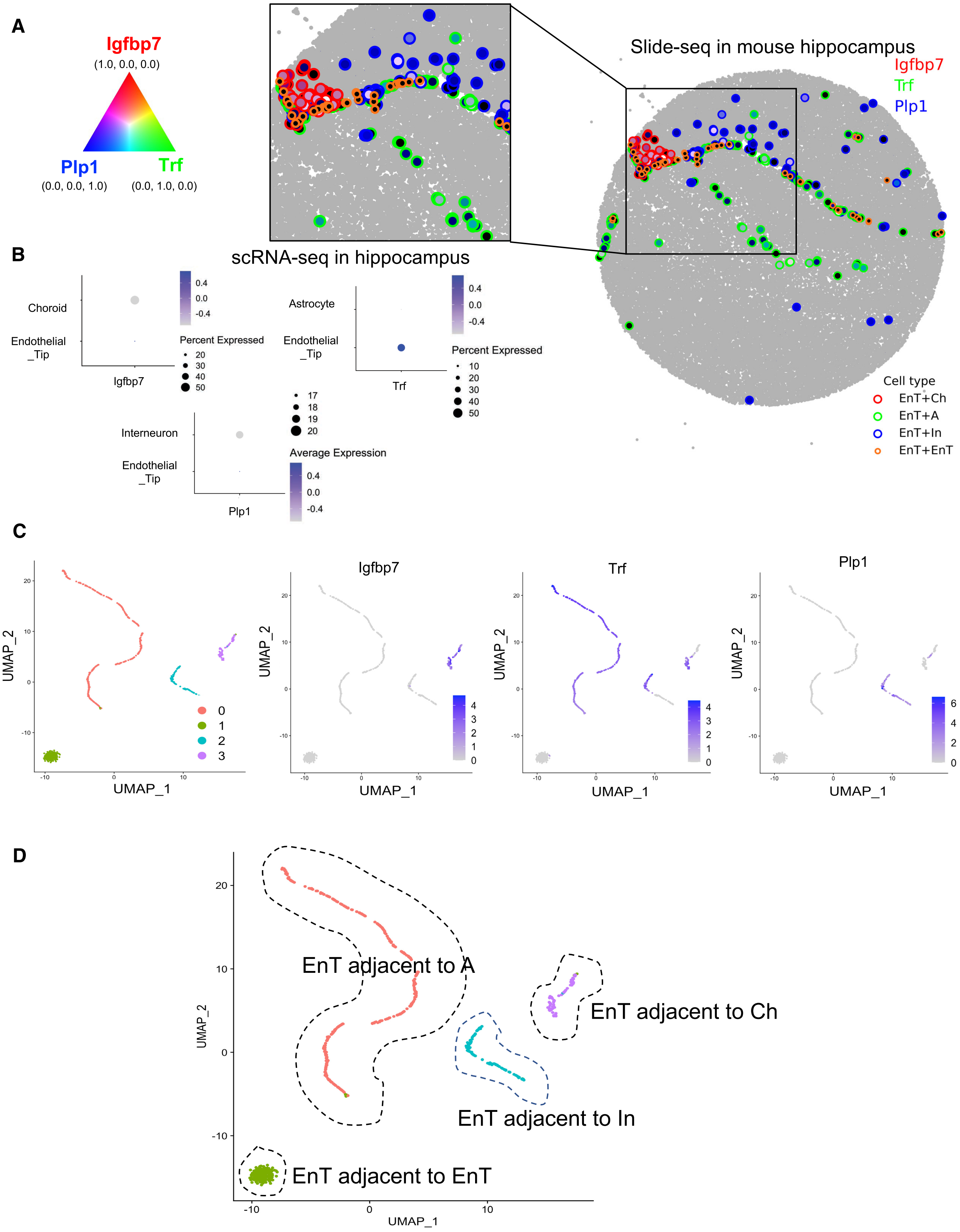

Figure EV5.

 © 2023 The Authors
