# Supplementary material for: CellNeighborEX: deciphering neighbor‐dependent gene expression from spatial transcriptomics data
Source: Mol Syst Biol. 2023 Oct 10;19(11):e11670. doi: 10.15252/msb.202311670 (PMC10632736; doi:10.15252/msb.202311670)
Supplement: Supplementary file 11 [file MSB-19-e11670-s007.pdf]

CellNeighborEX: Deciphering Neighbor-Dependent Gene Expression from Spatial Transcriptomics Data

Hyobin Kim, Amit Kumar, Cecilia Lövkvist, António Palma, Patrick Martin, Junil Kim, Praveen Bhoopathi, Jose Trevino, Paul Fisher, Esha Madan, Rajan Gogna, and Kyoung Won
DOI: 10.15252/msb.202311670

Corresponding author(s): Kyoung Won (kyoungjae.won@cshs.org) , Rajan Gogna (rajangogna@gmail.com)

Review Timeline:

Submission Date:	23rd Mar 23
Editorial Decision:	24th Apr 23
Revision Received:	21st Jul 23
Editorial Decision:	16th Aug 23
Revision Received:	14th Sep 23
Accepted:	18th Sep 23

Editor: Maria Polychronidou

Transaction Report:

(Note: With the exception of the correction of typographical or spelling errors that could be a source of ambiguity, letters and reports are not edited. Depending on transfer agreements, referee reports obtained elsewhere may or may not be included in this compilation. Referee reports are anonymous unless the Referee chooses to sign their reports.)

24th Apr 2023

Manuscript Number: MSB-2023-11670, CellNeighborEX: Deciphering Neighbor-Dependent Gene Expression from Spatial Transcriptomics Data

Dear Dr Won,

Thank you again for submitting your work to Molecular Systems Biology. We have now heard back from the three reviewers who agreed to evaluate your study. As you will see below, the reviewers appreciate that the proposed approach addresses a timely topic. However, they raise a series of concerns, which we would ask you to address in a major revision.

Without repeating all the issues listed below, some of the more fundamental issues raised are the following:

- Additional analyses related to the neighbor cells considered by CellNeighborEX and how their choice affects the results need to be included. The reviewers provide constructive comments related to this point.
- Further validations better supporting the biological relevance of the findings need to be performed.
- CellNeighborEX needs to be provided as a package that can be easily installed and used by future users and can be combined with existing workflows. A guide on how to use it would help make CellNeighborEX easily accessible.
- Related to the point above, the method should be described in better detail.

All issues raised by the reviewers would need to be satisfactorily addressed. Please let me know in case you would like to discuss in further detail any of the issues raised, I would be happy to schedule a call.

On a more editorial level, we would ask you to address the following points:

- Please provide a .doc version of the manuscript text (including legends for main figures) and individual production quality figure files for the main Figures (one file per figure).

- We have replaced Supplementary Information by the Expanded View (EV format). In this case, all additional figures can be included in a PDF called Appendix. Appendix figures should be labeled and called out as: "Appendix Figure S1, Appendix Figure S2... Appendix Table S1..." etc. Each legend should be below the corresponding Figure/Table in the Appendix. Please include a Table of Contents in the beginning of the Appendix. For detailed instructions regarding expanded view please refer to our Author Guidelines: .

- Supplementary Tables S1-S5 should be provided as EV Tables (less complex tables, not longer than one page) or EV Datasets (more complex tables with multiple sheets and longer than one page). Please provide one file per EV Table/Dataset. In each file, a description of the table/dataset should be provided in a separate tab. Please note that we do NOT allow multiple tables with the same number (e.g. EV1A, EV1B, EV1C), so the current numbering/labeling needs to be corrected!

- Please provide a "standfirst text" summarizing the study in one or two sentences (approximately 250 characters), three to four "bullet points" highlighting the main findings and a "synopsis image" (550px width and max 400px height, jpeg format) to highlight the paper on our homepage.

- All Materials and Methods need to be described in the main text. We would ask you to use 'Structured Methods', our new Materials and Methods format, which is mandatory for Methods and Articles with a strong methodological focus. According to this format, the Material and Methods section should include a Reagents and Tools Table (listing key reagents, experimental models, software and relevant equipment and including their sources and relevant identifiers) followed by a Methods and Protocols section in which we encourage the authors to describe their methods using a step-by-step protocol format with bullet points, to facilitate the adoption of the methodologies across labs. More information on how to adhere to this format as well as downloadable templates (.doc or .xls) for the Reagents and Tools Table can be found in our author guidelines: . An example of a Method paper with Structured Methods can be found here: .

- Please include a Data availability section describing how the data and code have been made available. This section needs to be formatted according to the example below:
The datasets and computer code produced in this study are available in the following databases:
- Chip-Seq data: Gene Expression Omnibus GSE46748 (https://www.ncbi.nlm.nih.gov/geo/query/acc.cgi?acc=GSE46748)
- Modeling computer scripts: GitHub (https://github.com/SysBioChalmers/GECKO/releases/tag/v1.0)
- [data type]: [full name of the resource] [accession number/identifier] ([doi or URL or identifiers.org/DATABASE:ACCESSION])

- For data quantification: please specify the name of the statistical test used to generate error bars and P values, the number (n) of independent experiments (specify technical or biological replicates) underlying each data point and the test used to calculate

p-values in each figure legend. The figure legends should contain a basic description of n, P and the test applied. Graphs must include a description of the bars and the error bars (s.d., s.e.m.).

- Please include a "Disclosure & Competing Interests Statement" in the main text.

- Molecular Systems Biology supports formal data citations in the Reference list, to cite previously published datasets. In addition to citing the original papers that reported the data, we encourage you to also cite the relevant datasets directly in the Reference list. In the text, references to datasets are included as "Data ref: Smith et al, 2001" or "Data ref: NCBI Sequence Read Archive PRJNA342805, 2017". In the Reference list, data citations are very similar to normal literature references but must be labeled with "[DATASET]" at the end of the reference. For detailed instructions please refer to our Author Guidelines .

- In the References, some author lists are truncated, please correct. (According to the journal's reference style the references should be ordered alphabetically, listing 10 author names followed by et al.)

- When you resubmit your manuscript, please download our CHECKLIST (https://bit.ly/EMBOPressAuthorChecklist) and include the completed form in your submission.
Please note that the Author Checklist will be published alongside the paper as part of the transparent process (https://www.embopress.org/page/journal/17444292/authorguide#transparentprocess).

If you feel you can satisfactorily deal with these points and those listed by the referees, you may wish to submit a revised version of your manuscript. Please attach a covering letter giving details of the way in which you have handled each of the points raised by the referees. A revised manuscript will be once again subject to review and you probably understand that we can give you no guarantee at this stage that the eventual outcome will be favorable.

Kind regards,

Maria

Maria Polychronidou, PhD
Senior Editor
Molecular Systems Biology

We realize that it is difficult to revise to a specific deadline. In the interest of protecting the conceptual advance provided by the work, we recommend a revision within 3 months (23rd Jul 2023). Please discuss the revision progress ahead of this time with the editor if you require more time to complete the revisions. Use the link below to submit your revision:

https://msb.msubmit.net/cgi-bin/main.plex

IMPORTANT: When you send your revision, we will require the following items:
1. the manuscript text in LaTeX, RTF or MS Word format
2. a letter with a detailed description of the changes made in response to the referees. Please specify clearly the exact places in the text (pages and paragraphs) where each change has been made in response to each specific comment given
3. three to four 'bullet points' highlighting the main findings of your study
4. a short 'blurb' text summarizing in two sentences the study (max. 250 characters)
5. a 'thumbnail image' (550px width and max 400px height, Illustrator, PowerPoint or jpeg format), which can be used as 'visual title' for the synopsis section of your paper.
6. Please include an author contributions statement after the Acknowledgements section (see https://www.embopress.org/page/journal/17444292/authorguide)
7. Please complete the CHECKLIST available at (https://bit.ly/EMBOPressAuthorChecklist).
Please note that the Author Checklist will be published alongside the paper as part of the transparent process (https://www.embopress.org/page/journal/17444292/authorguide#transparentprocess).
8. When assembling figures, please refer to our figure preparation guideline in order to ensure proper formatting and readability in print as well as on screen:
https://bit.ly/EMBOPressFigurePreparationGuideline
See also figure legend guidelines: https://www.embopress.org/page/journal/17444292/authorguide#figureformat
9. Please note that corresponding authors are required to supply an ORCID ID for their name upon submission of a revised manuscript (EMBO Press signed a joint statement to encourage ORCID adoption).
(https://www.embopress.org/page/journal/17444292/authorguide#editorialprocess)
Currently, our records indicate that the ORCID for your account is 0000-0002-2924-9630.

Please click the link below to modify this ORCID:
Link Not Available

10. At EMBO Press we ask authors to provide source data for the main manuscript figures. Our source data coordinator will contact you to discuss which figure panels we would need source data for and will also provide you with helpful tips on how to upload and organize the files.

The system will prompt you to fill in your funding and payment information. This will allow Wiley to send you a quote for the article processing charge (APC) in case of acceptance. This quote takes into account any reduction or fee waivers that you may be eligible for. Authors do not need to pay any fees before their manuscript is accepted and transferred to the publisher.

EMBO Press participates in many Publish and Read agreements that allow authors to publish Open Access with reduced/no publication charges. Check your eligibility: https://authorservices.wiley.com/author-resources/Journal-Authors/open-access/affiliation-policies-payments/index.html

As a matter of course, please make sure that you have correctly followed the instructions for authors as given on the submission website.

*** PLEASE NOTE *** As part of the EMBO Press transparent editorial process initiative (see our Editorial at https://dx.doi.org/10.1038/msb.2010.72), Molecular Systems Biology publishes online a Review Process File with each accepted manuscripts. This file will be published in conjunction with your paper and will include the anonymous referee reports, your point-by-point response and all pertinent correspondence relating to the manuscript. If you do NOT want this File to be published, please inform the editorial office at msb@embo.org within 14 days upon receipt of the present letter.

Reviewer #1:

Here, Kim et al present CellNeighborEX, a computational method for inferring neighbor-dependent gene expression from spatial transcriptomics data. Spatial transcriptomics data is becoming widely available, but there is still a need for computational methods to allow researchers to extract biological insights. One of the most active areas of research is to develop methods for inferring cell-cell interactions. The method presented here provides an interesting take on the problem by focusing on the role of nearby cells. This approach could be of interest to the community, but before publication the following major issues need to be addressed:

1 For the Delaunay triangulation, CellNeighborEX only considers the closest neighbors. However, in very dense regions, the non-closest neighbors may actually be closer than the closest one in a sparse region. Have the authors considered combining the nearest-neighbor criterion with a distance based one?

2 On p5 the authors claim that neighbors can be defined by Delaunay triangulation, k-nearest or radial distance. However, as far as I can tell, only the Delaunay triangulation is used in the manuscript for the seqFISH data. As the latter two approaches require one or more parameters, I think that some results and guidelines for these approaches need to be included. Otherwise this functionality is not of much use.

3 The authors highlight some key interacting genes (Pitx1, Cd24). However, the support in the literature seems weak or non-existing and this makes me a bit worried. I would be more convinced about the validity of the results if there was better evidence supporting the findings from the literature.

4 The validation experiment in Fig 3e is valuable, but the setup seems a bit strange. The analysis was carried out for mouse hippocampus, yet the qPCR data is normalized by the expression from the total brain. Would it not make more sense to use the whole hippocampus to normalize? I agree that the increased expression in the co-culture is consistent with the predictions of their model, but it seems as if there could be other factors than proximity that matter. One of the endothelial cells could be secreting ligands that stimulate Fabp7 over longer distances and this has not been controlled for.

5 Throughout their use-cases, the authors highlight several genes. However, it is difficult to put these results into context since the global picture is not provided. For example, it would be helpful if Fig 2 to show a volcano plot, heatmap or other representation including other genes to be able to compare Pitx1 and understand if it is representative or an extreme case. The same goes for Figs 3-5.

6 The number of DE genes varies a lot between datasets. It would be helpful if the authors could discuss as to what extent these differences are technical and due to the choice of assay or if it reflects the underlying biology.

7 The github page is as far as I can tell essentially just a collection of scripts. If someone else wanted to use this method, it would be quite challenging. To make CellNeighborEX more useful to the rest of the community, the authors should make it available as a package that can be used with existing workflows, e.g. scanpy. With just a collection of poorly documented scripts, the barrier

for using this method is high.

Reviewer #2:

Summary:
The authors propose a method, CellNeighborEX, to identify genes whose expression levels are affected by neighboring cells using spatial-transcriptome data. They utilize both image-based spatial transcriptomics (ST) data and next-generation sequencing (NGS)-based data to demonstrate the versatility of their approach. By classifying cells into homotypic and heterotypic neighbors, they successfully identify differentially expressed genes (DEGs) between these groups and validate their findings using single-cell RNA sequencing (scRNA-seq) data with niche-specific gene expressions.

General remarks:
Understanding gene expression changes within cells when in contact with specific cell types, known as niches, is crucial for comprehending cell development and homeostasis. However, single-cell and single-nucleus omics analysis often lack spatial information, except for partially sort-seq. Spatial-transcriptomics techniques, such as image-based, NGS-based, and in situ sequencing, provide valuable insights into cell biology. However, image-based approaches are limited in the number of genes they can cover, and NGS-based approaches suffer from low resolution. Despite these limitations, CellNeighborEX effectively identifies neighbor-dependent genes and confirms them with another platform. Although CellNeighborEX may not be perfect for 3D tissue characteristics, this paper serves as a promising starting point for future research.

As mentioned above, this manuscript is suitable for publication, but I would like to suggest some revision points to enhance its impact and citation potential.

Major points:
1. In NGS-based ST data, spots that contain heterogeneous cells may consist of three or four types of cells, such as A, A' (neighboring B), B, or B' (neighboring A), rather than just A+B. Have you considered the possibility of changes in the membership of neighbor-dependent genes in your analytical pipeline when accounting for this scenario?
2. Why have you not discarded homotypic spots located near heterotypic spots in NGS-based ST data? It is possible that some A cells stochastically contact B cells.
3. Have you checked the consistency of results from subsequent analyses based on newly identified gene sets when you change the threshold for classifying homotypic and heterotypic neighbors? For example, A+A spots far from A+B spots may represent genuine A+A interactions, while A+A spots nearby A+B spots may be influenced by A' cells, which are A cells with trait changes due to neighboring B cells.
4. Considering the situation in 3D tissue and the potential for changes in cell classification, you may want to order the same cells based on the expression levels of neighbor-dependent genes. Can we predict the diversity of neighbors (z-axis in different slides) in reverse based on the results obtained?

Minor points:
1. Have you considered using Shannon's entropy as an alternative method to measure heterogeneity, which is used to classify cells as heterotypic or homotypic neighbors?
2. Since the number of interesting genes is small in image-based ST data, and sample size may affect the normality of expression distribution, it would be advisable to conduct normality testing and use parametric statistical tests. In method part, the authors tried to convince relationship between size and normality but they may use normality test directly.
3. NGS-based ST data, like scRNA-seq data, often contains many zero values. Have you considered using a zero-inflated negative binomial (NB) distribution to account for expression distribution?
4. It may be challenging to understand the analytic pipeline from the GitHub repository. As a tool, it would be helpful to make it more user-friendly guides and easily accessible for users.
5. How did you select the Gene Ontology (GO) terms associated with embryonic development in Fig. 2C and those related to neuronal regulation in Fig. 3D? Please provide more details on the selection process.

Reviewer #3:

Kim et al develop CellNeighborEX: a novel computational approach to identify differential patterns of gene expression in spatial transcriptomics datasets by comparing the transcriptome of heterotypic vs homotypic neighbors.

This is an interesting and busy area of research with many groups developing methods for understanding cell-cell communication using spatial methods. However, many existing methods employ considerable prior knowledge about cell receptor and ligand expression and interaction which may not generalize well and ignore information contained in the whole

transcriptome. From this standpoint the present work stands out as being considerably simpler and with less assumptions.

The paper however, has a few point that could use improvement:

- Since the method depends heavily on cell segmentation and cell type annotation, I do wonder about the contribution of errors during pre-processing to the outputs of CellNeighborEX. For example, differential expression between two very related cell states of a related cell type when annotated differently could yield false positives

- The observation that Fabp7 is upregulated during co-culture of astrocytes and endothelial cells is a commendable effort towards the experimental validation of the outputs, but unfortunately falls short of a comprehensive validation of the method. The authors found for example 3 differentially expressed genes in this pair of neighbors - why not do PCR for all 3?

- My intuition is that by using homotypic cell interactions as control, the statistical testing is a bit more controlled than other methods comparing expression of single-cells, despite the authors using simply a T-test. However, demonstrating this with for example M-A and volcano plots would go a great way to showcase that the statistical expectations of the test are met.

- I compliment the authors on making code and datasets publicly available.
However, the software made available is not quite up to the expectations of modern scientific software production and reproducibility of the results.
The authors should specify how to install the required software (conda/pip install, etc), scripts used to reproduce the results obtained in the paper and documentation on how to run them. Furthermore, for example jupyter notebooks integrating both analysis and visualizations, as well as a pip installable package that can be run from the command-line interface or within python interactively would be invaluable to new users wanting to run CellNeighborEX on their data.

- Illustration of data: it would benefit greatly the reader if the authors provided either matched/paired images of the tissues for a better appreciation of the anatomical context. In the case of it not being available, raw transcript intensity may also help here. In particular, it is very hard to grasp some of the interactions the authors point to in the examples due to the representation of cells as circles with both filled and outline color. Perhaps panels dedicated to illustrate either expression only or cell type identity only (as in Figure 4) would help.

- Representation of single-cell expression values as dot-plots (eg. Figure 5b, d, f) is not maximizing the information content per plot area or illustrating the distribution of values across cells. Perhaps bar, violin, or swarmplots would be better.

- From a reading perspective, the authors spend very little time describing the method itself in the Results section. Given it's a methods paper, providing more detail in the main text might be better. The current form of applying method to various datasets sometimes without a particular motivation is rather repetitive.

Reviewer #1:

Here, Kim et al present CellNeighborEX, a computational method for inferring neighbor-dependent gene expression from spatial transcriptomics data. Spatial transcriptomics data is becoming widely available, but there is still a need for computational methods to allow researchers to extract biological insights. One of the most active areas of research is to develop methods for inferring cell-cell interactions. The method presented here provides an interesting take on the problem by focusing on the role of nearby cells. This approach could be of interest to the community, but before publication the following major issues need to be addressed:

Thanks a lot for constructive comments and finding scientific merits from our manuscript. Please find the point-to-point responses.

1. For the Delaunay triangulation, CellNeighborEX only considers the closest neighbors. However, in very dense regions, the non-closest neighbors may actually be closer than the closest one in a sparse region. Have the authors considered combining the nearest-neighbor criterion with a distance based one?

Answer: As the reviewer said, the non-closest neighbors in a dense region can be closer than the closest ones in a sparse region in terms of distance. Delaunay triangulation can be used alone as well as in combination with radial distance. We made it clear in the manuscript in the first paragraph of "CellNeighborEX categorizes transcriptome to investigate the influence of neighboring cell types" on p.5: *"Delaunay triangulation can be used alone or in combination with radial distance"*.

2. On p5 the authors claim that neighbors can be defined by Delaunay triangulation, k-nearest or radial distance. However, as far as I can tell, only the Delaunay triangulation is used in the manuscript for the seqFISH data. As the latter two approaches require one or more parameters, I think that some results and guidelines for these approaches need to be included. Otherwise, this functionality is not of much use.

Answer: Following the reviewer's comments, we have provided some guidelines for the two approaches in the first paragraph of "CellNeighborEX categorizes transcriptome to investigate the influence of neighboring cell types" on p.5. Specifically, we added *"Both radial distance and KNN require prior knowledge. KNN is useful for cells evenly distributed on a tissue. Radial distance will be useful if we know the distance between the cells of interest. Delaunay triangulation can be used alone or in*

combination with radial distance.". In addition, we have prepared a function *calculate_closest_distance* to provide the distribution of the closest cell-cell distances in our package to assist it.

3. The authors highlight some key interacting genes (*Pitx1*, *Cd24*). However, the support in the literature seems weak or non-existing and this makes me a bit worried. I would be more convinced about the validity of the results if there was better evidence supporting the findings from the literature.

Answer: It is true that it is not easy to find supporting literatures as there are not enough study to discuss cell contact-dependent genes. We tried our best to provide additional literatures for the genes we identified. We mentioned the literatures for Pitx1 in the second paragraph on p.7 and for Cd24a in the third paragraph on p.8. However, we could not find exactly matching cases explaining cell contact-induced expression for these genes.

We previously showed that cell contact can induce gene expression in the mouse embryo (Kim et al. 2023, PNAS). In this manuscript, we also validated that genes can be induced by cell contact in the mouse adult tissue as well (Fig EV4). In addition, we prepared various visualization on spatial transcriptomics data to show the interacting genes and the associated cell types. These all show the validity of neighbor-dependent gene expression.

4. The validation experiment in Fig 3e is valuable, but the setup seems a bit strange. The analysis was carried out for mouse hippocampus, yet the qPCR data is normalized by the expression from the total brain. Would it not make more sense to use the whole hippocampus to normalize? I agree that the increased expression in the co-culture is consistent with the predictions of their model, but it seems as if there could be other factors than proximity that matter. One of the endothelial cells could be secreting ligands that stimulate *Fabp7* over longer distances and this has not been controlled for.

Answer: Following the reviewer's suggestion, we normalized the expression against the whole hippocampus. To show that the differences observed in the expression of our tested genes was due to direct cell contact and not through the release of cellular factors, we performed a media exchange assay in which we cultured Endothelial Tip (EnT) cells with Astrocyte (As)-derived media and cultured As with EnT cells-derived media for 48h. Then, we analyzed the expression of *Trf*, *Fabp7,* and *Dbi* in these cells. In these conditions, we did not observe significant changes in the expression of these genes compared to their expression in the mouse hippocampus when As and EnT cells are cultured in their respective media. These results suggests that the induction of de novo gene expression results from the communication established by these two cell types. We gave the description of this experiment in the third and fourth

paragraphs of "Neighbor-dependent genes are discovered from Slide-seq data in mouse brain" on pp.10-11. We added Figure 3, and EV4.

5. Throughout their use-cases, the authors highlight several genes. However, it is difficult to put these results into context since the global picture is not provided. For example, it would be helpful if Fig 2 to show a volcano plot, heatmap or other representation including other genes to be able to compare Pitx1 and understand if it is representative or an extreme case. The same goes for Figs 3-5.

Answer: Thanks for the suggestions. We have presented volcano plots whenever it is appropriate (Fig 2A, Fig EV1A, EV2A, and EV3A).

6. The number of DE genes varies a lot between datasets. It would be helpful if the authors could discuss as to what extent these differences are technical and due to the choice of assay or if it reflects the underlying biology.

Answer: The difference of the number of DEGs can be due to technical and/or biological variations. Image-based approach provides high sensitivity but the number of detectable genes is limited. This greatly influenced the number of DEGs. It is not easy to explain technical variation. To measure how much technical variation is, we need datasets from seqFISH and Slide-seq applied to the same biopsy. For biological variation, many datasets for multiple biological replicates are required on the same technology. Based on such datasets, it would be possible to determine the causes of variations.

We added that *"The number of neighbor-dependent genes can vary across the datasets. Image-based approaches such as seqFISH provide more accurate measurement of RNA quantity against NGS-based ones, which can allow more statistical power in detecting neighbor dependent-genes. However, the number of detectable genes is limited in the image-based approaches, making it hard to detect unknown genes influenced by neighbors."* in the second paragraph on p.15.

7. The github page is as far as I can tell essentially just a collection of scripts. If someone else wanted to use this method, it would be quite challenging. To make CellNeighborEX more useful to the rest of the community, the authors should make it available as a package that can be used with existing workflows, e.g., scanpy. With just a collection of poorly documented scripts, the barrier for using this method is high.

Answer: We have created the Python package (https://github.com/hkim240/CellContact).

Reviewer #2:

Summary:
The authors propose a method, CellNeighborEX, to identify genes whose expression levels are affected by neighboring cells using spatial-transcriptome data. They utilize both image-based spatial transcriptomics (ST) data and next-generation sequencing (NGS)-based data to demonstrate the versatility of their approach. By classifying cells into homotypic and heterotypic neighbors, they successfully identify differentially expressed genes (DEGs) between these groups and validate their findings using single-cell RNA sequencing (scRNA-seq) data with niche-specific gene expressions.

General remarks:
Understanding gene expression changes within cells when in contact with specific cell types, known as niches, is crucial for comprehending cell development and homeostasis. However, single-cell and single-nucleus omics analysis often lack spatial information, except for partially sort-seq. Spatial-transcriptomics techniques, such as image-based, NGS-based, and in situ sequencing, provide valuable insights into cell biology. However, image-based approaches are limited in the number of genes they can cover, and NGS-based approaches suffer from low resolution. Despite these limitations, CellNeighborEX effectively identifies neighbor-dependent genes and confirms them with another platform. Although CellNeighborEX may not be perfect for 3D tissue characteristics, this paper serves as a promising starting point for future research.

As mentioned above, this manuscript is suitable for publication, but I would like to suggest some revision points to enhance its impact and citation potential.
Answer: Thanks a lot for finding scientific values in our manuscript. The followings are the answers for the comments.

Major points:
1. In NGS-based ST data, spots that contain heterogeneous cells may consist of three or four types of cells, such as A, A' (neighboring B), B, or B' (neighboring A), rather than just A+B. Have you considered the possibility of changes in the membership of neighbor-dependent genes in your analytical pipeline when accounting for this scenario?

Answer: As the reviewer pointed out, it can be possible that there exist A and B beside A' and B'. In this scenario, the expression of neighbor-specific genes in A' or B' can be diluted by the transcriptome of A and B, which can lower the expression levels of neighbor-specific genes. Even with potential dilution, the expression levels were strong enough to identify neighbor-specific genes. Moreover, considering that the spot size is 10 μm, we think that the influence of dilution is very limited.

2. Why have you not discarded homotypic spots located near heterotypic spots in NGS-based ST data? It is possible that some A cells stochastically contact B cells.

Answer: As the reviewer suggested, we left out homotypic spots adjacent to heterotypic spots in the Slide-seq datasets. Specifically, we discarded homotypic spots located within 30 μm from heterotypic spots. For each dataset, we identified new DEGs: 19 genes in the embryo, 154 genes in the hippocampus, and 60 genes in the liver cancer. We checked how many DEGs are conserved by comparing the new DEG list with the previous one. It was confirmed that about 80% of DEGs are consistent on average: 17 genes among 28 genes in the embryo, 138 genes among 155 genes in the hippocampus, and 39 genes among 42 genes in the liver cancer.

Discarding homotypic spot located near heterotypic spots can remove potential false negatives. At the same time, it can remove the homotypic ones not influenced by heterotypic spots, resulting in reduced statistical power. We think that it might be better to maintain our approach because our DEGs are genes robustly expressed even against the stochastic effects of heterotypic spots on homotypic spots.

3. Have you checked the consistency of results from subsequent analyses based on newly identified gene sets when you change the threshold for classifying homotypic and heterotypic neighbors? For example, A+A spots far from A+B spots may represent genuine A+A interactions, while A+A spots nearby A+B spots may be influenced by A' cells, which are A cells with trait changes due to neighboring B cells.

Answer: A' which is influence by B is still A with some changes in a small number of genes. If clustered, A' are well mixed with A. Therefore, the change of the threshold for classifying homotypic and heterotypic spots does not change the results. We also tested only using genuine A+A by removing A+A close to A+B (see the answers for comment #2) and obtained robust results.

4. Considering the situation in 3D tissue and the potential for changes in cell classification, you may want to order the same cells based on the expression levels of neighbor-dependent genes. Can we predict the diversity of neighbors (z-axis in different slides) in reverse based on the results obtained?

Answer: Yes, we can predict the diversity of neighbors. Instead of ordering them, we projected single cells onto UMAP space based on the expression values of neighbor dependent genes (Fig EV6 and EV7). We found separate clusters each with neighbor-dependent genes, explaining the heterogeneity caused by neighboring cells.

Minor points:

1. Have you considered using Shannon's entropy as an alternative method to measure heterogeneity, which is used to classify cells as heterotypic or homotypic neighbors?

Answer: We did not consider it. Without applying Shannon's entropy, it was possible to obtained them without too much trouble.

2. Since the number of interesting genes is small in image-based ST data, and sample size may affect the normality of expression distribution, it would be advisable to conduct normality testing and use parametric statistical tests. In method part, the authors tried to convince relationship between size and normality, but they may use normality test directly.

Answer: As the reviewer suggested, we have included an option to perform the normality test (i.e., Shapiro-Wilk test) and find differentially expressed genes according to the result in the package of CellNeighborEX. We briefly mentioned it in the first paragraph of "Neighbor-dependent gene expression analysis" on p.19.

3. NGS-based ST data, like scRNA-seq data, often contains many zero values. Have you considered using a zero-inflated negative binomial (NB) distribution to account for expression distribution?

Answer: We have not directly considered a zero-inflated negative binomial (ZINB) distribution. However, if gene expression values follow ZINB that is a non-normal distribution, the Wilcoxon rank sum test should be eventually performed in differential expression analysis. Also, this is the convention used by other scRNAseq packages such as Seurat to obtain DEGs.

4. It may be challenging to understand the analytic pipeline from the GitHub repository. As a tool, it would be helpful to make it more user-friendly guides and easily accessible for users.

Answer: We have created the Python package in the GitHub repository.
(https://github.com/hkim240/CellContact).

5. How did you select the Gene Ontology (GO) terms associated with embryonic development in Fig. 2C and those related to neuronal regulation in Fig. 3D? Please provide more details on the selection process.

Answer: We showed the top GO terms. We explained it clearly and added further details in the third paragraph on p.7.

Reviewer #3:

Kim et al develop CellNeighborEX: a novel computational approach to identify differential patterns of gene expression in spatial transcriptomics datasets by comparing the transcriptome of heterotypic vs homotypic neighbors.

This is an interesting and busy area of research with many groups developing methods for understanding cell-cell communication using spatial methods. However, many existing methods employ considerable prior knowledge about cell receptor and ligand expression and interaction which may not generalize well and ignore information contained in the whole transcriptome. From this standpoint the present work stands out as being considerably simpler and with less assumptions.

The paper, however, has a few points that could use improvement:

- Since the method depends heavily on cell segmentation and cell type annotation, I do wonder about the contribution of errors during pre-processing to the outputs of CellNeighborEX. For example, differential expression between two very related cell states of a related cell type when annotated differently could yield false positives.

Answer: We agree with that cell type annotation can heavily affect the identification of cell contact-dependent genes. Mis-annotation can bring some noises in obtaining DEGs and cause false positives. Inclusion of false positives can increase the positive samples without any neighbor specific gene expression. The increase in the number of false positives will undermine the statistical test result depending on the sample size. Studying the sample size against false positives has been discussed in statistics. Also, false positives can provide an effect similar to dropouts (detected expression level=0) in scRNAseq. Even in the presence of potential dropouts or false positives, we successfully identified neighbor dependent-genes.

We tried to control it by applying the false discovery rate (FDR) to both image and NGS based data. We also put a lot of work into the validation of cell type annotation to minimize yielding false positives. Concerning Slide-seq datasets, we double-checked whether the cell types are correctly annotated by confirming the expression of cell type markers and additionally calculating Pearson's correlation between true heterotypic spots and artificial heterotypic spots.

- The observation that Fabp7 is upregulated during co-culture of astrocytes and

endothelial cells is a commendable effort towards the experimental validation of the outputs, but unfortunately falls short of a comprehensive validation of the method. The authors found for example 3 differentially expressed genes in this pair of neighbors - why not do PCR for all 3?
Answer: Following the suggestion, we performed PCR for the three genes (Fig 3 and Fig EV4).

- My intuition is that by using homotypic cell interactions as control, the statistical testing is a bit more controlled than other methods comparing expression of single-cells, despite the authors using simply a T-test. However, demonstrating this with for example M-A and volcano plots would go a great way to showcase that the statistical expectations of the test are met.
Answer: As the reviewer suggested, we used volcano plots (Fig 2A, Fig EV1A, EV2A, and EV3A).

- I compliment the authors on making code and datasets publicly available. However, the software made available is not quite up to the expectations of modern scientific software production and reproducibility of the results. The authors should specify how to install the required software (conda/pip install, etc), scripts used to reproduce the results obtained in the paper and documentation on how to run them. Furthermore, for example jupyter notebooks integrating both analysis and visualizations, as well as a pip installable package that can be run from the command-line interface or within python interactively would be invaluable to new users wanting to run CellNeighborEX on their data.
Answer: We have created the Python package of CellNeighborEX and put it on Github. (https://github.com/hkim240/CellContact).

- Illustration of data: it would benefit greatly the reader if the authors provided either matched/paired images of the tissues for a better appreciation of the anatomical context. In the case of it not being available, raw transcript intensity may also help here. In particular, it is very hard to grasp some of the interactions the authors point to in the examples due to the representation of cells as circles with both filled and outline color. Perhaps panels dedicated to illustrating either expression only or cell type identity only (as in Figure 4) would help.
Answer: Unfortunately, their anatomical images are not available. It is also difficult to show anatomical regions clearly with raw transcript intensity because many spots of Slide-seq represent mixed

transcriptomes obtained from two different cell types. Instead, we have illustrated spatial plots with cell identities in the appendix figures (Fig S5).

- Representation of single-cell expression values as dot-plots (e.g., Figure 5b, d, f) is not maximizing the information content per plot area or illustrating the distribution of values across cells. Perhaps bar, violin, or swarm plots would be better.

Answer: To improve the representation of single cell or nucleus expression, we have included violin plots in the appendix figures (Fig S7).

- From a reading perspective, the authors spend very little time describing the method itself in the Results section. Given it's a methods paper, providing more detail in the main text might be better. The current form of applying method to various datasets sometimes without a particular motivation is rather repetitive.

Answer: We have described our methods in more detail in the main text. They are highlighted in blue.

16th Aug 2023

Manuscript Number: MSB-2023-11670R
Title: CellNeighborEX: Deciphering Neighbor-Dependent Gene Expression from Spatial Transcriptomics Data

Dear Dr Won,

Thank you for sending us your revised manuscript. We have now heard back from two of the three reviewers who agreed to evaluate your revised study. We have not yet heard back from reviewer #2, despite a couple of reminders. Since the other two reviewers make similar recommendations we think that in the interest of time we can proceed with making a decision based on the two available reports. As you will see below, reviewers #1 and #3 think that the study has improved as a result of the performed revisions. However, they still raise concerns regarding the data and software availability. Addressing these issues is very important for the eventual acceptance of the study for publication and we would ask you to fix them in a revision. Please make sure that CellNeighborEX is provided in a way that it is easy and intuitive for future users to install and use for analysing their data. The reviewers provide constructive comments in this regard. It might also be useful to consult with colleagues and ask for their feedback on whether they find the documentation, instructions etc. intuitive for external users.

We would also ask you to address some minor editorial issues listed below.

- Our data editors have noticed some unclear or missing information in the figure legends, please see the attached .doc file. We have also made some minor formatting edits. Please make all requested text changes using the attached file and *keeping the "track changes" mode* so that we can easily access the edits made.

- Please provide individual files (one file per figure) for the EV Figures. The EV Figure legends, should be included in the main manuscript text, below the main figure legends.

- There are callouts to Figures EV6 and EV7, it seems that these should be Appendix Figure S6 and S7? Please check and correct.

- Please include page numbers in the Appendix Table of Contents.

- The synopsis image should be provided the image in a .jpg or .png format. It needs to be exactly 550 px wide, and the height ideally < 500 px. Please make sure that all labels are readable at this final size.

Please resubmit your revised manuscript online, with a covering letter listing amendments and responses to each point raised by the referees. Please resubmit the paper **within one month** and ideally as soon as possible. If we do not receive the revised manuscript within this time period, the file might be closed and any subsequent resubmission would be treated as a new manuscript. Please use the Manuscript Number (above) in all correspondence.

Click on the link below to submit your revised paper.

https://msb.msubmit.net/cgi-bin/main.plex

Thank you for submitting this paper to Molecular Systems Biology.

Kind regards,

Maria

Maria Polychronidou, PhD
Senior Editor
Molecular Systems Biology

If you do choose to resubmit, please click on the link below to submit the revision online before 15th Sep 2023.

https://msb.msubmit.net/cgi-bin/main.plex

IMPORTANT:
Please note that corresponding authors are required to supply an ORCID ID for their name upon submission of a revised

manuscript (EMBO Press signed a joint statement to encourage ORCID adoption).
(https://www.embopress.org/page/journal/17444292/authorguide#editorialprocess)
Currently, our records indicate that the ORCID for your account is 0000-0002-2924-9630.

Please click the link below to modify this ORCID:
Link Not Available

The system will prompt you to fill in your funding and payment information. This will allow Wiley to send you a quote for the article processing charge (APC) in case of acceptance. This quote takes into account any reduction or fee waivers that you may be eligible for. Authors do not need to pay any fees before their manuscript is accepted and transferred to the publisher.

As a matter of course, please make sure that you have correctly followed the instructions for authors as given on the submission website.

*** PLEASE NOTE *** As part of the EMBO Press transparent editorial process initiative (see our Editorial at https://dx.doi.org/10.1038/msb.2010.72 , Molecular Systems Biology will publish online a Review Process File to accompany accepted manuscripts. When preparing your letter of response, please be aware that in the event of acceptance, your cover letter/point-by-point document will be included as part of this File, which will be available to the scientific community. More information about this initiative is available in our Instructions to Authors. If you have any questions about this initiative, please contact the editorial office (msb@embo.org).

Reviewer #1:

The authors have provided an improved version of their manuscript and all of my comments pertaining to the paper have been addressed. However, I am still not satisfied about the software package. I was able to install the python package without any issues. However, using it proved more challenging as I could not find any explicit instructions. I was able to find the scripts describing the analyses in the paper after some digging around in the repository. Although they are well structured and well commented, this is not a substitute for a documentation. Without more explicit instructions/vignette it will be challenging for others to use CelleighborEX. In 2023, it is to be expected that there are basic instructions on how to use a novel software package and this makes it much more valuable to the community.

Reviewer #3:

The authors have addressed all but one of my concerns. Unfortunately the data and software availability is still subpar and precluding both standard reproducibility practices and reducing the potential impact the paper may have in the community since without these features, it is impossible to run the software, assess its usability and evaluate its reproducibility and real-world use.

The authors should attend to the following specific points:
- Data:
- The github repository only includes data on simulated datasets, and thus missing most data that for the basis of the method validation (Slide-seq and seqFISH).
- The authors should distribute all datasets used in the manuscript, ideally as a separate, referenceable repository with persistent identifiers (e.g. Zenodo).
- Documentation: No documentation is provided beyond installation of the software. The authors should provide instructions on how to use their software to enable usage and adoption of the software. Specific points required:
- Basic usage (if possible including a demo/toy dataset)
- Information on what are the types of input for CellNeighborEX (e.g. h5ad), what assumptions are made on it such as if transformations are necessary (if any) to apply to the data.
- Information on the outputs, their format and location (if written to disk)
- Command-line interface or Python API documentation (depending on the interface with the package - I believe there is no CLI).
- A directory in the current repository (or better a separate one) containing Jupyter notebooks illustrating the usage in the data used for publication. Ideally these would reproduce at least the key figures/figure panels.
- Software packaging:
- Cloning from Github and installing with `pip` only instead of Conda is working fine (I tested) and would serve as a lightweight alternative for installation - the authors could also suggest this route to users.
- Ideally, please remove system-specific and packaging files from the distributed code (e.g. ".DS_Store", "build/", "CellNeighborEX.egg-info/", "dist", "CellNeighborEX/__pycache__"). These files should not be distributed and add to the size of the package when cloning. You may also want to remove these files from the git history using e.g. https://rtyley.github.io/bfg-repo-cleaner/ so that they aren't cloned as part of the history.
- It isn't clear why Python 3.11 is excluded from the compatible versions. By and large there are no reasons for this to be the case as all dependencies can be easily installed in 3.11 - I actually tried first with it as it is my default.

We would like to thank the reviewers for the thoughtful comments and constructive suggestions. Please find the point-to-point responses below.

Reviewer #1:

The authors have provided an improved version of their manuscript and all of my comments pertaining to the paper have been addressed. However, I am still not satisfied about the software package. I was able to install the python package without any issues. However, using it proved more challenging as I could not find any explicit instructions. I was able to find the scripts describing the analyses in the paper after some digging around in the repository. Although they are well structured and well commented, this is not a substitute for a documentation. Without more explicit instructions/vignette it will be challenging for others to use CelleighborEX. In 2023, it is to be expected that there are basic instructions on how to use a novel software package and this makes it much more valuable to the community.

Answer: As the reviewer suggested, we generated Python API documentation and tutorials. The materials are shown on this webpage: https://cellneighborex.readthedocs.io/en/latest/

Reviewer #3:

The authors have addressed all but one of my concerns. Unfortunately the data and software availability is still subpar and precluding both standard reproducibility practices and reducing the potential impact the paper may have in the community since without these features, it is impossible to run the software, assess its usability and evaluate its reproducibility and real-world use.

Answer: Thanks for the constructive comments. The followings are the answers for the comments.

The authors should attend to the following specific points:

- Data:

- The github repository only includes data on simulated datasets, and thus missing most data that for the basis of the method validation (Slide-seq and seqFISH).

Answer: The simulation datasets indicate pre-processed datasets used for analysis. To clarify it, we shared them via online open-access repository Figshare. Among the shared datasets, the seqFISH dataset is not included because it has been already pre-processed and published by Squidpy: https://squidpy.readthedocs.io/en/stable/api/squidpy.datasets.seqfish.html

- The authors should distribute all datasets used in the manuscript, ideally as a separate, referenceable repository with persistent identifiers (e.g. Zenodo).

Answer: As the reviewer suggested, we uploaded the datasets to Figshare:
https://figshare.com/articles/dataset/Datasets_for_neighbor-dependent_gene_expression_analysis_with_CellNeighborEX/24130395

- Documentation:

No documentation is provided beyond installation of the software. The authors should provide instructions on how to use their software to enable usage and adoption of the software. Specific points required:

- Basic usage (if possible including a demo/toy dataset)

Answer: We generated Python API documentation and tutorials. The materials are shown on this webpage: https://cellneighborex.readthedocs.io/en/latest/

- Information on what are the types of input for CellNeighborEX (e.g. h5ad), what assumptions are made on it such as if transformations are necessary (if any) to apply to the data.

Answer: We explained it in the tutorials:
https://cellneighborex.readthedocs.io/en/latest/tutorials.html

- Information on the outputs, their format and location (if written to disk)

Answer: We provided the information in the tutorials:
https://cellneighborex.readthedocs.io/en/latest/tutorials.html
Specifically, we mentioned it in the comments for the functions that generate output files.

- Command-line interface or Python API documentation (depending on the interface with the package - I believe there is no CLI).

Answer: As the reviewer suggested, we generated Python API documentation:
https://cellneighborex.readthedocs.io/en/latest/api.html

- A directory in the current repository (or better a separate one) containing Jupyter notebooks illustrating the usage in the data used for publication. Ideally these would reproduce at least the key figures/figure panels.

Answer: We presented the Jupyter notebooks in the following directory:
https://github.com/hkim240/CellNeighborEX/tree/main/docs/tutorials

- Software packaging:

- Cloning from Github and installing with `pip` only instead of Conda is working fine (I tested) and would serve as a lightweight alternative for installation - the authors could also suggest this route to users.

Answer: Thanks for the test. We described how to install CellNeighborEX on this webpage:
https://cellneighborex.readthedocs.io/en/latest/installation.html

- Ideally, please remove system-specific and packaging files from the distributed code (e.g. ".DS_Store", "build/", "CellNeighborEX.egg-info/", "dist", "CellNeighborEX/__pycache__"). These files should not be distributed and add to the size of the package when cloning. You may also want to remove these files from the git history using e.g. https://rtyley.github.io/bfg-repo-cleaner/ so that they aren't cloned as part of the history.

Answer: Thanks for the information. We removed the unnecessary files.

- It isn't clear why Python 3.11 is excluded from the compatible versions. By and large there are no reasons for this to be the case as all dependencies can be easily installed in 3.11 - I actually tried first with it as it is my default.

Answer: When we tried to install CellNeighborEX and its dependencies in Python 3.11, we could not install them because of numba. The error message says, "Could not find a version that satisfies the requirement numba==0.55.2". We confirmed that CellNeighborEX and its dependencies were successfully installed in Python version >=3.8, < 3.11. Accordingly, we proposed the installation methods.

18th Sep 2023

Manuscript number: MSB-2023-11670RR
Title: CellNeighborEX: Deciphering Neighbor-Dependent Gene Expression from Spatial Transcriptomics Data

Dear Dr Won,

Thank you again for sending us your revised manuscript. We have heard back from reviewer #3 who was asked to assess whether the documentation of CellNeighborEX allows wide adoption of the method by the community. We are glad to inform you that reviewer #3 is satisfied with the modifications made and supports publication. As such, I am pleased to inform you that we have accepted your manuscript for publication.

*** PLEASE NOTE *** As part of the EMBO Publications transparent editorial process initiative (see our Editorial at https://dx.doi.org/10.103/msb.2010.72), Molecular Systems Biology publishes online a Review Process File with each accepted manuscripts. This file will be published in conjunction with your paper and will include the anonymous referee reports, your point- by-point response and all pertinent correspondence relating to the manuscript. If you do NOT want this File to be published, please inform the editorial office at msb@embo.org within 14 days upon receipt of the present letter.

LICENSE AND PAYMENT:
All articles published in Molecular Systems Biology are fully open access: immediately and freely available to read, download and share.

Molecular Systems Biology charges an article processing charge (APC) to cover the publication costs. You, as the corresponding author for this manuscript, should have already received a quote with the article processing fee separately. Please let us know in case this quote has not been received.

Once your article is at Wiley for editorial production, you will receive an email from Wiley's Author Services system, which will ask you to log in and will present you with the publication license form for completion. Within the same system the publication fee can be paid by credit card, an invoice or pro forma can be requested.

Payment of the publication charge and the signed Open Access Agreement form must be received before the article can be published online.

Upon acceptance it is mandatory for you to return the completed payment form. Failure to send back the form may result in a delay in the publication of your paper.

Molecular Systems Biology articles are published under the Creative Commons licence CC BY, which facilitates the sharing of scientific information by reducing legal barriers, while mandating attribution of the source in accordance to standard scholarly practice.

Proofs will be forwarded to you within the next 2-3 weeks.

Thank you very much for submitting your work to Molecular Systems Biology.

Kind regards,

Maria

Maria Polychronidou, PhD
Senior Editor
Molecular Systems Biology

Reviewer #3:

Thank you for improving the documentation, packaging and instructions of CellNeighborEX. It really improves the ease and intuitiveness of use for future users.

EMBO Press Author Checklist

Corresponding Author Name: Kyoung Jae Won, Rajan Gogna
Journal Submitted to: Molecular Systems Biology
Manuscript Number: MSB-2023-11670

USEFUL LINKS FOR COMPLETING THIS FORM
The EMBO Journal - Author Guidelines
EMBO Reports - Author Guidelines
Molecular Systems Biology - Author Guidelines
EMBO Molecular Medicine - Author Guidelines

Reporting Checklist for Life Science Articles (updated January
This checklist is adapted from Materials Design Analysis Reporting (MDAR) Checklist for Authors. MDAR establishes a minimum set of requirements in transparent reporting in the life sciences (see Statement of Task: 10.31222/osf.io/9sm4x). Please follow the journal's guidelines in preparing your
Please note that a copy of this checklist will be published alongside your article.

Abridged guidelines for figures
1. Data
The data shown in figures should satisfy the following conditions:
- the data were obtained and processed according to the field's best practice and are presented to reflect the results of the experiments in an accurate and unbiased manner.
- ideally, figure panels should include only measurements that are directly comparable to each other and obtained with the same assay.
- plots include clearly labeled error bars for independent experiments and sample sizes. Unless justified, error bars should not be shown for technical
- if n<5, the individual data points from each experiment should be plotted. Any statistical test employed should be justified.
- Source Data should be included to report the data underlying figures according to the guidelines set out in the authorship guidelines on Data

2. Captions
Each figure caption should contain the following information, for each panel where they are relevant:
- a specification of the experimental system investigated (eg cell line, species name).
- the assay(s) and method(s) used to carry out the reported observations and measurements.
- an explicit mention of the biological and chemical entity(ies) that are being measured.
- an explicit mention of the biological and chemical entity(ies) that are altered/varied/perturbed in a controlled manner.
- the exact sample size (n) for each experimental group/condition, given as a number, not a range;
- a description of the sample collection allowing the reader to understand whether the samples represent technical or biological replicates (including how many animals, litters, cultures, etc.).
- a statement of how many times the experiment shown was independently replicated in the laboratory.
- definitions of statistical methods and measures:
  - common tests, such as t-test (please specify whether paired vs. unpaired), simple χ2 tests, Wilcoxon and Mann-Whitney tests, can be unambiguously identified by name only, but more complex techniques should be described in the methods section;
  - are tests one-sided or two-sided?
  - are there adjustments for multiple comparisons?
  - exact statistical test results, e.g., P values = x but not P values < x;
  - definition of 'center values' as median or average;
  - definition of error bars as s.d. or s.e.m.

Please complete ALL of the questions below.
Select "Not Applicable" only when the requested information is not relevant for your study.

Materials

Newly Created Materials	Information included in the manuscript?	In which section is the information available? (Reagents and Tools Table, Materials and Methods, Figures, Data Availability Section)
New materials and reagents need to be available; do any restrictions apply?	Not Applicable	

Antibodies	Information included in the manuscript?	In which section is the information available? (Reagents and Tools Table, Materials and Methods, Figures, Data Availability Section)
For **antibodies** provide the following information:<br>- Commercial antibodies: RRID (if possible) or supplier name, catalogue number and or/clone number<br>- Non-commercial: RRID or citation	Yes	Materials and Methods

DNA and RNA sequences	Information included in the manuscript?	In which section is the information available? (Reagents and Tools Table, Materials and Methods, Figures, Data Availability Section)
**Short novel DNA or RNA including primers, probes**: provide the sequences.	Yes	Materials and Methods

Cell materials	Information included in the manuscript?	In which section is the information available? (Reagents and Tools Table, Materials and Methods, Figures, Data Availability Section)
**Cell lines:** Provide species information, strain. Provide accession number in repository **OR** supplier name, catalog number, clone number, and/**OR** RRID.	Not Applicable	
**Primary cultures:** Provide species, strain, sex of origin, genetic modification status.	Yes	Materials and Methods
Report if the cell lines were recently **authenticated** (e.g., by STR profiling) and tested for mycoplasma contamination.	Not Applicable	

Experimental animals	Information included in the manuscript?	In which section is the information available? (Reagents and Tools Table, Materials and Methods, Figures, Data Availability Section)
**Laboratory animals or Model organisms:** Provide species, strain, sex, age, genetic modification status. Provide accession number in repository **OR** supplier name, catalog number, clone number, **OR** RRID.	Yes	Materials and Methods
**Animal observed in or captured from the field:** Provide species, sex, and age where possible.	Not Applicable	
Please detail **housing and husbandry conditions**.	Not Applicable	

Plants and microbes	Information included in the manuscript?	In which section is the information available? (Reagents and Tools Table, Materials and Methods, Figures, Data Availability Section)
**Plants:** provide species and strain, ecotype and cultivar where relevant, unique accession number if available, and source (including location for collected wild specimens).	Not Applicable	
**Microbes:** provide species and strain, unique accession number if available, and source.	Not Applicable	

Human research participants	Information included in the manuscript?	In which section is the information available? (Reagents and Tools Table, Materials and Methods, Figures, Data Availability Section)
If collected and within the bounds of privacy constraints report on age, sex and gender or ethnicity for all study participants.	Not Applicable	

Core facilities	Information included in the manuscript?	In which section is the information available? (Reagents and Tools Table, Materials and Methods, Figures, Data Availability Section)
If your work benefited from core facilities, was their service mentioned in the acknowledgments section?	Yes	Acknowledgments
	Not Applicable	

Design

Study protocol	Information included in the manuscript?	In which section is the information available? (Reagents and Tools Table, Materials and Methods, Figures, Data Availability Section)
If study protocol has been **pre-registered, provide DOI in the manuscript**. For clinical trials, provide the trial registration number **OR** cite DOI.	Not Applicable	
Report the **clinical trial registration number** (at ClinicalTrials.gov or equivalent), where applicable.	Not Applicable	

Laboratory protocol	Information included in the manuscript?	In which section is the information available? (Reagents and Tools Table, Materials and Methods, Figures, Data Availability Section)
Provide DOI OR other citation details if **external detailed step-by-step protocols** are available.	Not Applicable	

Experimental study design and statistics	Information included in the manuscript?	In which section is the information available? (Reagents and Tools Table, Materials and Methods, Figures, Data Availability Section)
Include a statement about **sample size** estimate even if no statistical methods were used.	Yes	Materials and Methods, Fig3 and FigEV4
Were any steps taken to minimize the effects of subjective bias when allocating animals/samples to treatment (e.g. **randomization procedure**)? If yes, have they been described?	Not Applicable	
Include a statement about **blinding** even if no blinding was done.	Not Applicable	
Describe **inclusion/exclusion criteria** if samples or animals were excluded from the analysis. Were the criteria pre-established?<br><br>If sample or data points were omitted from analysis, report if this was due to attrition or intentional exclusion and provide justification.	Not Applicable	
For every figure, are **statistical tests** justified as appropriate? Do the data meet the assumptions of the tests (e.g., normal distribution)? Describe any methods used to assess it. Is there an estimate of variation within each group of data? Is the variance similar between the groups that are being statistically compared?	Yes	Materials and Methods, Fig3 and FigEV4

Sample definition and in-laboratory replication	Information included in the manuscript?	In which section is the information available? (Reagents and Tools Table, Materials and Methods, Figures, Data Availability Section)
In the figure legends: state number of times the experiment was **replicated** in laboratory.	Yes	Materials and Methods, Fig3 and FigEV4
In the figure legends: define whether data describe **technical or biological replicates**.	Yes	Materials and Methods, Fig3 and FigEV4

Ethics

Ethics	Information included in the manuscript?	In which section is the information available? (Reagents and Tools Table, Materials and Methods, Figures, Data Availability Section)
Studies involving **human participants**: State details of **authority granting ethics approval** (IRB or equivalent committee(s), provide reference number for approval.	Not Applicable	
Studies involving **human participants**: Include a statement confirming that **informed consent** was obtained from all subjects and that the experiments conformed to the principles set out in the WMA Declaration of Helsinki and the Department of Health and Human Services Belmont Report.	Not Applicable	
Studies involving **human participants:** For publication of **patient photos**, include a statement confirming that consent to publish was obtained.	Not Applicable	
Studies involving experimental **animals**: State details of **authority granting ethics approval** (IRB or equivalent committee(s), provide reference number for approval. Include a statement of compliance with ethical regulations.	Yes	Materials and Methods
Studies involving **specimen and field samples:** State if relevant **permits** obtained, provide details of authority approving study; if none were required, explain why.	Not Applicable	

Dual Use Research of Concern (DURC)	Information included in the manuscript?	In which section is the information available? (Reagents and Tools Table, Materials and Methods, Figures, Data Availability Section)
Could your study fall under dual use research restrictions? Please check biosecurity documents and list of **select agents and toxins** (CDC): https://www.selectagents.gov/sat/list.htm	Not Applicable	
If you used a select agent, is the security level of the lab appropriate and reported in the manuscript?	Not Applicable	
If a study is subject to dual use research of concern regulations, is the name of the **authority granting approval and reference number** for the regulatory approval provided in the manuscript?	Not Applicable	

Reporting

The MDAR framework recommends adoption of discipline-specific guidelines, established and endorsed through community initiatives. Journals have their own policy about requiring specific guidelines and recommendations to complement MDAR.

Adherence to community standards	Information included in the manuscript?	In which section is the information available? (Reagents and Tools Table, Materials and Methods, Figures, Data Availability Section)
State if relevant guidelines or checklists (e.g., **ICMJE, MIBBI, ARRIVE, PRISMA**) have been followed or provided.	Not Applicable	
For **tumor marker prognostic studies,** we recommend that you follow the **REMARK** reporting guidelines (see link list at top right). See author guidelines, under ‘Reporting Guidelines’. Please confirm you have followed these guidelines.	Not Applicable	
For **phase II and III randomized controlled trials**, please refer to the **CONSORT** flow diagram (see link list at top right) and submit the CONSORT checklist (see link list at top right) with your submission. See author guidelines, under ‘Reporting Guidelines’. Please confirm you have submitted this list.	Not Applicable	

Data Availability

Data availability	Information included in the manuscript?	In which section is the information available? (Reagents and Tools Table, Materials and Methods, Figures, Data Availability Section)
Have **primary datasets** been deposited according to the journal's guidelines (see 'Data Deposition' section) and the respective accession numbers provided in the Data Availability Section?	Not Applicable	
Were **human clinical and genomic datasets** deposited in a public access-controlled repository in accordance to ethical obligations to the patients and to the applicable consent agreement?	Not Applicable	
Are **computational models** that are central and integral to a study available without restrictions in a machine-readable form? Were the relevant accession numbers or links provided?	Yes	Github
If publicly available data were reused, provide the respective **data citations in the reference list**.	Yes	In the reference
